# Synthesis and Antitrypanosomal Activity of Novel 2‑Nitroimidazole-3,5-Disubstituted Isoxazole Derivatives with Diaryl Ether and Thioether Substituents

**DOI:** 10.1021/acsomega.5c08312

**Published:** 2025-11-15

**Authors:** Larissa B. B. Santos, Diego B. Carvalho, Cristiane Y. K. Shiguemoto, Omar D. Lacerda, Talícia S. Silva, Gisele B. Portapilla, Saulo E. Silva-Filho, Palimécio G. Guerrero, Sérgio de Albuquerque, Adriano C. M. Baroni

**Affiliations:** † Laboratório de Síntese E Química Medicial (LASQUIM), Faculdade de Ciências Farmacêuticas, Alimentos E Nutrição (FACFAN), 54534 Universidade Federal de Mato Grosso Do Sul (UFMS), Avenida Costa E Silva, s.n. Bairro Universitário, Campo Grande, Mato Grosso Do Sul 79070-900, Brazil; ‡ Departamento de Análises Clínicas, Toxicológicas E Bromatológicas, Faculdade de Ciências Farmacêuticas de Ribeirão Preto, Universidade de São Paulo (USP), Avenida Do Café, s.n. Monte Alegre, Ribeirão Preto, São Paulo 14040-903, Brazil; § Faculdade de Ciências Farmacêuticas, Alimentos E Nutrição (FACFAN), Universidade Federal de Mato Grosso Do Sul (UFMS), Avenida Costa E Silva, s.n. Bairro Universitário, Campo Grande, Mato Grosso Do Sul 79070-900, Brazil; ∥ Universidade Tecnológica Federal Do Paraná, Química, Rua Deputado Heitor Alencar Furtado, 4900, Curitiba, Paraná 81280-340, Brazil

## Abstract

In this study, we synthesized 17 novel 2-nitroimidazole-3,5-disubstituted isoxazole analogs of benznidazole with diaryl ether and thioether substituents and evaluated their antitrypanosomal activity and cytotoxicity. Compounds **6a**–**q** were obtained in moderate-to-good yields (29–81%). Derivatives **6a**–**g** displayed greater activity than benznidazole. **6b** (*R* = Ph-O-(4-F-Ph)) and **6g** (*R* = Ph-O-(4-OCH_3_-Ph)) were the most active of all the synthesized compounds (IC_50_ = 0.50 μM and IC_50_ = 0.64 μM, respectively). Compounds **6h**–**n** included a 3-fluoro substituent in the first phenyl ring, resulting in lower activity. The diaryl thioether compounds **6o**–**q** exhibited lower anti-*T. cruzi* activity than diarylether analogs.

## Graphical abstract

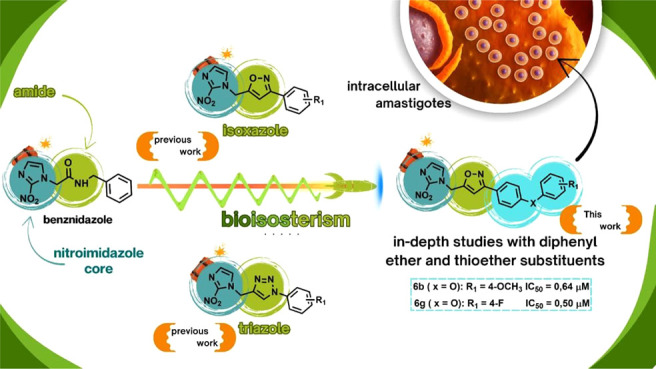

## Introduction

Chagas disease (CD), also known as American Trypanosomiasis, is a tropical illness caused by *Trypanosoma cruzi*, a Trypanosomatidae protozoan. While the most common form of infection with *T. cruzi* is through vectorial transmission, which occurs through contact with the feces of a triatomine insect, other important means of infection include infected blood transfusion, oral transmission through contaminated food, congenital transmission and organ transplantation.^1,2^

According to the World Health Organization (WHO), it is estimated that there are approximately 6 to 7 million people infected worldwide, making it a major public health problem, especially in Latin American countries where the disease is endemic.^3^ The disease also has a significant impact on the social and economic well-being of the poverty-stricken communities it mainly affects, classifying it as a neglected tropical disease.^4^ Although Chagas disease is widespread in Latin America, migratory patterns have disseminated it worldwide, with cases confirmed in Europe, the United States, Japan and Australia, making it a worldwide health problem.^5^

Currently, therapeutic options for the treatment of Chagas disease are limited to two nitroheterocycle drugs, benznidazole (BZ) **1** and nifurtimox **2** (Figure 1). Despite their poor safety and effectiveness profiles, these have been the primary treatment for nearly 50 years.^6,7^ Both drugs are most effective during the acute phase of *T. cruzi* infection, present better tolerance in children while showing higher toxicity in adults and varied susceptibility among *T. cruzi* strains. Nifurtimox **2** is a nitrofuran derivative that was initially introduced in 1969; however, it is poorly tolerated in adult patients, with low treatment adherence due to the drug’s numerous adverse effects. These include gastrointestinal symptoms such as anorexia, vomiting, and diarrhea, neurological disorders including dizziness, headaches, mood swings, disorientation, and insomnia, as well as fatigue and skin rashes.^8,9^

**Figure 1:**
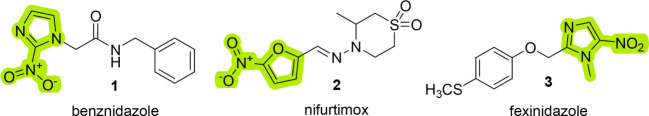
Antitrypanosomal agents benznidazole **1**, nifurtimox **2** and fexinidazole **3**.

Benznidazole **1** is a 2-nitroimidazole derivative and is currently the first line of treatment for Chagas disease demonstrating a similar efficacy profile to that of Nifurtimox. Although its safety and tolerability are slightly better than those of nifurtimox, adverse events such as allergic dermopathy, vomiting, nausea, peripheral polyneuropathy and even bone marrow depression have been reported after treatment.^10^ Due to these adverse events, patient adherence is often compromised, emphasizing the need for the discovery of new, more effective treatments for Chagas disease with fewer adverse effects.^11^

Nitroheterocycle compounds exhibit a wide range of biological activities, including antibacterial, antituberculosis, and antiparasitic properties, and have emerged as promising therapeutic options for treating neglected diseases.^12,13^ Besides benznidazole **1** and nifurtimox **2**, other nitroheterocycle compounds have demonstrated significant antitrypanosomal activity. For instance, fexinidazole **3**, a 5-nitroimidazole compound, was developed by The Drugs for Neglected Diseases initiative (DNDi) and was approved for the treatment of human African trypanosomiasis (Figure 1).^14^

Given the reemergence of nitroheterocycles as effective therapeutic options in the treatment of neglected diseases, our research group devised and synthesized a series of triazole compounds **4** based on the structure of benznidazole **1**. The study confirmed previous findings by demonstrating that bioisosteric replacement of the amide group with a 1,2,3-triazole ring resulted in compounds with good antitrypanosomal activity (Figure 2).^15–17^ Assunção et al.^15^ reported notable antitrypanosomal activity of triazole derivatives bearing a diaryl ether group, such as compound **4b** (R^1^ = Ph–O–Ph), which exhibited an IC_50_ value of 6.6 μM against *T. cruzi* amastigote forms.

**Figure 2:**
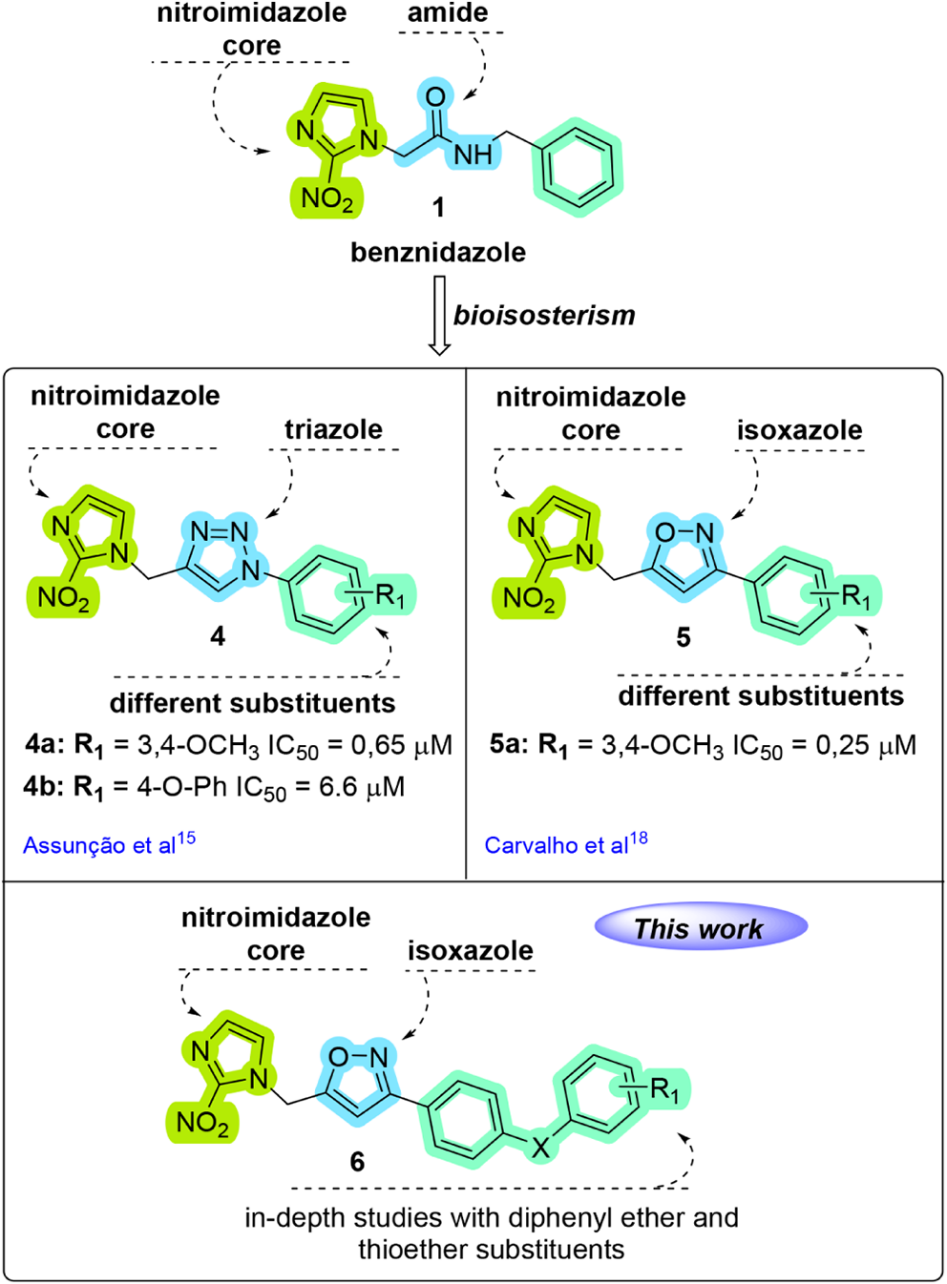
Design of 2-nitroimidazole-3,5-disubstituted isoxazole analogues **6** based on the structure of antitrypanosomal drug benznidazole **1**.

Based on these promising results, the isoxazole scaffold was used in another study conducted by our research group.^18^ The isoxazole moiety is also well-known for its several biological activities and has been widely employed in the development of numerous therapeutic agents, including antibacterial, anticancer, anti-inflammatory, antiviral, and antiparasitic compounds.^19^ As a result, in novel benznidazole derivatives, a bioisosteric substitution of the amide group by an isoxazole core was proposed, and the outcomes revealed that isoxazole compounds **5** exhibit higher activity when compared to triazole **4** compounds with similar substituents (Figure 2).^18^

Inspired by this result, this study reports the synthesis of a new series of nitroimidazole isoxazole compounds with diaryl ether and diaryl thioether substituents with design based on the structure of benznidazole **1** and **4b,** and additionally, the antitrypanosomal activity and cytotoxicity of these compounds were also evaluated (Figure 2).

## Results and Discussion

### Chemistry

Nitroimidazole-isoxazole derivatives were synthesized via a [3 + 2] cycloaddition reaction between propargyl-2-nitroimidazole and various diaryl ether or thioether chloro-oximes bearing different substituents. The first building block, propargyl-2-nitroimidazole, was obtained through a multistep synthetic route beginning with the condensation of S-methylisothiourea hemisulfate and aminoacetaldehyde dimethyl acetal to yield 2-aminoimidazole **9**.^20^ This compound was then subjected to diazotization and nitration to afford 2-nitroimidazole **10**,^18,21^ which was subsequently alkylated with propargyl bromide **11** to afford propargyl 2-nitroimidazole **12** (Scheme 1).^18,22^

**Scheme 1:**
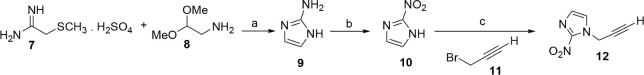
Synthesis of Propargyl-2-nitroimidazole **12** ^a^

a Reagents and conditions: (a) H_2_O, 75 °C, 1 h, (61%). (b) (1) H_2_O, – 5 °C, HBF_4_. (2) NaNO_2_, – 10 °C, 30 min. (3) CuSO_4_·5H_2_O, r.t., 24 h (86%). (c) K_2_CO_3_, acetone, r.t. 24 h (84%).

Chloro-oximes were synthesized through two distinct methodologies in a sequential synthetic pathway, utilizing either aldoximes **16** ^23,24^ or amidoximes **20**,^25,26^ as illustrated in Scheme 2. The reaction of aldoximes **16a–h**, **k–n**, **q** with N-chlorosuccinimide (NCS) afforded the corresponding chloro-oximes **17a–h**, **k–l**, **n**.^27^ Conversely, amidoximes **20i–j**, **o–p** underwent chlorination with hydrochloric acid (HCl), yielding chloro-oximes **17i–j**, **o–p**, respectively.^28^

**Scheme 2:**
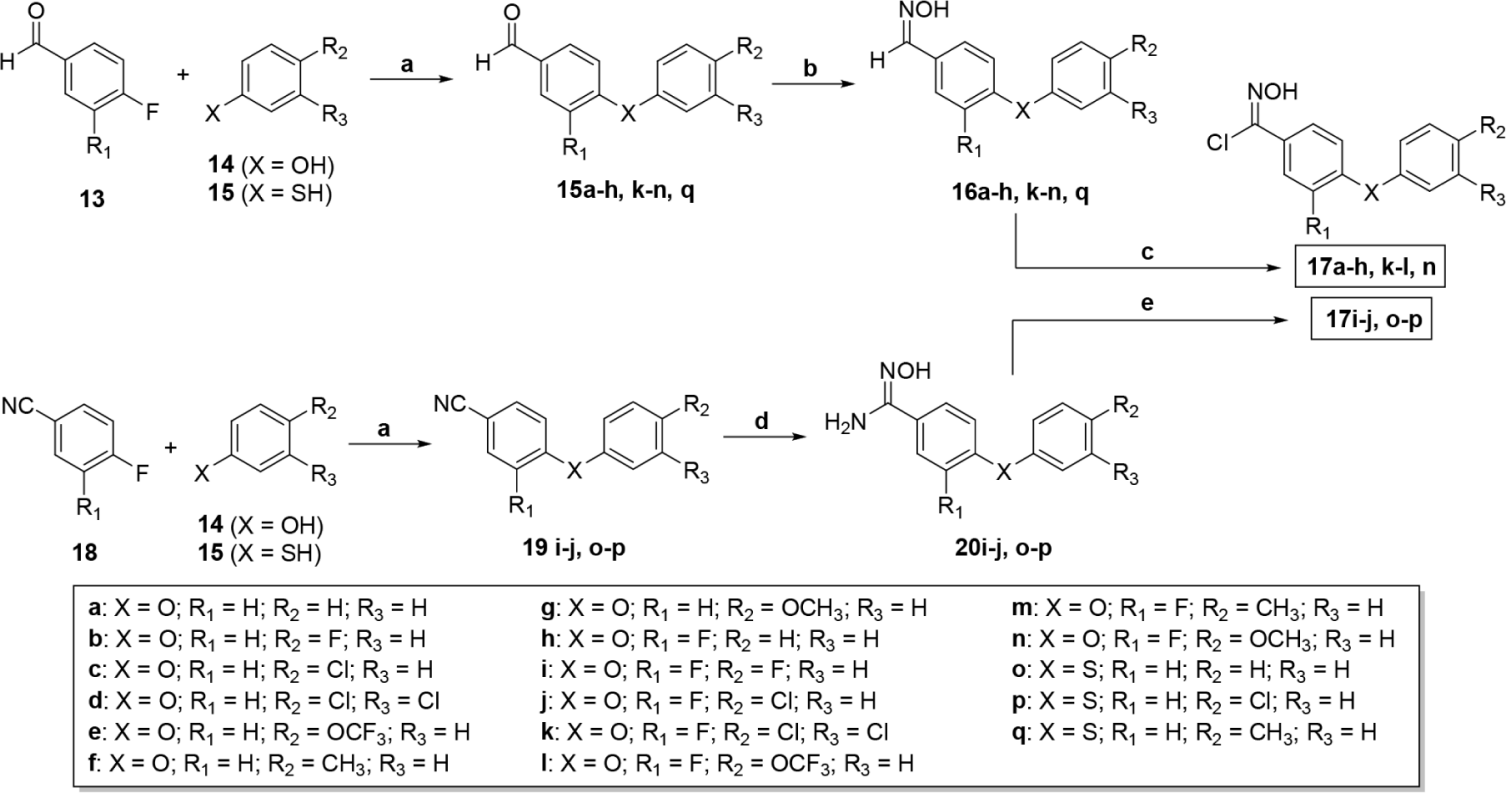
General Procedure for the Synthesis of Chloro-Oxime **17** [Fn sch2-fn1]

i Reagents and conditions: (a) K_2_CO_3_, DMSO, reflux, 110 °C (53–98%). (b) NH_2_OH·Cl, NaOH, EtOH/H_2_O, r.t. 3 h (48–97%). (c) NCS, CH_3_CN, DMF, 60 °C, 2 h (33–83%). (d) NH_2_OH·HCl, K_2_CO_3_, MeOH, reflux, 2 h (74–89%). (e) H_2_O, HCl, NaNO_2_, r.t., 2 h (23–61%).

Subsequently, reaction was performed between chloro-oximes **17a-l, n-p** and propargyl-2-nitroimidazole **12**, using a catalytic system of CuSO_4_·5H_2_O, sodium ascorbate, and KHCO_3_, in a solvent mixture of THF/CH_2_Cl_2_ 1:1 yielding 2-nitroimidazole isoxazoles **6a-l, n-p** in moderate to good yields (29–81%) (Scheme 3).^29^

**Scheme 3:**
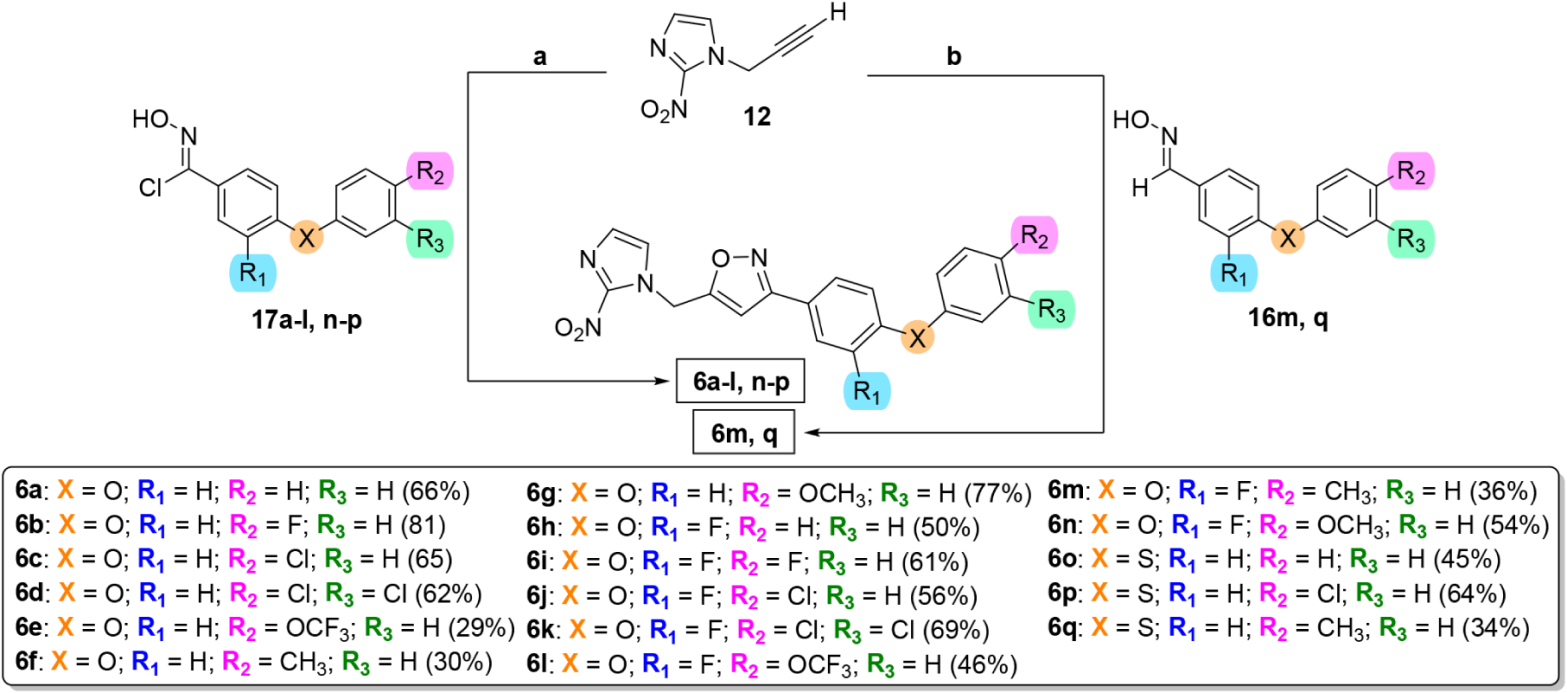
Synthesis of 2-Nitroimidazole Isoxazoles Derivatives **6** ^a^

a Reagents and conditions: (a) CuSO_4_·5H_2_O, sodium ascorbate, KHCO_3_, CH_2_Cl_2_/THF, r.t., 48 h. (b) (1) NCS, DMF, −10 °C, then r.t., 2 h. (2) DBU, propargyl-2-nitroimidazole **12**, r.t., 8h.

A one-pot protocol was employed to obtain compounds **6m, q** using DBU (1,8-Diazabicyclo[5.4.0]­undec-7-ene) to avoid the use of metallic catalysts and minimize purification steps.^30^ In this case, chloro-oxime were generated in situ by treating aldoxime **16m**, **q** with NCS, followed by sequential addition of DBU and terminal acetylene **12**. The 2-nitroimidazole isoxazole compounds **6m**, **q** were obtained with 36% and 34% yields, respectively (Scheme 3).

To evaluate the impact of fluorine substitution on antitrypanosomal activity, compounds **6h–n** were designed with fluorinated phenyl rings, given fluorine’s known influence on key physicochemical properties, such as solubility, lipophilicity, p*K*_a_, and molecular conformation which may affect pharmacokinetics, potency, selectivity, and toxicity.^31–33^

Furthermore, compounds **6o–q** were developed via bioisosteric replacement of the amide group in benznidazole with a heterocyclic isoxazole ring, incorporating 4-phenylthiobenzene substituents. This design aimed to explore a novel class of sulfur-containing compounds by substituting phenoxybenzene group, previously shown to be active against *T. cruzi* amastigotes in 1,2,3-triazole derivatives, with its bioisostere, phenylthiobenzene.^15,17^ Given the classical bioisosterism between oxygen and sulfur, this modification may preserve biological activity while potentially enhancing pharmacological properties and reducing toxicity.^34^

### In Vitro Antitrypanosomal Activity, Cytotoxicity, and SAR

Table 1 displays the in vitro antitrypanosomal activity against *T. cruzi* amastigotes (Tulahuen lacZ strain). Drug discovery experts consider that a hit compound for CD should exhibit a half-maximal inhibitory concentration (IC_50_) of <10 μM against the intracellular forms of the parasite.^35^ In this study, we report a novel series comprising 17 compounds of which 14 showed potencies below 10 μM, while 12 demonstrated greater activity than the reference drug benznidazole (BZ). Compounds were categorized as active (IC_50_ < 4.0 μM), moderately active (IC_50_ 4.0–60.0 μM), or inactive (IC_50_ > 60.0 μM). The cytotoxicity evaluation and the determination of the selectivity index (SI) were done on LLC-MK2 cells.

**Table 1:**
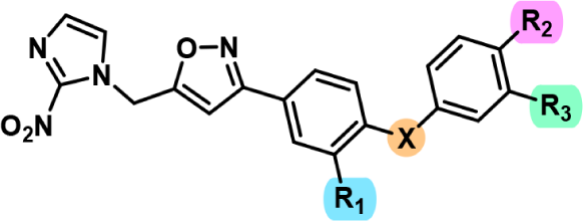
Antitrypanosomal Activity and Cytotoxicity of Compounds Type **6**

Comp.	R_1_	R_2_	R_3_	Log*P* ^a^	Hansch constant^b^	Hammett constant^c^		Amastigote IC_50_ (μM ± SD)^d^	LLC-MK2 CC_50_ (μM **±** SD)^e^	SI^f^
					π	σ_p_	σ_m_			
**6a (X = O)**	H	H	H	4.02	0.00	0.00	-	1.22 ± 0.73	>100	>82.2
**6b (X = O)**	H	F	H	4.18	0.14	0.06	-	0.50 ± 0.51	>100	>199.4
**6c (X = O)**	H	Cl	H	4.70	0.71	0.23	-	1.01 ± 1.13	>100	>99.0
**6d (X = O)**	H	Cl	Cl	5.30	1.25	0.23	-	2.79 ± 0.93	>100	>35.9
**6e (X = O)**	H	OCF_3_	H	4.99	1.04	0.35	-	1.25 ± 0.71	>100	>80.1
**6f (X = O)**	H	CH_3_	H	4.47	0.56	–0.17	-	1.77 ± 0.24	>100	>56.5
**6g (X = O)**	H	OCH_3_	H	4.08	–0.02	–0.27	-	0.64 ± 0.05	>100	>155.3
**6h (X = O)**	F	H	H	4.11	0.00	0.00	-	3.18 ± 2.18	>100	>31.4
**6i (X = O)**	F	F	H	4.27	0.14	0.06	-	1.42 ± 0.42	>100	>70.4
**6j (X = O)**	F	Cl	H	4.79	0.71	0.23	-	1.56 ± 0.69	>100	>64.1
**6k (X = O)**	F	Cl	Cl	5.39	1.25	0.23	0.37	8.50 ± 2.06	>100	>11.8
**6l (X = O)**	F	OCF_3_	H	5.08	1.04	0.35	-	3.77 ± 0.07	55,6 ± 4.4	>14.7
**6m (X = O)**	F	CH_3_	H	4.56	0.56	–0.17	-	7.41 ± 3.22	>100	>13.5
**6n (X = O)**	F	OCH_3_	H	4.17	–0.02	–0.27	-	3.54 ± 0.21	>100	>28.2
**6o (X = S)**	H	H	H	4.23	0.00	0.00	-	20.55 ± 0.98	>100	>4.9
**6p (X = S)**	H	Cl	H	4.91	0.71	0.23	-	11.09 ± 0.16	>100	>9.0
**6q (X = S)**	H	CH_3_	H	4.68	0.56	–0.17	-	17.14 ± 0.24	>100	>5.8
**BZN**				0.78	-	-	-	5.67 ± 0.83	>100	>17.6

a Log*P* calculated using Molinspiration software.

b Hansch’s constant (Σπ observed).^36,41^

c Hammett’s constant σ.^36,41^

d Half-maximum inhibitory concentration (IC_50_) of *T. cruzi* intracellular amastigotes (Tulahuen strain).

e Cytotoxic concentration (CC_50_) on LLC-MK2 cells.

f SI, selectivity index: CC_50_ of LLC-MK2 cells/IC_50_ of intracellular amastigotes. SD: standard deviation. Benznidazole (BZN) as reference drug.

To design the structure of these novel isoxazole compounds, we employed traditional medicinal chemistry approaches,^36^ such as bioisosterism and Craig Plot analysis. The compounds were prepared with varying Hansch hydrophobicity constants (π) and Hammett substituent constants (σ) of the substituents. This allowed us to better understand the role of each substituent group in the antitrypanosomal activity of this novel series of 2-nitroimidazole isoxazoles (**6a**–**q**) with diaryl ether or thioether groups.

The first synthesized compound of the diaryl ether series **6a** (*R* = Ph-O-Ph) presented an impressive IC_50_ of 1.22 μM, being 4.6-fold more active than the reference drug benznidazole (BZN) (IC_50_ of 5.67 μM). **6a** also exhibited a significantly higher SI of 82.2, which indicates the compound has a higher selectivity for parasites rather than mammalian cells. When comparing the structure of BZN and **6a**, as well as the other synthesized compounds in this series, BZN has a Log*P* of only 0.78 while **6a** has a much greater Log*P* of 4.02, as a result of the presence of two phenyl rings in its structure. Other than the bioisosteric replacement of the amide group in BZN for an isoxazole ring, the increased Log*P* may be a crucial factor for better activity of **6a** as well as the other compounds in this series because lipophilicity is an important physical–chemical property that affects a compound’s cell permeability, especially when its targets are intracellular amastigote forms.

Although this isoxazole compound is unpublished in literature, it can be compared to its triazole congener with the same diphenyl ether substituent, which presented a promising activity with IC_50_ of 6.6 μM and a SI of 18.3. Our isoxazole analog **6a** was 5.4-fold more active against amastigote forms than its triazole analog.^15^ Despite the structural and electronic similarities between 1,2,3-triazole and 3,5-isoxazole rings, a tendency of better activity of isoxazoles when compared to triazoles with the same substitution patterns has already been reported.^18,37^

Next, we evaluated compounds containing lipophilic and electron-withdrawing substituents (π+, σ+). **6b** (*R* = Ph-O-(4-F-Ph)) was the compound with the highest potency of all the synthesized compounds of this work with an IC_50_ of 0.5 μM. Moreover, **6b** also showed the highest safety profile with an excellent SI of 199.4. With a 4-chloro substituent, **6c** (*R* = Ph-O-(4-Cl-Ph)) showed the higher potency of the chlorine group when compared to its unsubstituted analog **6a,** exhibiting good activity with an IC_50_ of 1.01 μM and SI of 99.0. However, **6c** was 2-fold less active than its fluoro-substituted analog **6b**. When comparing these two substituent’s parameters, analog **6c** with a 4-Cl group (π = 0.71; σ = 0.23) exhibited significantly higher lipophilic and electron-withdrawing properties than **6b** (π = 0.14; σ = 0.06).

Compound **6d** (*R* = Ph-O-(3,4-*di*-Cl-Ph)) had the addition of another chlorine substituent in the *meta* position that resulted in a reduction of antitrypanosomal activity (IC_50_ = 2.79 μM) although it was still considered active, compared to analog **6c** bearing only one chlorine substituent (IC_50_ = 1.01 μM). This reduction in activity may be due to the significantly higher lipophilicity profile (Log *p* = 5.30; π = 1.25) along with the increased electron-withdrawing effect and, perhaps, an unfavorable steric hindrance. The addition of an extra chlorine in *meta* position did not boost antitrypanosomal activity and a considerable reduction of the selectivity index of **6d** (SI = 35.9) was also observed in comparison to *para-*substituted **6c** (SI = 99.0).

In sequence, compound **6e** (*R* = Ph-O-(4-OCF_3_–Ph)) also with considerable lipophilic and electron-withdrawing properties (π+, σ+) exhibited good activity with an IC_50_ = 1.25 μM and SI = 80.1.

In our synthesized compounds we observed an inversely proportional relationship between antitrypanosomal activity and Log P within π+, σ+ compounds, following the sequence from the most to least active: **6b** (*R* = 4-F-Ph, Log*P* = 4.18) > **6c** (*R* = 4-Cl-Ph, Log*P*= 4.70) > **6e** (*R* = 4-OCF_3_–Ph, Log*P* = 4.99) > **6d** (*R* = 3,4-*di*-Cl-Ph, Log*P* = 5.30). This suggests that there may be an ideal range of Log P for this set of compounds. Higher Log *p* values in this series tend to decrease the compounds’ permeability, leading to lower potency.

Compound **6f** (*R* = Ph-O-(4-CH_3_Ph)), possessing lipophilic and electron-donating properties (π = 0.56, σ = −0.17), was evaluated and showed good activity (IC_50_ = 1.77 μM). However, it was less active than compound **6a** (R1 = Ph). Subsequently, compound **6g** (*R* = Ph-O-(4-OCH_3_Ph)), with slightly hydrophilic and electron-donating properties (π = −0.02, σ = −0.27), demonstrated significant antitrypanosomal activity with an IC_50_ = 0.64 μM. Compound **6g** was the second most potent of all the synthesized compounds, and exhibited the second highest selectivity against *T. cruzi* (SI = 155.3).

Compounds **6a**–**g** were all considered active and more potent than reference drug benznidazole. This series of diaryl ether substituted compounds was also more potent when compared to similar 2-nitroimidazole isoxazole compounds previously published by our research group, without diaryl ether substituents.^18^

Analogs **6b** (*R* = Ph-O-(4-F-Ph)) and **6g** (*R* = Ph-O-(4-OCH_3_Ph)) were highlighted as the most active of this work which may be due to the ability of these substituents to perform hydrogen bonds. Furthermore, the 4-F substituent in **6b** may establish dipole–dipole interactions with amino acid residues, such as carbonyl from amide groups, which may justify its greater potency in comparison to its synthesized congeners. It should also be noted that the fluorinated compound **6b** is more resistant to metabolism than compounds **6a** and **6g**, demonstrating its potential for in vivo studies.^32^

For the (3-F)-Ph-O-Ph series, compounds **6h**–**n** were synthesized with similar substituents as, but with **6a**–**g** the addition of a 3-fluoro substituent in the first ring of the diaryl ether group. This modification was made to reduce potential metabolism at this vulnerable position and to investigate whether the presence of fluorine in this position could enhance the potency of these compounds due to a potentially more active conformation.

The first compound of this series, **6h** (*R* = (3-F-Ph)-O-(4-Ph)), was considered active with an IC_50_ of 3.18 μM but presented a low selectivity index of 31.4. In comparison with the similar analog **6a** (*R* = Ph-O-Ph, IC_50_ = 1.22 μM, SI = 82.2), **6h** displayed lower potency. This same pattern was observed in the other synthesized compounds of this series **6i**–**6n**, where (3-F)-substituted analogs showed considerably lower activity and selectivity than their congeners without a fluoro substituent.

Compounds **6i**–**l**, which have lipophilic and electron-withdrawing substituents (π+, σ+), were sequentially evaluated and found to be active with IC_50_ values below 4 μM, except for **6k**, which exhibited moderate activity and low selectivity (IC_50_ = 8.50 μM, SI = 11.8), likely due to its high Log P. Compounds **6i** (*R* = (3-F-Ph)-O-(4-F-Ph)) and **6j** (*R* = (3-F-Ph)-O-(4-Cl-Ph)), showed the best activity in this entire series with IC_50_ = 1.42 μM, SI = 70.4 and IC_50_ = 1.56 μM, SI = 64.1, respectively. Nevertheless, **6i** was considerably less active (2.8-fold) than its congener **6b** (*R* = Ph-O-(4-F-Ph)); the same was observed when comparing **6c** (*R* = (Ph-O-(4-Cl-Ph) and **6j** (R_1_ = (3-F-Ph)-O-(4-Cl-Ph)).

Analogously **6m** (*R* = (3-F-Ph)-O-(4-CH_3_–Ph)) with lipophilic and electron-donating properties (π + , σ-) was also 4-fold less active than **6f** (*R* = Ph-O-(4-CH_3_Ph)). Likewise, with hydrophilic and electron-donating properties (π-, σ-), **6n** (*R* = (3-F-Ph)-O-(4-OCH_3_–Ph) exhibited 5.5-fold less activity and considerably lower SI than similar analog **6g** (*R* = Ph-O-(4-OCH_3_–Ph), which was the second most active of all the synthesized compounds.

Overall, the (3-F)-diphenyl ether compounds did not outperform their respective unsubstituted congeners or exhibit a superior safety profile. Despite maintaining the same substituents in the second phenyl ring, the addition of the (3-F)-substituent in the first phenyl ring evidently did not enhance antitrypanosomal activity.

Regarding diaryl thioether substituted isoxazoles **6o**–**q**, these compounds exhibited the lowest antitrypanosomal activity among all the synthesized compounds in this study, besides presenting the lowest selectivity indices. Compound **6o** (*R* = Ph-S-Ph) showed moderate activity with an IC_50_ of 20.55 μM and a very low selectivity index of 4.9. Compound **6p** (*R* = Ph-S-(4-Cl-Ph)), with a lipophilic and electron-withdrawing substituent (π+, σ+), showed improved activity (IC_50_ = 11.09 μM) compared to the unsubstituted **6o**, yet the toxicity profile remained unsatisfactory (SI = 9.0). Regarding compound **6q** (*R* = Ph-S-(4-CH_3_–Ph)), with lipophilic and electron-donating properties (π+, σ-), this analog exhibited moderate activity with an IC_50_ of 17.14 μM, while also displaying an unsatisfactory selectivity index of 5.8.

The results indicate that, although oxygen and sulfur atoms are traditionally regarded as classical bioisosteres, this structural analogy did not yield equivalent biological activity within the series of 2-nitroimidazole-isoxazole derivatives tested against *Trypanosoma cruzi* amastigote forms. We hypothesize that this discrepancy arises from the distinct ways in which these heteroatoms interact with biological targets. Sulfur, particularly when bonded to aryl or heteroaryl groups, is capable of engaging in sigma-hole interactions, which enable a broad spectrum of interactions and accommodate a wider range of bond angles.^38^ Conversely, oxygen may act as a more effective hydrogen bond acceptor in a molecular recognition processes involving the parasite’s therapeutic target, potentially contributing to the superior antitrypanosomal activity observed in the diaryl ether derivatives.^39^ Moreover, this tendency of the ether link in enhancing antitrypanosomal activity was also demonstrated in Papadopoulos’s study,^40^ further supporting our findings in the present work.

### SAR Highlights of 2-Nitroimidazole-3,5-Disubstituted Isoxazole with Diaryl Ether and Thioether Substituents

The results of antitripanosomatid activity of 2-nitroimidazol-3,5-disubstituted isoxazole diaryl ether and thiother substituents indicates the following SAR highlights (Figure 3):

The 2-nitroimidazole isoxazole diphenyl ether **6a** showed an IC_50_ of 1.22 μM, exhibiting greater activity compared to the triazole counterpart reported by Assunção et al., which presented IC_50_ value of 6.6 μM;^15^
The diaryl ether substituents are more active than the diaryl thiother (**6a** > **6o**, **6c** > **6p**, **6f** > **6q**);
Fluorine at 3-position in the first ring (R_1_ substituent) did not improve the potency. However, when fluorine is located at 4-position in the second ring (R_2_ substituent), the results were better (**6b** > **6h**, **6c** > **6j**, **6d** > **6k**, **6e** > **6l**, **6f** > **6m**, **6g** > **6n**).

**Figure 3:**
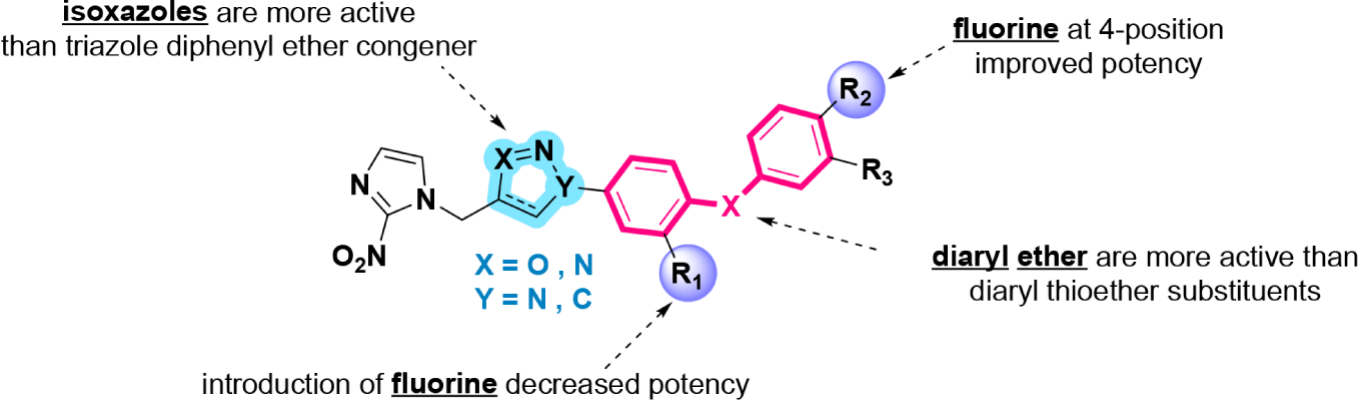
SAR highlights for 2-nitroimidazole-3,5-disubstituted isoxazole derivatives with diaryl ether and thioether substituents.

## Conclusion

A novel series of 3,5-isoxazole-2-nitroimidazole analogs was successfully synthesized in moderate to good yields (29%–81%) via a 1,3-dipolar cycloaddition reaction, affording 17 previously unreported isoxazole derivatives containing diaryl ether and thioether substituents. The molecular design was based on the bioisosteric replacement of the amide group in benznidazole with an isoxazole ring, and further functionalized with diaryl ether and thioether groups bearing substituents that vary in Hansch’s hydrophobicity constant (π) and Hammett’s electronic parameter (σ).

Biological evaluation revealed that 12 compounds exhibited higher antitrypanosomal potency and selectivity against the amastigote forms of *Trypanosoma cruzi* compared to benznidazole, alongside improved lipophilicity profiles. Notably, compounds **6a–g** were more active than the reference drug, with **6b** (*R* = Ph-O-(4-F-Ph)) and **6g** (*R* = Ph-O-(4-OCH_3_-Ph)) showing the highest potency, with IC_50_ values of 0.50 μM and 0.64 μM, respectively.

In contrast, compounds **6h–n**, bearing a meta-fluorine on the first phenyl ring of the diaryl ether, did not show enhanced activity. Similarly, replacement of the ether linkage with sulfur to form diaryl thioether analogs did not improve potency, despite oxygen and sulfur being classical bioisosteres. These thioether derivatives **6o**–**q** showed only moderate activity (IC_50_ = 11.0–20.55 μM).

Among the compounds tested, derivative **6b** stands out as a promising lead for further *in vivo* studies, combining potent antitrypanosomal activity with favorable physicochemical properties, suggesting reduced metabolic liability and improved pharmacokinetic behavior.

## Experimental Section

### Materials and Methods

Anhydrous solvents were distilled and removed the water before the use, according to the standard procedure.^42^ Chemicals used for the synthesis were reagent grade and they were used without purification. Reactions were carried out under a nitrogen atmosphere and monitored by thin layer chromatography (TLC) using prepared plates (silica gel 60 GF_254_ on aluminum). TLC plates were examined under 254 and 360 nm ultraviolet lights or with the developing agent ethanolic vanillin and heat. Flash column chromatography was performed on silica gel 60 (particle size 200–400 mesh ASTM, purchased from Sigma-Aldrich). Melting points were determined on Fisatom 430D equipment. The ^1^H and ^13^C NMR spectra were recorded at 300 or 500 and 75 or 100 MHz, respectively, using Bruker DPX 300 NMR Spectrometer or Bruker Avance Neo 500 Nuclear Magnetic Ressonance Spectrometer. CDCl_3_ and DMSO-*d*_*6*_ were used as solvent. Chemical shifts (δ) are expressed as parts per million (ppm) downfield from tetramethylsilane or the residual hydrogen signals of the deuterated solvent (CDCl_3_^1^H δ 7.27 and ^13^C δ 77.0 ppm; DMSO-d_*6*_^1^H δ 2.5 and ^13^C δ 39.51 ppm) as the internal standard. High-resolution mass spectrometry (HRMS) data were obtained on a mass spectrometer MicrOTOF-Q III (Bruker Daltonics, Billerica, MA, USA) equipped with electrospray ionization source and analyzers quadrupole and Time-Of-Flight (QTOF). The samples (0.5 mg/mL) were analyzed in positive ionization mode and the molecular formulas were determined by the accurate mass considering errors up ±5 ppm and mSigma below 30.

### Synthesis of 2-Nitroimidazole Isoxazoles **6a-l**, **n-p**

#### General Procedure A^29^

To a solution of the respective chloro-oxime of interest **17** (3 mmol, 1.0 equiv) and the propargyl-2-nitroimidazol **12** (3.3 mmol, 1.1 equiv) in CH_2_Cl_2_/THF (50:50, 5 mL/mmol), CuSO_4_.5H_2_O (0.19 mmol), sodium ascorbate (0.52 mmol) and KHCO_3_ (30 mmol, 10 equiv) were added. The mixture was stirred at room temperature for 48 h. Extraction was carried out using ethyl acetate (3 × 30 mL) and rinsed with NaCl saturated solution (3 × 30 mL). The solvent was removed under low pressure after the organic phase was dried over anhydrous MgSO_4_. The product was purified using flash colunm chromatography (*n*-hexane:ethyl acetate, 6:4 (v/v)) and was then subjected to recrystallization in ethanol.

#### General Procedure B^30^

*N*-chlorosuccinimide (3.6 mmol, 1.2 equiv) was added to a stirred solution of aldoximes **16** (3 mmol, 1 equiv) in DMF (10 mL) at −10 °C and warmed until it reached room temperature, then the reaction was stirred for 2 h. The reaction was then agitated for 8 h after DBU (3 mmol, 1 equiv) and propargyl-2-nitroimidazole **12** (3.6 mmol, 1.2 equiv) were added. After the reaction was completed, as verified by TLC, water (50 mL) was added and the product was extracted with ethyl acetate (3 × 30 mL). The organic phase was recovered and dried using anhydrous MgSO_4_, the solvent was evaporated under reduced pressure. The product was purified using flash colunm chromatography (*n*-hexane: ethyl acetate, 6:4 (v/v)) and was then subjected to recrystallization in ethanol.

##### 5-((2-Nitro-1H-imidazol-1-yl)­methyl)-3-(4-phenoxyphenyl)­isoxazole (**6a**)

Reaction of propargyl-2-nitroimidazol **12** (498.7 mg, 3.3 mmol) with chloro-oxime **17a** according to General Procedure A. A yellow solid was obtained (717.4 mg, 66%). M.p.: 125 °C; ^1^H NMR (300 MHz, DMSO-*d*_*6*_) δ 5.88 (s, 2H), 6.98 (*s*, 1H), 7.06–7.09 (m, 4H), 7.21 (t, 1H, *J* 6 Hz), 7.29 (*d*, 1H, *J* 0.88 Hz), 7.41–7.46 (m, 2H), 7.84–7.88 (m, 3H). ^13^C NMR (75 MHz, DMSO-*d*_*6*_) δ 45.2, 101.5, 118.9, 119.8, 123.4, 124.6, 128.6, 128.7, 129.0, 130.6, 144.8, 156.1, 159.1, 162.0, 167.7. HRMS (ESI+) *m*/*z* 363.1082 [M + H]^+^ (calcd. *m*/*z* 363.1093 for C_19_H_15_N_4_O_4_^+^).

##### 3-(4-(4-Fluorophenoxy)­phenyl)-5-((2-nitro-1H-imidazol-1-yl)­methyl)­isoxazole (**6b**)

Reaction of propargyl-2-nitroimidazol **12** (498.7 mg, 3.3 mmol) with chloro-oxime **17b** according to General Procedure A. A yellow solid was obtained (924.2 mg, 81%). M.p.: 85 °C; ^1^H NMR (300 MHz, DMSO-*d*_*6*_) δ 5.88 (s, 2H), 6.98 (*s*, 1H), 7.05 (d, 2H *J* 8.7 Hz), 7.14–7.17 (m, 2H), 7.23–7.29 (m, 3H), 7.84–7.87 (m, 3H). ^13^C NMR (75 MHz, DMSO-*d*_*6*_) δ 44.7, 101.1, 116.7 (d, *J*_C–F_ 23 Hz), 117.9, 121.5 (d, *J*_C–F_ 8.5 Hz), 122.8, 128.1, 128.2, 128.6, 144.3, 151.6 (d, *J*_C–F_ 2.3 Hz), 158.6 (d, *J*_C–F_ 238 Hz), 159.0, 161.5, 167.2. HRMS (ESI+) *m*/*z* 381.0990 [M + H]^+^ (calcd *m*/*z* 381.0999 for C_19_H_14_FN_4_O_4_^+^).

##### 3-(4-(4-Chlorophenoxy)­phenyl)-5-((2-nitro-1H-imidazol-1-yl)­methyl)­isoxazole (**6c**)

Reaction of propargyl-2-nitroimidazol **12** (498.7 mg, 3.3 mmol) with chloro-oxime **17c** according to General Procedure A. A yellow solid was obtained (773.7 mg, 65%). M.p.: 109 °C; ^1^H NMR (300 MHz, DMSO-*d*_*6*_) δ 5.88 (s, 2H), 7.00 (*s*, 1H), 7.10–7.13 (m, 4H), 7.29 (d, 1H, *J* 0.87 Hz), 7.47 (*d*, 2H, *J* 8.9 Hz), 7.84–7.89 (m, 3H). ^13^C NMR (75 MHz, DMSO-*d*_*6*_) δ 45.2, 101.5, 119.2, 121.4, 123.8, 128.4, 128.6, 128.7, 129.1, 130.5 144.9, 155.2, 158.6, 161.9, 167.8. HRMS (ESI+) *m*/*z* 397.0705 [M + H]^+^ (calcd *m*/*z* 397.0704 for C_19_H_14_ClN_4_O_4_^+^).

##### 3-(4-(3,4-Dichlorophenoxy)­phenyl)-5-((2-nitro-1H-imidazol-1-yl)­methyl)­isoxazole (**6d**)

Reaction of propargyl-2-nitroimidazol **12** (498.7 mg, 3.3 mmol) with chloro-oxime **17d** according to General Procedure A. A yellow solid was obtained (802.1 mg, 62%). M.p.: 134 °C; ^1^H NMR (300 MHz, DM*S*O-*d*_*6*_) δ 5.8 (s, 2H), 7.01 (s, 1H), 7.08 (dd, 1H, *J* 2.8 and 8.8 Hz), 7.17 (d, 2H, *J* 8.6 Hz), 7.29 (d, 1H, *J* 0.85 Hz), 7.39 (d, 1H, *J* 2.8 Hz), 7.66 (d, 1H, *J* 8.8 Hz), 7.84 (s, 1H), 7.90 (d, 2H, *J* 8.6 Hz). ^13^C NMR (75 MHz, DM*S*O-*d*_6_) δ 45.2, 101.6, 119.7, 119.8, 121.4, 124.4, 126.5, 128.6, 128.7, 129.2, 132.1, 132.6, 144.8, 156.0, 158.0, 161.9, 167.8. HRMS (ESI+) *m*/*z* 431.0300 [M + H]^+^ (calcd *m*/*z* 431.0314 for C_19_H_13_Cl_2_N_4_O_4_^+^).

##### 5-((2-Nitro-1H-imidazol-1-yl)­methyl)-3-(4-(4-(trifluoromethoxy)­phenoxy)­phenyl)­isoxazole (**6e**)

Reaction of propargyl-2-nitroimidazol **12** (498.7 mg, 3.3 mmol) with chloro-oxime **17e** according to General Procedure A. A yellow solid was obtained (388.1 mg, 29%). M.p.: 92 °C; ^1^H NMR (500 MHz, DMSO-*d*_*6*_) δ 5.89 (s, 2H), 7.01 (s, 1H), 7.15 (d, 2H, *J* 6.8 Hz), 7.20 (d, 2H, *J* 6.8 Hz), 7.30 (d, 1H, *J* 1.0 Hz), 7.43 (d, 2H, *J* 8.9 Hz), 7.85 (d, 1H, *J* 1.0 Hz), 7.91 (d, 2H, *J* 8.9 Hz). ^13^C NMR (100 MHz, DMSO-*d*_*6*_) δ 45.2, 101.6, 119.4, 120.5 (q, *J* 203 Hz), 121.0, 123.5, 124.0, 128.6, 128.7, 129.2, 144.6 (q, *J* 1.2 Hz), 144.8, 155.2, 158.5, 161.9, 167.8. HRMS (ESI+) *m*/*z* 447.0914 [M + H]^+^ (calcd *m*/*z* 447.0916 for C_20_H_15_F_3_N_4_O_5_^+^).

##### 5-((2-Nitro-1H-imidazol-1-yl)­methyl)-3-(4-(P-tolyloxy)­phenyl)­isoxazole (**6f**)

Reaction of propargyl-2-nitroimidazol **12** (498.7 mg, 3.3 mmol) with chloro-oxime **17f** according to General Procedure A. A yellow solid was obtained (338.7 mg, 30%). M.p.: 102 °C; ^1^H NMR (300 MHz, DMSO-*d*_*6*_) δ 2.30 (s, 3H), 5.88 (s, 2H), 6.97–7.04 (m, 5H), 7.23 (d, 2H, *J* 8.2 Hz), 7.29 (d, 1H, *J* 0.8 Hz), 7.83–7.85 (m, 3H). ^13^C NMR (75 MHz, DMSO-*d*_*6*_) δ 20.7, 45.2, 101.5, 118.3, 120.0, 122.9, 128.6, 128.7, 129.0, 131.0, 133.9, 144.7, 153.6, 159.6, 162.0, 167.6. HRMS (ESI+) *m*/*z* 377.1250 [M + H]^+^ (calcd *m*/*z* 377.1250 for C_20_H_17_N_4_O_4_^+^).

##### 3-(4-(4-Methoxyphenoxy)­phenyl)-5-((2-nitro-1H-imidazol-1-yl)­methyl)­isoxazole (**6g**)

Reaction of propargyl-2-nitroimidazol **12** (498.7 mg, 3.3 mmol) with chloro-oxime **17b** according to General Procedure A. A pale yellow solid was obtained (906.3 mg, 77%). M.p.: 104 °C; ^1^H NMR (300 MHz, DMSO-*d*_*6*_) δ 3.76 (s, 3H), 5.88 (s, 2H), 6.96–7.00 (m, 5H), 7.05–7.08 (m, 2H), 7.29 (d, 1H, *J* 0.8 Hz), 7.81–7.84 (m, 3H). ^13^C NMR (75 MHz, DMSO-*d*_*6*_) δ 45.1, 55.8, 101.5, 115.6, 117.7, 121.7, 122.6, 128.6, 128.7, 128.9, 144.7, 148.8, 156.5, 160.3, 162.0, 167.6. HRMS (ESI+) *m*/*z* 393.1198 [M + H]^+^ (calcd for *m*/*z* 393.1199 for C_20_H_17_N_4_O_5_).

##### 3-(3-Fluoro-4-phenoxyphenyl)-5-((2-nitro-1H-imidazol-1-yl)­methyl)­isoxazole (**6h**)

Reaction of propargyl-2-nitroimidazol **12** (498.7 mg, 3.3 mmol) with chloro-oxime **17h** according to General Procedure A. A pale yellow solid was obtained (570.5 mg, 50%). M.p.: 152 °C; ^1^H NMR (500 MHz, DMSO-*d*_*6*_) 5.91 (s, 2H), 7.05–7.07 (m, 3H), 7.16–7.23 (m, 2H), 7.31 (d, 1H, *J* 0.95 Hz), 7.40–7.43 (m, 2H), 7.72–7.74 (m, 1H), 7.86 (d, 1H, *J* 0.95 Hz), 7.91 (dd, 1H, *J* 2 and 11.7 Hz). ^13^C NMR (100 MHz, DMSO-*d*_*6*_) δ 45.3, 101.7, 116.1 (d, J 16 Hz), 118.1, 122.2, 124.2 (d, J 2.6 Hz), 124.4, 125.4 (d, *J*_C–F_ 5.7 Hz), 128.6, 128.7, 130.6, 144.8, 145.3 (d, *J*_C–F_ 8.8 Hz), 153.8 (d, *J*_C–F_ 196 Hz), 156.6, 161.3 (d, *J*_C–F_ 1.4 Hz), 168.2. HRMS (ESI+) *m*/*z* 381.0997 [M + H]^+^ (calcd *m*/*z* 381.0999 for C_19_H_14_FN_4_O_4_^+^).

##### 3-(3-Fluoro-4-(4-fluorophenoxy)­phenyl)-5-((2-nitro-1H-imidazol-1-yl)­methyl)­isoxazole (**6i**)

Reaction of propargyl-2-nitroimidazol **12** (498.7 mg, 3.3 mmol) with chloro-oxime **17i** according to General Procedure A. A white solid was obtained (728.9 mg, 61%). M.p.: 111 °C; ^1^H NMR (500 MHz, DMSO-*d*_*6*_) δ 5.90 (s, 2H), 7.06 (s, 1H), 7.12–7.19 (m, 3H), 7.23–7.27 (m, 2H), 7.31 (d, 1H, *J* 1.0 Hz), 7.70–7.72 (m, 1H), 7.85 (d, 1H, *J* 1 Hz), 7.90 (dd, 1H, *J* 1.9 and 11.8 Hz). ^13^C NMR (100 MHz, DMSO-*d*_*6*_) δ 45.2, 101.7, 116.1 (d, *J*_C–F_ 15.9 Hz), 117.2 (d, *J*_C–F_ 18.8 Hz), 120.3 (d, *J*_C–F_ 6.6 Hz), 121.5, 124.2 (d, *J*_C–F_ 2.7 Hz), 125.2 (d, *J*_C–F_ 6.1 Hz), 128.6, 128.7, 144.8, 145.8 (d, *J*_C–F_ 8.8 Hz), 152.5 (d, *J*_C–F_ 1.1 Hz), 153.5 (d, *J*_C–F_ 196 Hz), 158.9 (d, *J*_C–F_ 161 Hz), 161.3 (d, *J*_C–F_ 1.3 Hz), 168.2. HRMS (ESI+) *m*/*z* 399.0903 [M + H]^+^ (calcd *m*/*z* 399.0905 for C_19_H_13_F_2_N_4_O_4_^+^).

##### 3-(4-(4-Chlorophenoxy)-3-fluorophenyl)-5-((2-nitro-1H-imidazol-1-yl)­methyl)­isoxazole (**6j**)

Reaction of propargyl-2-nitroimidazol **12** (498.7 mg, 3.3 mmol) with chloro-oxime **17j** according to General Procedure A. A pale yellow solid was obtained (696.8 mg, 56%). M.p.: 136.5 °C; ^1^H NMR (500 MHz, DMSO-*d*_*6*_) δ 5.91 (s, 2H), 7.08–7.10 (m, 3H), 7.25–7.29 (m, 1H), 7.31 (d, 1H, *J* 1.05 Hz), 7.45 (d, 2H, *J* 8.9 Hz), 7.73–7.75 (m, 1H), 7.86 (s, 1H), 7.92 (dd, 1H, *J* 1.9 and 11 Hz). ^13^C NMR (100 MHz, DMSO-*d*_*6*_) δ 45.3, 101.7, 116.2 (d, *J*_C–F_ 16 Hz), 119.7, 122.5, 124.3 (d, *J*_C–F_ 2.5 Hz), 125.8 (d, *J*_C–F_ 6 Hz), 128.2, 128.6, 128.7, 130.4, 144.8, 144.9, 153.8 (d, *J*_C–F_ 197 Hz), 155.5, 161.3 (d, *J*_C–F_ 1.4 Hz), 168.3. HRMS (ESI+) *m*/*z* 415.0608 [M + H]^+^ (calcd *m*/*z* 415.0609 for C_19_H_13_ClFN_4_O_4_^+^).

##### 3-(4-(3,4-Dichlorophenoxy)-3-fluorophenyl)-5-((2-nitro-1H-imidazol-1-yl)­methyl)­isoxazole (**6k**)

Reaction of propargyl-2-nitroimidazol **12** (498.7 mg, 3.3 mmol) with chloro-oxime **17j** according to General Procedure A. A pale yellow solid was obtained (929.9 mg, 69%). M.p.: 127 °C; ^1^H NMR (500 MHz, DMSO-*d*_*6*_) δ 5.9 (s, 2H), 8.06–7.09 (m, 2H), 7.31 (d, 1H, *J* 1.1 Hz), 7.35 (t, 1H, *J* 8.5 Hz), 7.40 (d, 1H, *J* 2.8 Hz), 7.64 (d, 1H, *J* 8.9 Hz), 7.75–7.77 (m, 1H), 7.86 (d, 1H, *J* 1.1 Hz), 7.94 (dd, 1H, *J* 2 and 11 Hz). ^13^C NMR (100 MHz, DMSO-*d*_*6*_) δ 45.3, 101.7, 116.3 (d, *J*_C–F_ 16 Hz), 118.2, 120.9, 122.9, 124.4 (d, *J*_C–F_ 2.4 Hz), 126.3, 126.4, 128.6, 128.7, 132.1, 132.6, 144.2 (d, *J*_C–F_ 9 Hz), 144.8, 153.8 (d, *J*_C–F_ 197 Hz), 156.1, 161.3 (d, *J*_C–F_ 1.3 Hz), 168.3. HRMS (ESI+) *m*/*z* 449.0215 [M + H]^+^ (calcd *m*/*z* 449.0220 for C_19_H_12_Cl_2_FN_4_O_4_^+^).

##### 3-(3-Fluoro-4-(4-(trifluoromethoxy)­phenoxy)­phenyl)-5-((2-nitro-1H-imidazol-1Yl)­methyl)­isoxazole (**6l**)

Reaction of propargyl-2-nitroimidazol **12** (498.7 mg, 3.3 mmol) with chloro-oxime **17l** according to General Procedure A. A pale yellow solid was obtained (640.8 mg, 46%). M.p.: 91 °C; ^1^H NMR (300 MHz, CDCl_3_) δ 5.77 (s, 2H), 6.61 (s, 1H), 7.00 (d, 2H, *J* 9 Hz), 7.08 (t, 1H, *J* 8 Hz), 7.17–7.29 (m, 5H), 7.48 (d, 1H, *J* 8 Hz), 7.62 (dd, 1H, *J* 1.7 and 11 Hz). ^13^C NMR (75 MHz, CDCl_3_) δ 44.4, 102.3, 115.7 (d, *J*_C–F_ 20 Hz), 188.8, 120.4 (q, *J*_C–F_ 250 Hz), 121.6 (d, *J*_C–F_ 2.2 Hz), 122.7, 123.4 (d, *J*_C–F_ 3.7 Hz), 125.0 (d, *J*_C–F_ 7.1 Hz), 126.1, 129.0, 144.2, 144.9 (q, *J*_C–F_ 1.8 Hz), 145.5 (d, *J*_C–F_ 11 Hz), 154.0 (d, *J*_C–F_ 249 Hz), 154.9, 161.4 (d, *J*_C–F_ 2.2 Hz), 165.3. HRMS (ESI+) *m*/*z* 465.0824 [M + H]^+^ (calcd *m*/*z* 465.0822 for C_20_H_13_F_4_N_4_O_5_^+^).

##### 3-(3-Fluoro-4-(P-tolyloxy)­phenyl)-5-((2-nitro-1H-imidazol-1-yl)­methyl)­isoxazole (**6m**)

Reaction of propargyl-2-nitroimidazol **12** (498.7 mg, 3.3 mmol) with aldoxime **16m** according to General Procedure B. A white solid was obtained (425.9 mg, 36%). M.p.: 111 °C; ^1^H NMR (500 MHz, DMSO-*d*_*6*_) δ 2.29 (s, 3H), 5.90 (s, 2H), 6.96 (d, 2H, *J* 8.5 Hz), 7.06 (s, 1H), 7.12 (t, 1H, *J* 8.5 Hz), 7.02–7.22 (m, 2H), 7.31 (d, 1H, *J* 1 Hz), 7.68–7.70 (m, 1H), 7.85–7.86 (m, 1H), 7.88 (dd, 1 H, *J* 1.9 and 11 Hz). ^13^C NMR (100 MHz, DMSO-*d*_*6*_) δ 20.7, 45.3, 101.7, 116.0 (d, *J*_C–F_ 15.9 Hz), 118.4, 121.4, 124.1 (d, *J*_C–F_2.6 Hz), 124.8 (d, *J*_C–F_ 6.0 Hz), 128.6, 128.7, 130.9, 133.7, 144.8, 146.0 (d, *J*_C–F_ 8.8 Hz), 153.6 (d, *J*_C–F_ 196 Hz), 154.1, 161.3 (d, *J*_C–F_ 1.3 Hz), 168.2. HRMS (ESI+) *m*/*z* 395.1169 [M + H]^+^ (calcd *m*/*z* 395.1156 for C_20_H_16_FN_4_O_4_^+^).

##### 3-(3-Fluoro-4-(4-methoxyphenoxy)­phenyl)-5-((2-nitro-1H-imidazol-1-yl)­methyl)­isoxazole (**6n**)

Reaction of propargyl-2-nitroimidazol **12** (498.7 mg, 3.3 mmol) with chloro-oxime **17n** according to General Procedure A. A pale yellow solid was obtained (664.8 mg, 54%). M.p.: 113.5 °C; ^1^H NMR (500 MHz, DMSO-*d*_*6*_) δ 3.75 (s, 3H), 5.90 (s, 2H), 6.98 (d, 2H, *J* 9.1 Hz), 7.01–7.08 (m, 4H), 7.31 (d, 1H, *J* 1.0 Hz), 7.66–7.68 (m, 1H), 7.85–7.88 (m, 2H). ^13^C NMR (100 MHz, DMSO-*d*_*6*_) 45.3, 55.9, 101.7, 115.6, 115.9 (d, *J*_C–F_ 15.9 Hz), 120.2, 120.4, 124.0 (d, *J*_C–F_ 2.4 Hz), 124.2 (d, *J*_C–F_ 6.0 Hz), 128.6, 128.7, 144.8, 147.0 (d, *J*_C–F_ 8.7 Hz), 149.3, 153.1 (d, *J*_C–F_ 196 Hz), 156.4, 161.4 (d, *J*_C–F_ 1.3 Hz), 168.1. HRMS (ESI+) *m*/*z* 411.1120 [M + H]^+^ (calcd *m*/*z* 411.1105 for C_20_H_16_FN_4_O_5_^+^).

##### 5-((2-Nitro-1H-imidazol-1-yl)­methyl)-3-(4-(phenylthio)­phenyl)­isoxazole (**6o**)

Reaction of propargyl-2-nitroimidazol **12** (498.7 mg, 3.3 mmol) with chloro-oxime **17o** according to General Procedure A. A yellow solid was obtained (506.8 mg, 45%). M.p.: 147.5 °C; ^1^H NMR (500 MHz, DMSO-*d*_*6*_) δ 5.89 (s, 2H), 7.10 (s, 1H), 7.30 (d, 1H, *J* 1.0 Hz), 7.34 (d, 2H, *J* 8.5 Hz), 7.38–7.41 (m, 1H), 7.43–7.44 (m, 4H), 7.83 (d, 2H, *J* 8.5 Hz), 7.85 (d, 1H, *J* 1.0 Hz). ^13^C NMR (100 MHz, DMSO-*d*_*6*_) δ 45.2, 101.6, 126.8, 128.0, 128.6, 128.7, 128.8, 130.0, 130.3, 132.6, 133.4, 139.2, 144.8, 162.0, 167.9. HRMS (ESI+) *m*/*z* 379.0874 [M + H]^+^ (calcd *m*/*z* 379.0865 for C_19_H_15_N_4_O_3_S^+^).

##### 3-(4-((4-Chlorophenyl)­thio)­phenyl)-5-((2-nitro-1H-imidazol-1-yl)­methyl)­isoxazole (**6p**)

Reaction of propargyl-2-nitroimidazol **12** (498.7 mg, 3.3 mmol) with chloro-oxime **17p** according to General Procedure A. A yellow solid was obtained (792.6 mg, 64%). M.p.: 135 °C; ^1^H NMR (300 MHz, DMSO-*d*_*6*_) δ 5.89 (s, 2H), 7.02 (*s*, 1H), 7.29 (*s*, 1H), 7.37–7.50 (*m*, 6H), 7.83–7.86 (*m*, 3H). ^13^C NMR (75 MHz, DMSO-*d*_*6*_) δ 45.2, 101.6, 127.2, 128.2, 128.6, 128.7, 130.2, 130.7, 132.9, 133.3, 133.8, 138.2, 144.8, 161.9, 167.9. HRMS (ESI+) *m*/*z* 413.0470 [M + H]^+^ (calcd *m*/*z* 413.0475 for C_19_H_14_ClN_4_O_3_S).

##### 5-((2-Nitro-1H-imidazol-1-yl)­methyl)-3-(4-(P-tolylthio)­phenyl)­isoxazole (**6q**)

Reaction of propargyl-2-nitroimidazol **12** (498.7 mg, 3.3 mmol) with aldoxime **16q** according to General Procedure B. A white solid was obtained (400.3 mg, 34%). M.p.: 134 °C; ^1^H NMR (300 MHz, CDCl_3_) δ 2.32 (s, 3H), 5.75 (s, 2H), 6.62 (s, 1H), 7.18 (s, 1H), 7.23 (d, 2H, *J* 8.0 Hz), 7.27 (s, 1H), 7.50 (d, 2H, *J* 8 Hz), 7.67 (d, 2H, *J* 8.3 Hz), 7.81 (d, 2H, *J* 8.3 Hz). ^13^C NMR (75 MHz, CDCl_3_) δ 21.3, 44.4, 102.4, 125.0, 125.1, 126.2, 127.6, 129.0, 130.2, 130.4, 142.0, 142.1, 144.2, 148.2, 161.9, 165.4. HRMS (ESI+) *m*/*z* 409.0983 [M+H_2_O–H]^+^ (calcd. *m*/*z* 409.0976 for C_20_H_17_N_4_O_4_S).

### Biological Methods

#### Antitrypanosomal Assay

Initially, 5 × 10^3^ LLC-MK2 cells/well were infected with trypomastigotes in 96-well plates at a parasite/cell ratio of 10:1. After 48 h, plates were washed twice with PBS before adding compounds in a serial dilution range of 100 μM to 0.78 μM in fresh RPMI 1640 medium (Sigma-Aldrich). As a control, infected untreated cells (100% infection control) were employed. Following 72 h of incubation, amastigote viability tests were performed using 50 μL of PBS containing 2% Triton X-100 and 200 μM CPRG.^43^ Tulahuen lacZ strain expressing galactosidase enzyme were used, which catalyzes the yellow reagent of CPRG into a red chromophore that can be easily detected by absorbance at 570 nm after 4 h at 37 °C (microplate reader – Synergy H1).^44^ The percentage of amastigotes inhibition (IC_50_) was calculated as 100-(absorbance of treated infected cells)/(absorbance mean of untreated infected cells) × 100.

#### Cytotoxicity Assay^45^

To assess chemical cytotoxicity, MTT (3-(4,5-Dimethyl-2-thiazolyl)-2,5-diphenyl-2H-tetrazolium bromide, Sigma-Aldrich)-based kit reagents were employed. LLC-MK2 cells were incubated in 96-well plates for 48 h at a concentration of 5 × 10^3^ cells/well. Cells were then washed twice with PBS (Sigma-Aldrich) before being incubated with compounds in a serial dilution from 200 μM to 1.56 μM in fresh RPMI 1640 medium (Sigma-Aldrich). After 72 h, 50 μL of MTT solution (2.0 mg/mL) was added to each well, and the plates were incubated for another 4 h at 37 °C. The resultant formazan crystals were dissolved in DMSO (50 μ L/well) and measured at 570 nm 30 min later in a Synergy H1 microplate reader. Cytotoxic concentration of 50% (CC_50_) was calculated based on a dose–response curve excluding DMSO cytotoxicity (>1%) of the untreated control.

## Supplementary Material



## Notes

Synthetic procedures and analytical characterization of precursors as well as copies of NMR spectra of 2-nitroimidazole-3,5-disubstituted isoxazole derivatives with diaryl ether and thioether substituents (PDF)
