# Supplementary material for: Synthesis and Antitrypanosomal Activity of Novel 2‑Nitroimidazole-3,5-Disubstituted Isoxazole Derivatives with Diaryl Ether and Thioether Substituents
Source: ACS Omega. 2025 Nov 15;10(46):56378–87. doi: 10.1021/acsomega.5c08312 (PMC12658631; doi:10.1021/acsomega.5c08312)
Supplement: Supplementary file 1 [file ao5c08312_si_001.pdf]

Supporting Information

Synthesis and Antitrypanosomal Activity of Novel 2-Nitroimidazole-3,5-disubstituted Isoxazole Derivatives with Diaryl Ether and Thioether Substituents

Larissa B. B. Santos[a], Diego B. Carvalho[a], Cristiane Y. K. Shiguemoto[a], Omar D. Lacerda[a], Talícia S. Silva [b], Gisele B. Portapilla[b], Saulo E. Silva-Filho[c], Palimécio G. Guerrero Jr.[d], Sérgio de Albuquerque[b]*, Adriano C. M. Baroni[a]*

[a] Laboratório de Síntese e Química Medicial (LASQUIM), Faculdade de Ciências Farmacêuticas, Alimentos e Nutrição (FACFAN), Universidade Federal de Mato Grosso do Sul (UFMS), Avenida Costa e Silva, s.n., Bairro Universitário, 79070-900, Campo Grande, Mato Grosso do Sul, Brazil.

[b] Departamento de Análise Clínica, Toxicológicas e Bromatológicas, Faculdade de Ciências Farmacêuticas de Ribeirão Preto, Universidade de São Paulo (USP), Avenida do Café, s.n., Monte Alegre, 14040-903, Ribeirão Preto, São Paulo, Brazil

[c] Faculdade de Ciências Farmacêuticas, Alimentos e Nutrição (FACFAN), Universidade Federal de Mato Grosso do Sul (UFMS), Avenida Costa e Silva, s.n., Bairro Universitário, 79070-900, Campo Grande, Mato Grosso do Sul, Brazil

[d] Universidade Tecnológica Federal do Paraná, Química, Rua Deputado Heitor Alencar Furtado, 4900, Cidade Industrial, 81280-340, Curitiba, Paraná, Brazil

Email: sdalbuqu@fcfrp.usp.br

Email: adriano.baroni@ufms.br

Table of contents

Synthesis of 2-aminoimidazole **9** S3
Synthesis of 2-nitroimidazole **10** S3
Synthesis of propargyl-2-nitroimidazole **12** S4
Synthesis of diaryl ether aldehydes **15a-h, k-n, q** S4
Synthesis of diaryl ether aldoximes **16a-h, k-n, q** S6
Synthesis of diaryl ether chloro-oximes **17a-h, k-l, n** S8
Synthesis of diaryl ether and thioether benzonitriles **19i-j, o-p** S10
Synthesis of diaryl ether and thioether amidoximes **20i-j, o-p** S11
Synthesis of diaryl ether and thioether chloro-oximes **17i-j, o-p** S12
References S12
^{1}H NMR and ^{13}C NMR spectra of compounds S13

Synthesis of 2-aminoimidazole 9

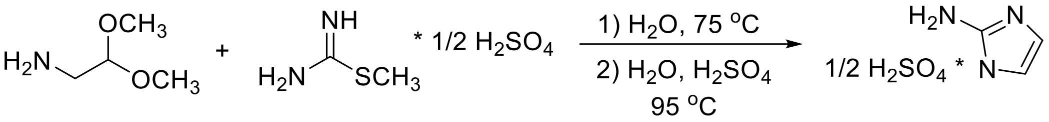

S-methylisothiouronium hemisulfate (142 g, 1.0 eq., 1.0 mol) was added at room temperature to a solution of 2-aminoacetaldehyde dimethyl acetal (105 g, 1.0 eq., 1.0 mol) in 230 mL of water. The mixture was heated slowly to reach 75 °C within 2 h, and kept at this temperature for 1h. After that, the solution was evaporated in vacuum at 60 °C, then 80 °C to remove the most part of solvent and gas. The viscous residue was dissolved in 1 L of water and sulfuric acid was added until pH 0.4. The solution was slowly heated to 95 °C within 45 min. and stirred for 30 min. at this temperature. The solvent was removed under reduced pressure at 80 °C until the formation of viscous residue. Then, this residue was added to 400 mL of cold ethanol and vigorous stirred. The resulting white precipitate was filtered, washed with cold ethanol and drying under vacuum to afford the compound. The product was obtained as a white solid in 92% yield (243.15 g). The obtained characterizing data are consistent with literature information.[1]

Synthesis of 2-nitroimidazole 10

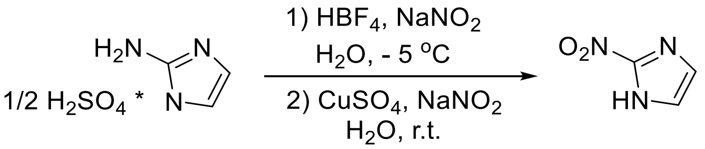

To a solution of 2-aminoimidazolium sulfate **9** (18.79 g, 1.0 eq., 72 mmol) and fluoroboric acid (63.0 mL, 6.68 eq., 481 mmol) in 120 mL of water was added dropwise 80 mL of an aqueous solution of sodium nitrite (49.67 g, 10.0 eq., 720 mmol) at – 5 °C and maintained stirring for 30 min. The reaction mixture was added to a solution of $CuSO_4.5H_2O$ (239.95 g, 13.3 eq., 961 mmol) in 800 mL of H_2O at room temperature. Sodium nitrite (49.67 g, 10.0 eq., 720 mmol) was added to this mixture and allowed to stir for 24h at room temperature. After this period, NaOH was added until pH 13 – 14 and the resulting precipitate was filtered and discarded. The pH of the solution was adjust to approximately 2.0 with concentrated HCl leading to precipitation of the 2-nitroimidazole. Then, this solid was filtered and allowed to dry at room temperature. The product was obtained as a yellow solid 86% yield (7.0 g). The obtained characterizing data are consistent with literature information.[1]

Synthesis of propargyl-2-nitroimidazole 12

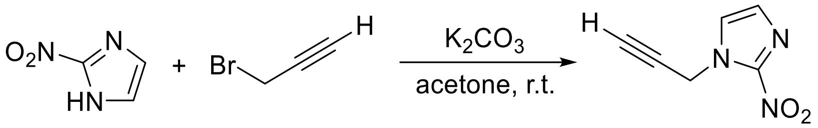

A suspension of 2-nitroimidazole **10** (0.9 g, 1.0 eq., 8.0 mmol) and K_2CO_3 (1.54 g, 1.4 eq., 11.2 mmol) in 20 mL of dry acetone was stirred for 5 min. at room temperature. Propargyl bromide **11** (80% w/v in toluene, 1.07 mL, 1.2 eq., 9.6 mmol) was added and the mixture was allowed to stir for 24h. The solvent was evaporated under reduced pressure and the residue was solubilized in water and extracted with EtOAc (3 x 30 mL). The combined organic layers were dried over anhydrous $MgSO_4$ and solvent removed. The product was purified by flash column chromatography using hexane:EtOAc (8:2) as eluent. The product was obtained as a pale yellow oil in 85% yield (1.03 g). The obtained characterizing data are consistent with literature information.[1]

Synthesis of diaryl ether aldehydes 15a-h, k-n, q

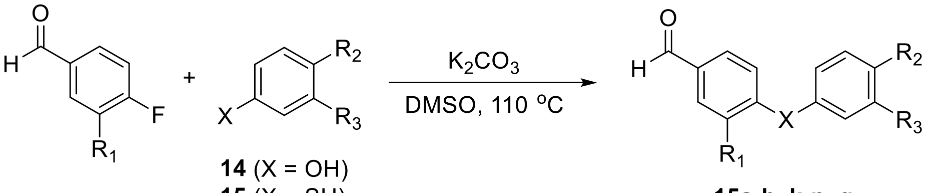

The suitable phenol **14**/thiol **15** compounds with various substituents (1.0 eq., 20 mmol), fluorobenzaldehyde derivative **13** (1.0 eq., 20 mmol), potassium carbonate (8.29 g, 3.0 eq., 60 mmol), and dimethylsulfoxide (28 mL) were poured into a reaction flask equipped with magnetic stirring and a condenser. The mixture was heated to 110 °C and the conversion of the compounds to the desired product was monitored by TLC. Upon completion, the resulting mixture was dissolved in ethyl acetate (150 mL) and washed with distilled water (100 mL). The aqueous phase was then extracted with ethyl acetate (3 x 50 mL). The organic phases were combined, dried over anhydrous magnesium sulfate, filtered and concentrated under reduced pressure.[2]

The obtained characterizing data are consistent with literature information for 4-phenoxybenzaldehyde (**15a**)[3], 4-(4-fluorophenoxy)benzaldehyde (**15b**)[3], 4-(4-chlorophenoxy)benzaldehyde (**15c**)[3], 4-(3,4-dichlorophenoxy)benzaldehyde (**15d**)[4], 4-(4-methylphenoxy)benzaldehyde (**15f**)[3], 4-(4-methoxyphenoxy)benzaldehyde (**15g**)[3], 4-(p-tolylthio)benzaldehyde (**15q**)[5].

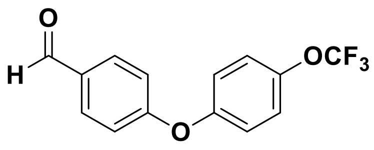

4-(4-(trifluoromethoxy)phenoxy)benzaldehyde (**15e**) was obtained in 97% yield. ^{1}H NMR (300 MHz, $CDCl_3$) δ 7.02-7.08 (m, 4H), 7.19 (d, 2H, *J* 7.8 Hz), 7.83 (d, 2H, *J* 7.8 Hz), 9.88 (s, 1H,).

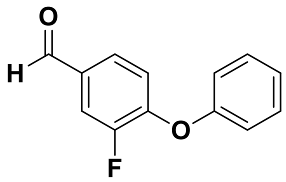

3-fluoro-4-phenoxybenzaldehyde (**15h**) was obtained in 55% yield. ^{1}H NMR (300 MHz, $CDCl_3$) δ 6.95-7.07 (m, 3H), 7.17-7.23 (m, 1H), 7.36-7.41 (m, 2H), 7.55-7.58 (m, 1H), 7.66-7.70 (m, 1H), 9.88 (s, 1H)

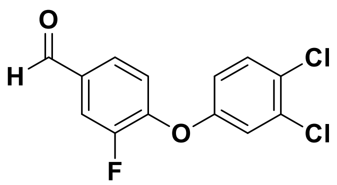

4-(3,4-dichlorophenoxy)-3-fluorobenzaldehyde (**15k**) was obtained in 56% yield. ^{1}H NMR (300 MHz, $CDCl_3$) δ 6.9 (dd, 1H, J 2.7 and 8.7 Hz), 7.08-7.14 (m, 2H), 7.43 (d, 1H, *J* 8.8 Hz), 7.64 (d, 1H, J 8.4 Hz), 7.70 (dd, 1H, *J* 1.6 and 10.3 Hz), 9.91 (s, 1H).

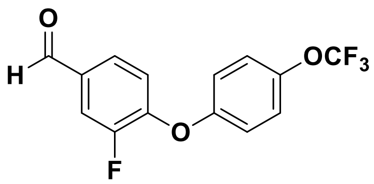

3-fluoro-4-(4-(trifluoromethoxy)phenoxy)benzaldehyde (**15l**) was obtained in 93% yield. ^{1}H NMR (300 MHz, $CDCl_3$) δ 7.03-7.08 (m, 3H), 7.21-7.24 (m, 2H), 7.61 (d, 1H, *J* 8.4 Hz), 7.70 (dd, 1H, *J* 1.7 Hz and 10.3 Hz), 9.90 (s, 1H).

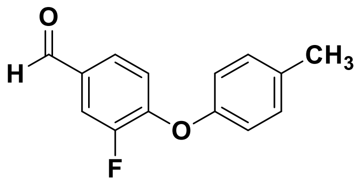

3-fluoro-4-(*p*-tolyloxy)benzaldehyde (**15m**) was obtained in 48% yield. ^{1}H NMR (300 MHz, $CDCl_3$) δ 2.34 (s, 3H), 6.92-6.97 (m, 3H), 7.17-7.20 (m, 2H), 7.54 (d, 1H, *J* 8.3 Hz), 7.67 (dd, 1H, *J* 1.8 and 10.5 Hz), 9.86 (s, 1H).

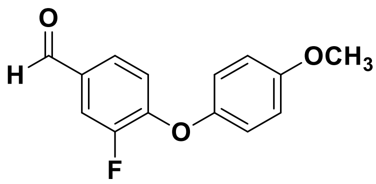

3-fluoro-4-(4-methoxyphenoxy)benzaldehyde (**15n**) was obtained in 64% yield. ^{1}H NMR (300 MHz, $CDCl_3$) δ 3.77 (s, 3H), 6.94-6.99 (m, 3H), 7.18-7.21 (m, 2H), 7.53 (d, 1H, *J* 8.6 Hz), 7.69 (dd, 1H, *J* 1.7 and 11.1 Hz), 9.89 (s, 1H).

Synthesis of diaryl ether aldoximes 16a-h, k-n, q

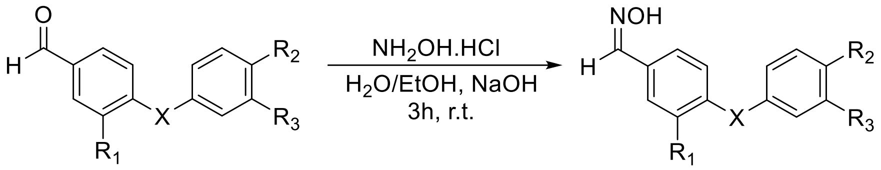

15a-h, k-n, q **16a-h, k-n, q**

The corresponding aldehyde **15a-h, k-n, q** (15 mmol) and hydroxylamine hydrochloride (1.25 g, 1.2 eq., 18 mmol) were added to a water/ethanol mixture (18 mL:18 mL) until completely dissolved. Then, a solution of NaOH (0.72 g, 1.2 eq., 18 mmol) was added dropwise and the reaction mixture was stirred for approximately 3h at room temperature. After completion of reaction, the mixture was extracted with ethyl acetate (3 x 50 mL). The ethyl acetate phases were combined, dried over anhydrous $MgSO_4$, filtered and the solvent were removed under reduced pressure. The aldoximes were used without further purification.[1]

The obtained characterizing data are consistent with literature information for 4-phenoxybenzaldehyde oxime **(16a)**[6].

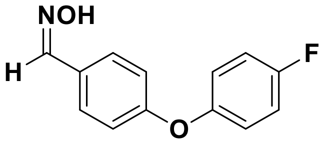

4-(4-fluorophenoxy)benzaldehyde oxime (**16b**) was obtained in 91% yield. ^{1}H NMR (300 MHz, $CDCl_3$): δ 6.90-7.08 (m, 6H), 7.51 (*d*, *J* 8.7 Hz, 2H), 8.09 (s, 1H), 8.15 (s, 1H).

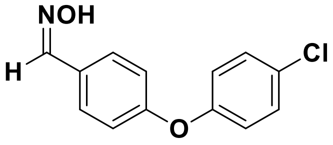

4-(4-chlorophenoxy)benzaldehyde oxime (**16c**) was obtained in 96% yield. ^{1}H NMR (300 MHz, $CDCl_3$): *δ* 6.95 (d, 2H, *J* 8.8 Hz), 6.96 (d, 2H, *J* 8.8 Hz), 7.29 (d, 2H, *J* 8.8 Hz), 7.52 (d, 2H, *J* 8.8 Hz), 8.10 (s, 1H).

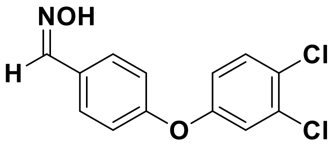

4-(3,4-dichlorophenoxy)benzaldehyde oxime (**16d**) was obtained in 87% yield. ^{1}H NMR (300 MHz, $CDCl_3$): *δ* 6.87 (dd, 1H, *J* 2.7 and 8.8 Hz), 6.98 (d, 2H, *J* 8.8 Hz), 7.11 (d, 1H, *J* 2.7 Hz), 7.38 (d, 2H, *J* 8.8 Hz), 7.55 (d, 2H, *J* 8.6 Hz), 8.10 (s, 1H).

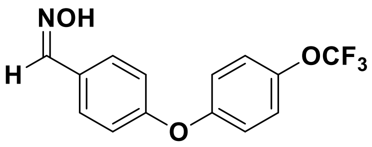

4-(4-(trifluoromethoxy)phenoxy)benzaldehyde oxime (**16e**) was obtained in 97% yield. ^{1}H NMR (300 MHz, $CDCl_3$): *δ* 6.97-7.03 (m, 4H), 7.19 (d, 2H, *J* 8.2 Hz), 7.53 (sl, 2H), 8.14 (s, 1H).

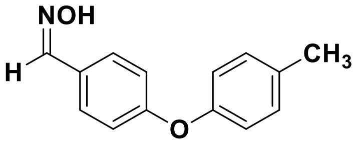

4-(*p*-tolyloxy)benzaldehyde oxime (**16f**) was obtained in 78% yield. ^{1}H NMR (300 MHz, $CDCl_3$) δ 2.33 (s, 3H, CH_3), 6.92 (d, 2H, *J* 8.3 Hz), 6.94 (d, 2H, *J* 8.5 Hz), 7.15 (d, 2H, *J* 8.3 Hz), 7.50 (d, 2H, *J* 8.5 Hz), 8.09 (s, 1H).

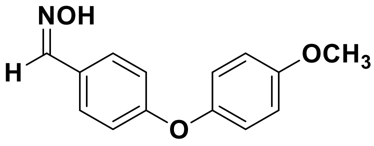

4-(4-methoxyphenoxy)benzaldehyde oxime (**16g**) was obtained in 84% yield. ^{1}H NMR (300 MHz, $CDCl_3$) δ 3.80 (s, 3H, OCH_3), 6.88 (d, 2H, *J* 9.0 Hz), 6.94 (d, 2H, *J* 8.5 Hz), 7.00 (d, 2H, *J* 9.0 Hz), 7.48 (d, 2H, *J* 8.5 Hz), 8.07 (s, 1H).

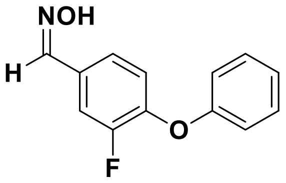

3-fluoro-4-phenoxybenzaldehyde oxime (**16h**) was obtained in 58% yield. ^{1}H NMR (300 MHz, $CDCl_3$) δ 6.95-7.01 (m, 3H), 7.09-7.14 (m, 1H), 7.22-7.24 (m, 1H), 7.30-7.36 (m, 2H), 7.44 (dd, 1H, *J* 1.8 and 11.3 Hz), 8.06 (s, 1H).

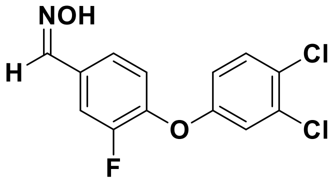

4-(3,4-dichlorophenoxy)-3-fluorobenzaldehyde oxime (**16k**) was obtained in 93% yield. ^{1}H NMR (300 MHz, $CDCl_3$) δ 6.83 (dd, 1H, J 2.7 and 8.8 Hz), 7.02-7.08 (m, 2H), 7.29 (d, 1H, *J* 8.4 Hz), 7.37 (d, 1H, *J* 8.8 Hz), 7.46 (dd, 1H, *J* 1.7 and 11.1 Hz), 8.07 (s, 1H).

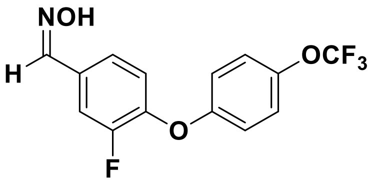

3-fluoro-4-(4-(trifluoromethoxy)phenoxy)benzaldehyde oxime (**16l**) was obtained in 94% yield. ^{1}H NMR (300 MHz, $CDCl_3$) δ 6.96-7.04 (m, 3H), 7.15-7.27 (m, 3H), 7.45 (dd, 1H, *J* 1.6 and 11.2 Hz), 8.07 (s, 1H).

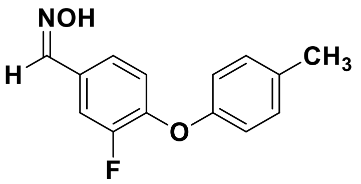

3-fluoro-4-(*p*-tolyloxy)benzaldehyde oxime (**16m**) was obtained in 48% yield. ^{1}H NMR (300 MHz, $CDCl_3$) δ 2.31 (s, 3H), 6.88-6.97 (m, 3H), 7.11-7.20 (m, 3H), 7.42 (dd, 1H, *J* 1.8 and 11.4 Hz), 8.04 (s, 1H).

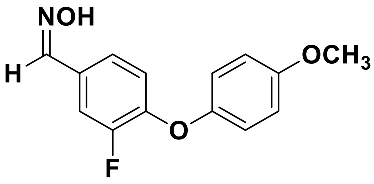

3-fluoro-4-(4-methoxyphenoxy)benzaldehyde oxime (**16n**) was obtained in 87% yield. ^{1}H NMR (300 MHz, $CDCl_3$) δ 3.79 (s, 3H), 6.85-6.88 (m, 3H), 6.95-7.01 (m, 2H), 7.15-7.18 (m, 1H), 7.42 (dd, 1H, *J* 1.6 and 11.4 Hz), 8.03 (s, 1H).

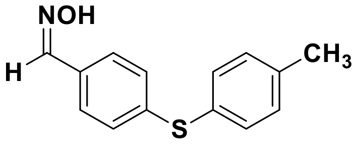

4-(*p*-tolylthio)benzaldehyde oxime (**16q**) was obtained in 60% yield. ^{1}H NMR (300 MHz, $CDCl_3$) δ 2.34 (s, 3H), 7.14-7.17 (m, 4H), 7.33 (d, 2H, *J* 7.9 Hz), 7.42 (d, 2H, J 8.2 Hz), 8.05 (s, 1H).

Synthesis of diaryl ether chloro-oximes 17a-h, k-l, n

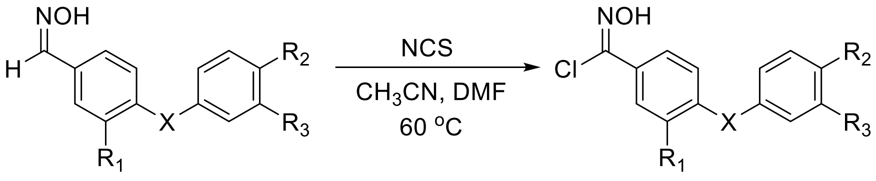

16a-h, k-n, q **17a-h, k-l, n**

Under magnetic stirring and heat, aldoxime **16a-h, k-l, n** (1.0 eq., 10 mmol), acetonitrile (50 mL), and 20% *N*-chlorosuccinimide (0.26 g, 0.2 eq., 2 mmol) were added to a two-neck flask equipped with a nitrogen atmosphere and heating mantle until the system reached 60 ° C. Then, the heating was turned off, and the remaining NCS (1.07 g, 0.8 eq., 8 mmol) dissolved in 10mL of DMF was progressively added to the reaction, so that the temperature did not rise over 60°C. The heat was switched on after the addition of the NCS and the reaction was agitated at 60°C. The conversion to the desired product was monitored by TLC analyses. Upon the completion, the reaction mixture was diluted with ethyl acetate (3 x 30 mL) and rinsed with water (8 x 30 mL). The solvent was removed under low pressure and the organic phase was dried over anhydrous $MgSO_4$. Flash chromatography was used to purify the products, yielding chloro-oximes **17a-h, k-l, n.**[1]

The obtained characterizing data are consistent with literature information for N-hydroxy-4-phenoxybenzimidoyl chloride (**17a**)[7].

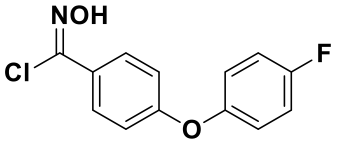

4-(4-fluorophenoxy)-*N*-hydroxybenzimidoyl chloride (**17b**) was obtained in 68% yield. ^{1}H NMR (300 MHz, $CDCl_3$) δ 6.93 (d, 2H, *J* 9.5 Hz), 6.98-7.08 (m, 5H), 7.77 (d, 2H, *J* 9.5 Hz), 8.01 (s, 1H).

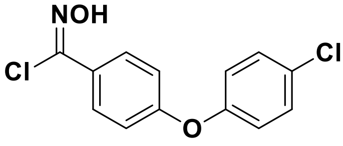

4-(4-chlorophenoxy)-*N*-hydroxybenzimidoyl chloride (**17c**) was obtained in 82% yield. ^{1}H NMR (300 MHz, $CDCl_3$) δ 6.95-6.97 (m, 4H), 7.31 (d, 2H, *J* 8.9 Hz), 7.79 (d, 2H, *J* 8.9 Hz), 8.01 (s, 1H).

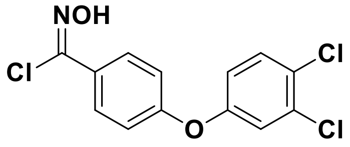

4-(3,4-dichlorophenoxy)-*N*-hydroxybenzimidoyl chloride (**17d**) was obtained in 41% yield. ^{1}H NMR (300 MHz, $CDCl_3$) δ 6.87 (dd, 1H, *J* 2.6 and 8.8 Hz), 6.99 (d, 2H, *J* 8.8 Hz), 7.11 (d, 1H, *J* 2.6 Hz), 7.39 (d, 2H, *J* 8.8 Hz), 7.82 (d, 2H, *J* 8.8 Hz), 8.37 (s, 1H,).

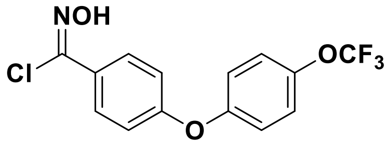

N-hydroxy-4-(4-(trifluoromethoxy)phenoxy)benzimidoyl chloride (**17e**) was obtained in 44% yield. ^{1}H NMR (300 MHz, $CDCl_3$) δ 6.97-7.05 (m, 4H), 7.20 (d, 2H, *J* 8.6 Hz), 7.78-7.79 (d, 2H, *J* 8.8 Hz), 9.06 (sl, 1H).

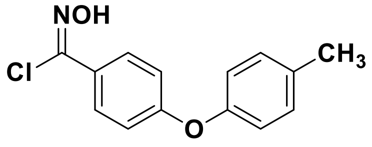

N-hydroxy-4-(*p*-tolyloxy)benzimidoyl chloride (**17f**) was obtained in 33% yield. ^{1}H NMR (300 MHz, $CDCl_3$) δ 2.33 (s, 3H), 6.91-6.95 (m, 4H), 7.15 (d, 2H, *J* 8.4 Hz), 7.75 (d, 2H, *J* 8.8 Hz), 8.05 (s, 1H).

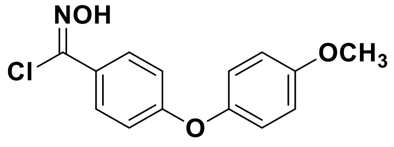

N-hydroxy-4-(4-methoxyphenoxy)benzimidoyl chloride (**17g**) was obtained 78% yield. ^{1}H NMR (300 MHz, $CDCl_3$) δ 3.80 (s, 3H), 6.87-6.92 (m, 4H), 6.97-7.00 (m, 2H), 7.74 (d, 2H, *J* 8.9 Hz), 8.07 (s, 1H).

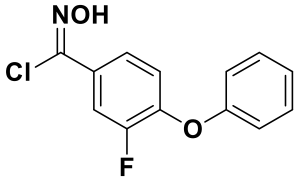

3-fluoro-*N*-hydroxy-4-phenoxybenzimidoyl chloride (**17h**) was obtained in 83% yield. ^{1}H NMR (300 MHz, $CDCl_3$) δ 6.98-7.02 (m, 3H), 7.11-7.16 (m, 1H), 7.32-7.39 (m, 2H), 7.56 (d, 1H, *J* 8.7 Hz), 7.67 (dd, 1H, *J* 2.1 and 11.5 Hz), 7.95 (s, 1H).

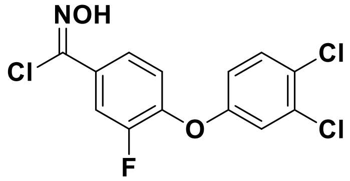

4-(3,4-dichlorophenoxy)-3-fluoro-*N*-hydroxybenzimidoyl chloride (**17k**) was obtained in 76% yield. ^{1}H NMR (300 MHz, $CDCl_3$) δ 6.85 (dd, 1H, *J* 2.8 and 8.8 Hz), 7.03-7.08 (m, 2H), 7.38 (d, 1H, *J* 8.8 Hz), 7.62 (d, 1H, *J* 8.8 Hz), 7.69 (dd, 1H, *J* 2.0 and 11.4 Hz), 8.10 (s, 1H).

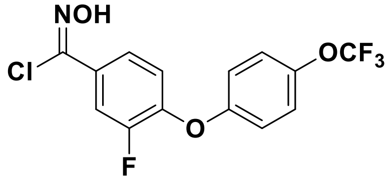

3-fluoro-N-hydroxy-4-(4-(trifluoromethoxy)phenoxy)benzimidoyl chloride (**17l**) was obtained in 70% yield. ^{1}H NMR (300 MHz, $CDCl_3$) δ 6.98-7.08 (m, 3H), 7.17-7.24 (m, 2H), 7.60 (d, 1H, *J* 8.6 Hz), 7.69 (dd, 1H, *J* 2.0 and 11.5 Hz), 7.89 (s, 1H).

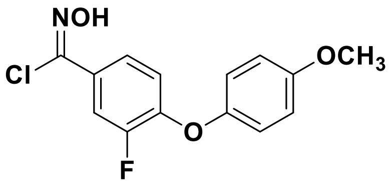

3-fluoro-*N*-hydroxy-4-(4-methoxyphenoxy)benzimidoyl chloride (**17n**) was obtained in 60% yield. ^{1}H NMR (300 MHz, $CDCl_3$) δ 3.79 (s, 3H), 6.85-6.89 (m, 3H), 6.96-6.99 (m, 2H), 7.50 (d, 1H, *J* 8.0 Jz), 7.64 (dd, 1H, *J* 2.1 and 11.7 Hz), 8.03 (s, 1H).

Synthesis of diaryl ether and thioether benzonitriles 19i-j, o-p

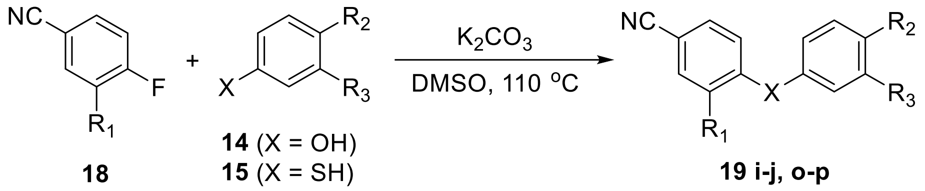

Fluorobenzonitrile derivatives **18** (1.0 eq., 20 mmol), phenols **14**/ thiols **15** with substituents of interest (1.0 eq., 20 mmol), potassium carbonate (4.15 g, 1.5 eq., 30 mmol) and dimethylsulfoxide (20 mL) were poured into a flask equipped with magnetic stirring and a reflux condenser. The mixture was heated to 110 °C and the conversion of the phenolic compound to the desired product was monitored by TLC analyses. Upon completion, the reaction medium was dissolved in ethyl acetate (150 mL) and washed with distilled water (100 mL). The aqueous phase was extracted twice with ethyl acetate (50 mL). The organic phases were combined, dried over anhydrous magnesium sulfate, filtered and concentrated under reduced pressure to give the benzonitrile product as a white solid.[2]

The obtained characterizing data are consistent with literature information for 4-(4-chlorophenoxy)-3-fluorobenzonitrile (**19j**)[8], 4-(phenylthio)benzonitrile (**19o**)[9], 4-((4-chlorophenyl)thio)benzonitrile (**19p**)[10].

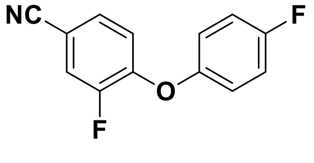

3-fluoro-4-(4-fluorophenoxy)benzonitrile (**19i**) was obtained in 98% yield. ^{1}H NMR (300 MHz, $CDCl_3$) δ 6.86-6.92 (m, 1H), 7.02-7.08 (m, 4H), 7.33-7.37 (m, 1H), 7.45 (dd, 1H, *J* 1.8 and 10.1 Hz).

Synthesis of diaryl ether and thioether amidoximes 20i-j, o-p

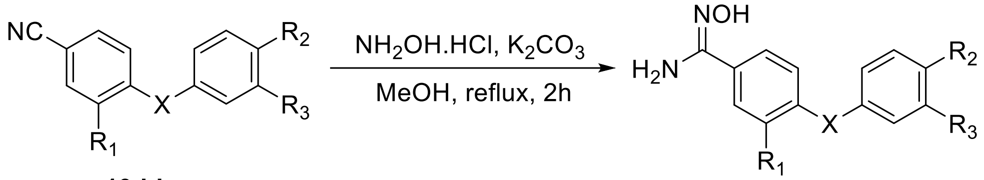

19 i-j, o-p **20i-j, o-p**

Following a modified literature procedure[1], potassium carbonate (3.11 g, 1.5 eq., 22.5 mmol) and hydroxylamine hydrochloride (2.60 g, 2.5 eq., 37.5 mmol,) were added to a solution of nitriles **19i-j, 17o-p** (1.0 eq., 15 mmol) in dry methanol, and the mixture was agitated at reflux for 2 hours under a nitrogen atmosphere. The solution was then vacuum-concentrated, diluted with water (50 mL, and extracted using ethyl acetate (3 x 30 mL). The organic phases were dried with MgSO4, filtered, and concentrated in vacuo to yield amidoximes.

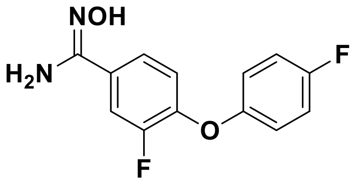

3-fluoro-4-(4-fluorophenoxy)-N'-hydroxybenzimidamide (**20i**) was obtained in 78% yield. ^{1}H NMR (300 MHz, CDCl3) δ 4.89 (s, 2H) 7.04-7.11 (m, 4H), 7.36 (m, 1H), 7.52 (m, 2H).

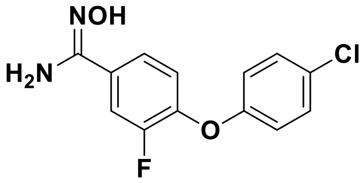

4-(4-chlorophenoxy)-3-fluoro-N'-hydroxybenzimidamide (**20j**) was obtained in 85% yield. ^{1}H NMR (300 MHz, DMSO-*d6*) δ 5.87 (s, 2H), 7.05 (m, 2H), 7.38-7.43 (m, 3H), 7.70 (d, 1H, *J* 7.9 Hz), 7.82 (dd, 1H, *J* 1.3 and 11.2 Hz), 9.75 (s, 1H).

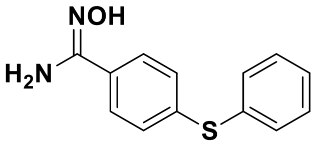

N'-hydroxy-4-(phenylthio)benzimidamide **(20o)** was obtained in 74% yield. ^{1}H NMR (300 MHz, CDCl3) δ 4.86 (s, 2H), 7.22-.725 (m, 2H), 7.30-7.32 (m, 2H), 7.34-7.40 (m, 3H), 7.47-7.49 (m, 2H).

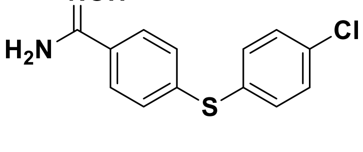

(Z)-4-((4-chlorophenyl)thio)-N'-hydroxybenzimidamide **(20p)** was obtained in 89% yield. ^{1}H NMR (300 MHz, CDCl3) δ 4.81 (s, 2H), 7.23-7.28 (m, 6H), 7.52 (d, 2H, *J* 8.5 Hz).

Synthesis of diaryl ether and thioether chloro-oximes 17i-j, o-p

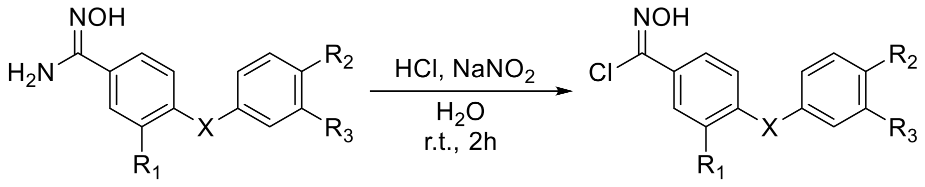

20i-j, o-p **17i-j, o-p**

Amidoximes **20i-j**, **o-p** (10 mmol, 1 eq.) were dissolved in a mixture of concentrated hydrochloric acid (10 mL, 1 eq.) and water (50 mL) at room temperature. Sodium nitrite (11.5 mmol, 1.15 eq.) was dissolved in 5 mL of water and then was added dropwise to the mixture. The system was stirred for 2 h. At the end of the reaction, as verified by TLC, the system was neutralized with sodium bicarbonate and the material was vacuum filtered and washed with water. The products were used in the next step without further purification.[1]

References

[1] D. B. Carvalho, P. A. N. Costa, G. B. Portapilla, A. R. das Neves, C. Y. K. Shiguemoto, B. I. Pelizaro, F. Silva, E. M. Piranda, C. C. P. Arruda, P. D. M. Gaspari, I. A. Cardoso, P. H. Luccas, M. C. Nonato, N. P. Lopes, S. de Albuquerque, A. C. M. Baroni, ""Design, synthesis and antitrypanosomatid activity of 2-nitroimidazole-3,5-disubstituted isoxazole compounds based on benznidazole,"" *European Journal of Medicinal Chemistry* **2023**, *260*, 115451.

[2] R. P. Tangallapally, R. Yendapally, R. E. Lee, A. J. M. Lenaerts, R. E. Lee, ""Synthesis and Evaluation of Cyclic Secondary Amine Substituted Phenyl and Benzyl Nitrofuranyl Amides as Novel Antituberculosis Agents,"" *J. Med. Chem.* **2005**, *48*, 8261–8269.

[3] Y. Du, F. Yao, Y. Tuo, M. Cai,""Highly Efficient Heterogeneous Copper-Catalysed O-Arylation of Phenols by Nitroarenes Leading to Diaryl Ethers,"" Journal of Medicinal Research, **2017**, *41*, 725-729.

[4] R. Takano, M. Yoshida, M. Inoue, T. Honda, R. Nakashima, K. Matsumoto, T. Yano, T. Ogata, N. Watanabe, M. Hirouchi, T. Kimura, N. Toda, ""Optimization of 3-aryl-3-ethoxypropanoic acids and discovery of the potent GPR40 agonist DS-1558,"" *Bioorganic & Medicinal Chemistry* **2015**, *23*, 5546–5565.

[5] T. Strittmatter, A. Brockmann, M. Pott, A. Hantusch, T. Brunner, A. Marx, ""Expanding the Scope of Human DNA Polymerase λ and β Inhibitors,"" *ACS Chem. Biol.* **2014**, *9*, 282–290.

[6] X. Jiang, X. Xu, Y. Lin, Y. Yan, P. Li, R. Bai, Y. Xie, ""A mild system for synthesis of aldoximes and ketoximes in the presence of *N*-hydroxyphthalimide in aqueous system,"" *Tetrahedron* **2018**, *74*, 5879–5885.

[7] K. Yang, F. Zhang, T. Fang, G. Zhang, Q. Song, ""Stereospecific 1,4-Metallate Shift Enables Stereoconvergent Synthesis of Ketoximes,"" *Angewandte Chemie International Edition* **2019**, *58*, 13421–13426.

[8] T. G. Le, A. Kundu, A. Ghoshal, N. H. Nguyen, S. Preston, Y. Jiao, B. Ruan, L. Xue, F. Huang, J. Keiser, A. Hofmann, B. C. H. Chang, J. Garcia-Bustos, T. N. C. Wells, M. J. Palmer, A. Jabbar, R. B. Gasser, J. B. Baell, ""Structure–Activity Relationship Studies of Tolfenpyrad Reveal Subnanomolar Inhibitors of Haemonchus contortus Development,"" *J. Med. Chem.* **2019**, *62*, 1036–1053.

[9] D. J. C. Prasad, G. Sekar, ""Cu-Catalyzed One-Pot Synthesis of Unsymmetrical Diaryl Thioethers by Coupling of Aryl Halides Using a Thiol Precursor,"" *Org. Lett.* **2011**, *13*, 1008–1011.

[10] G. Kibriya, S. Mondal, A. Hajra, ""Visible-Light-Mediated Synthesis of Unsymmetrical Diaryl Sulfides via Oxidative Coupling of Arylhydrazine with Thiol,"" *Org. Lett.* **2018**, *20*, 7740–7743.

^{1}H NMR and ^{13}C NMR spectra of compounds

5-((2-nitro-1H-imidazol-1-yl)methyl)-3-(4-phenoxyphenyl)isoxazole (6a)

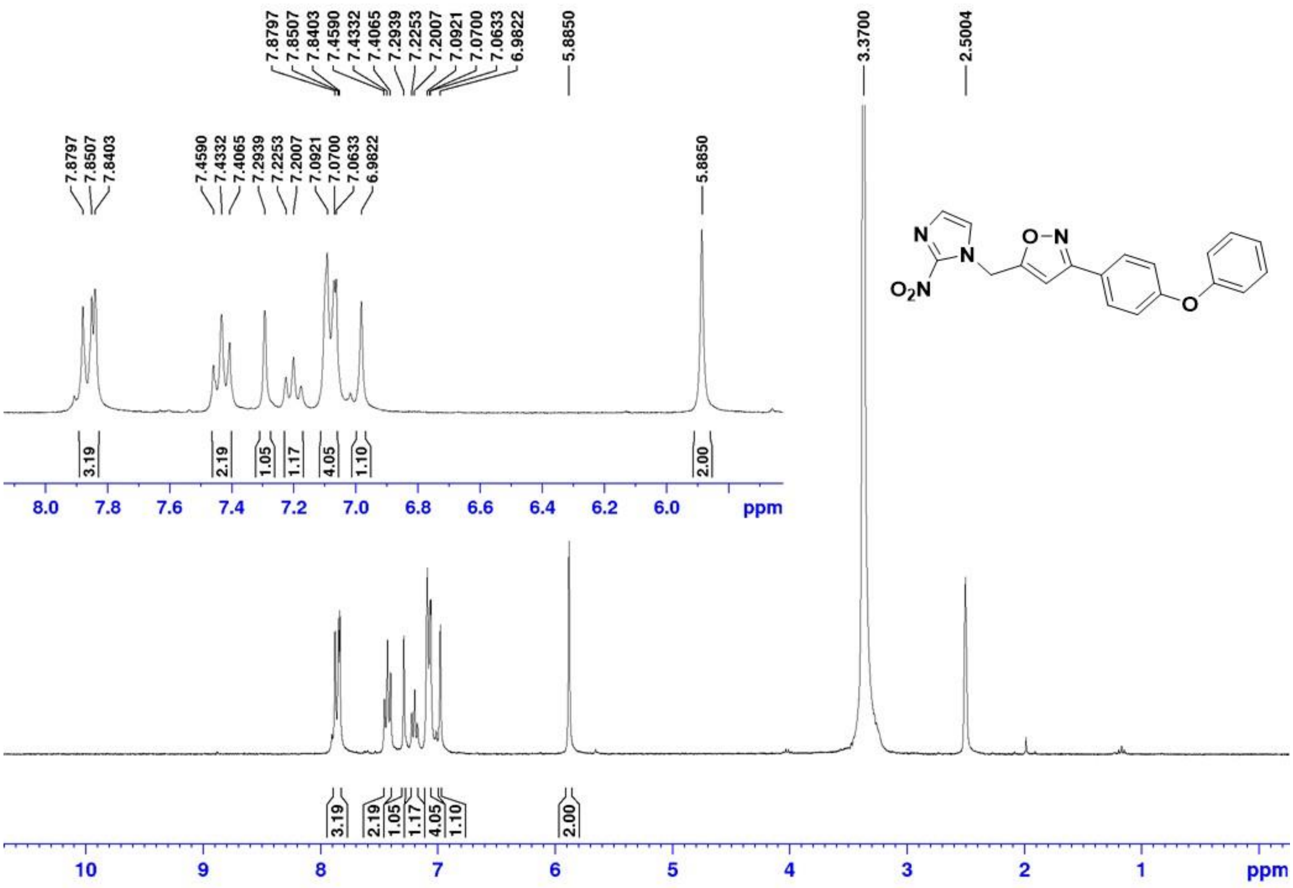

Figure S1. ^{1}H NMR spectrum (300 MHz, DMSO-d_6) of compound **6a**.

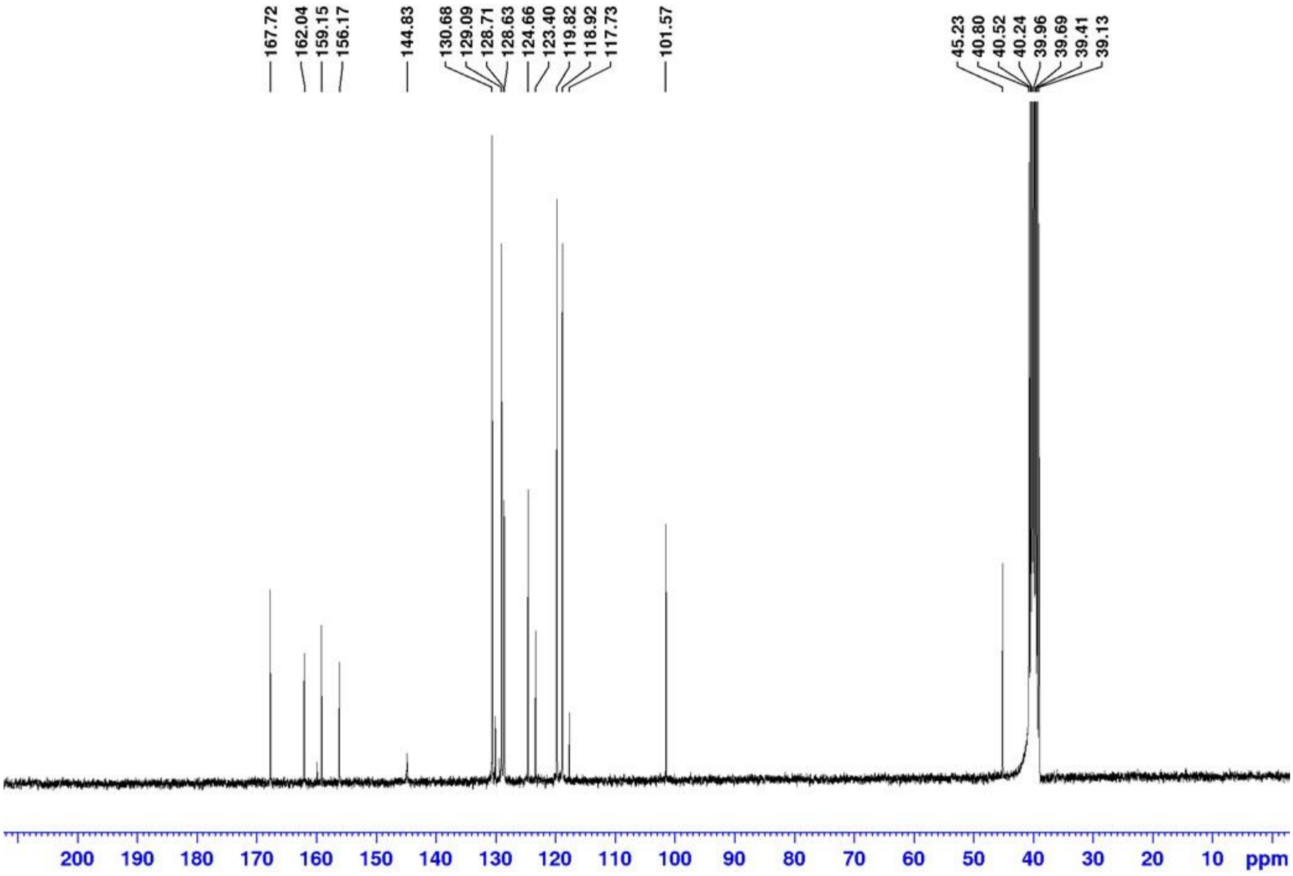

Figure S2. ^{13}C NMR spectrum (75 MHz, DMSO-d_6) of compound **6a**.

3-(4-(4-fluorophenoxy)phenyl)-5-((2-nitro-1H-imidazol-1-yl)methyl)isoxazole (6b)

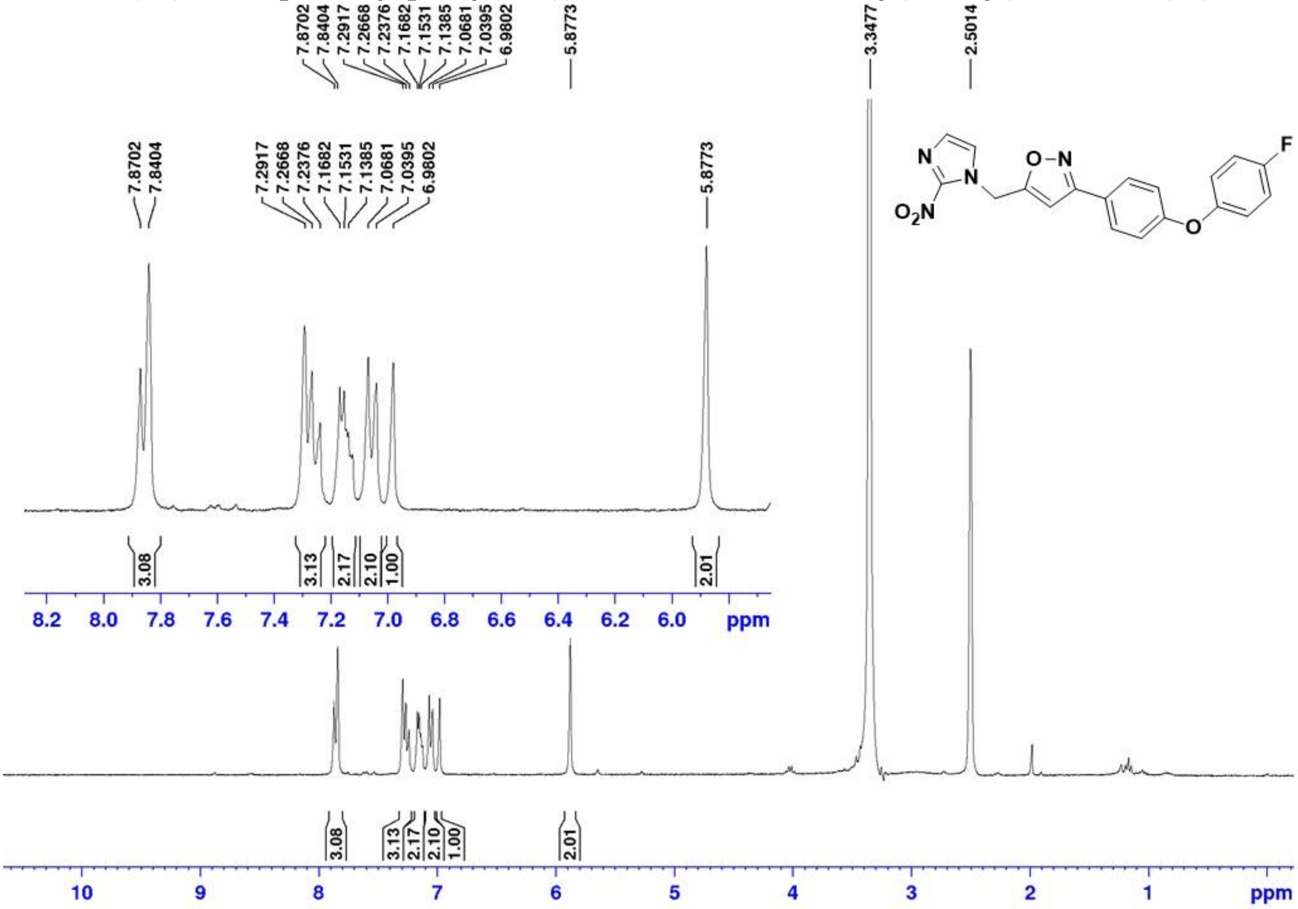

Figure S3. ^{1}H NMR spectrum (300 MHz, DMSO-d_6) of compound **6b**.

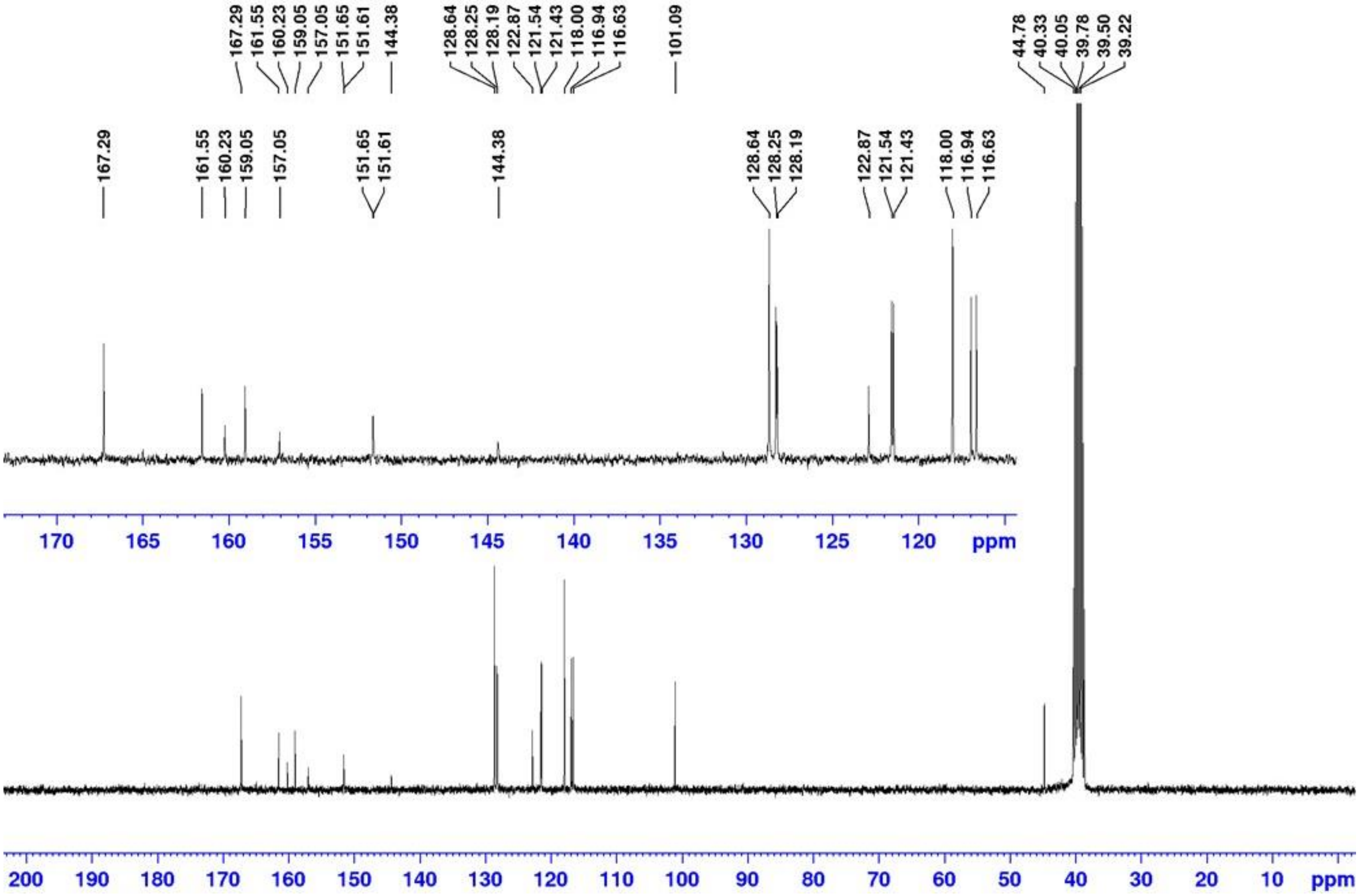

Figure S4. ^{13}C NMR spectrum (75 MHz, DMSO- d_6) of compound **6b**.

3-(4-(4-chlorophenoxy)phenyl)-5-((2-nitro-1H-imidazol-1-yl)methyl)isoxazole (6c)

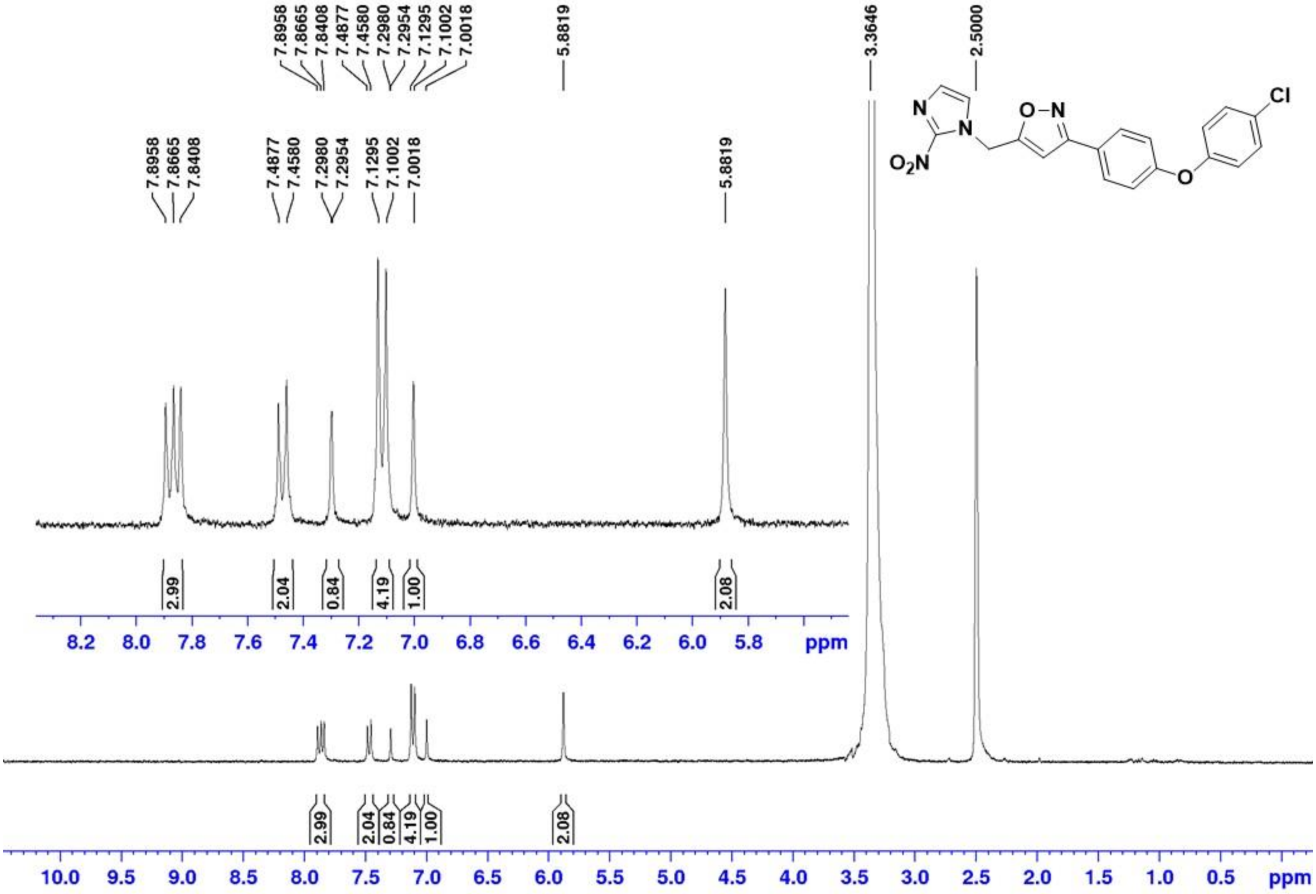

Figure S5. ^{1}H NMR spectrum (300 MHz, DMSO- d_6) of compound **6c**.

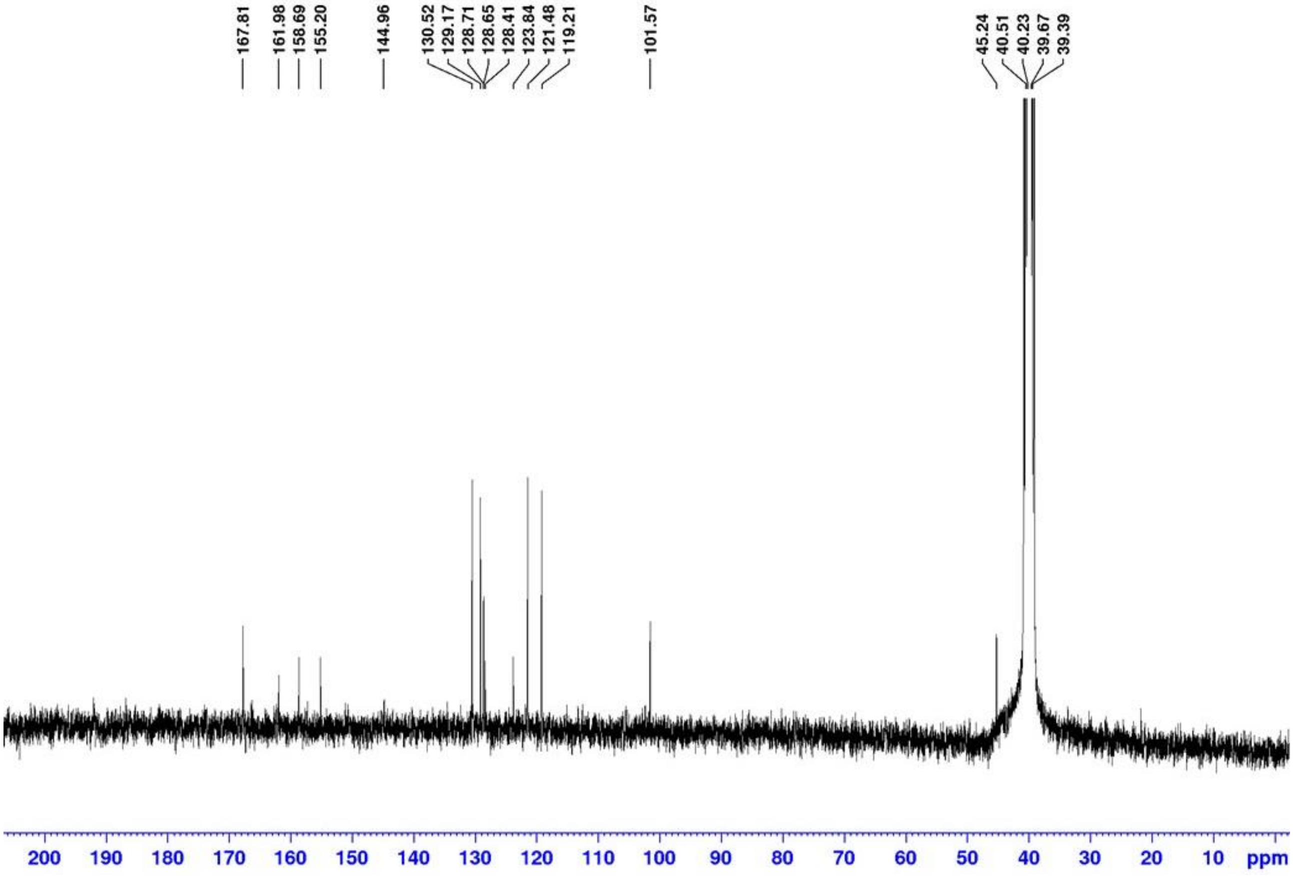

Figure S6. ^{13}C NMR spectrum (75 MHz, DMSO- d_6) of compound **6c**.

3-(4-(3,4-dichlorophenoxy)phenyl)-5-((2-nitro-1H-imidazol-1-yl)methyl)isoxazole (6d)

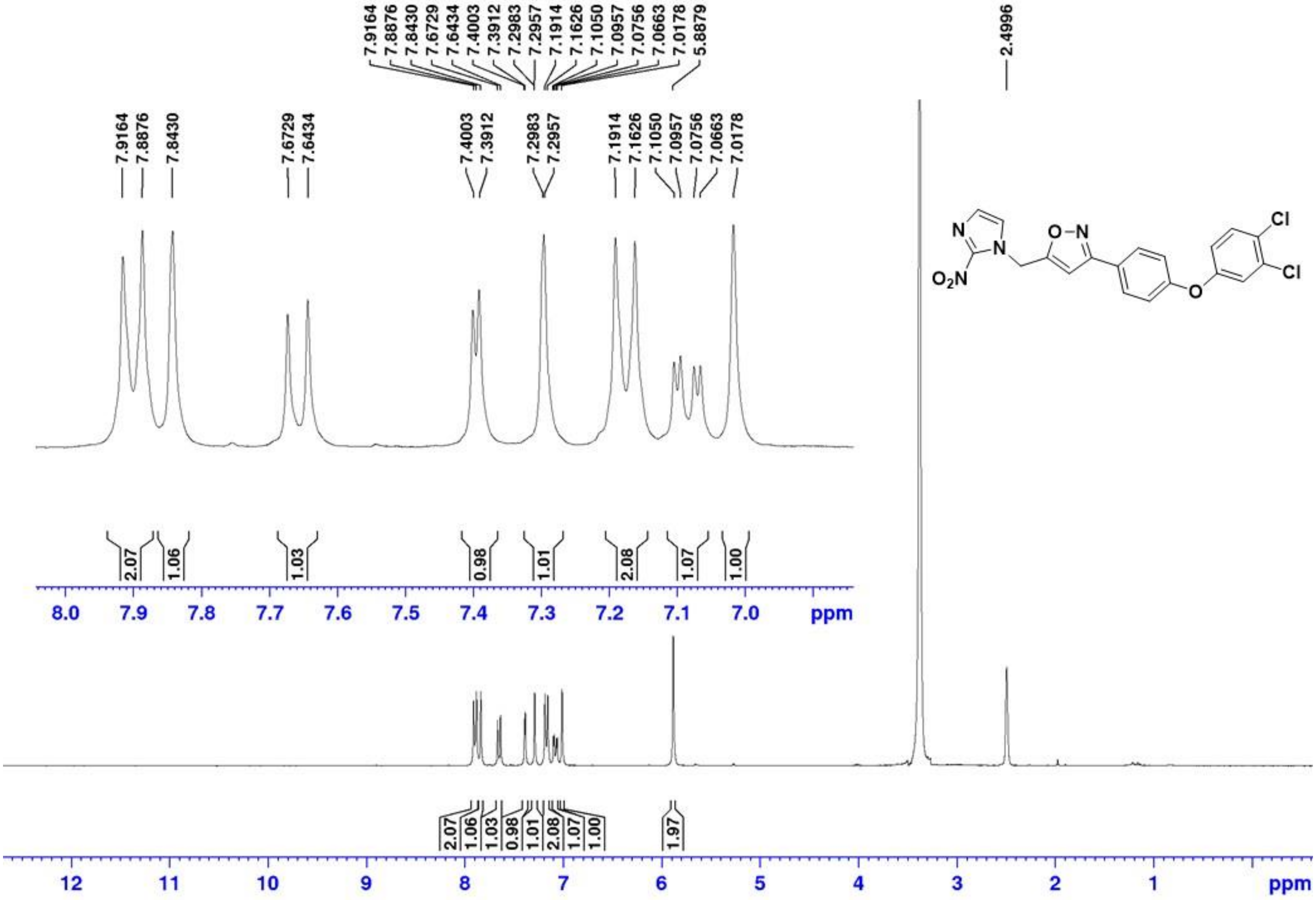

Figure S7. ^{1}H NMR spectrum (300 MHz, DMSO-d_6) of compound **6d**.

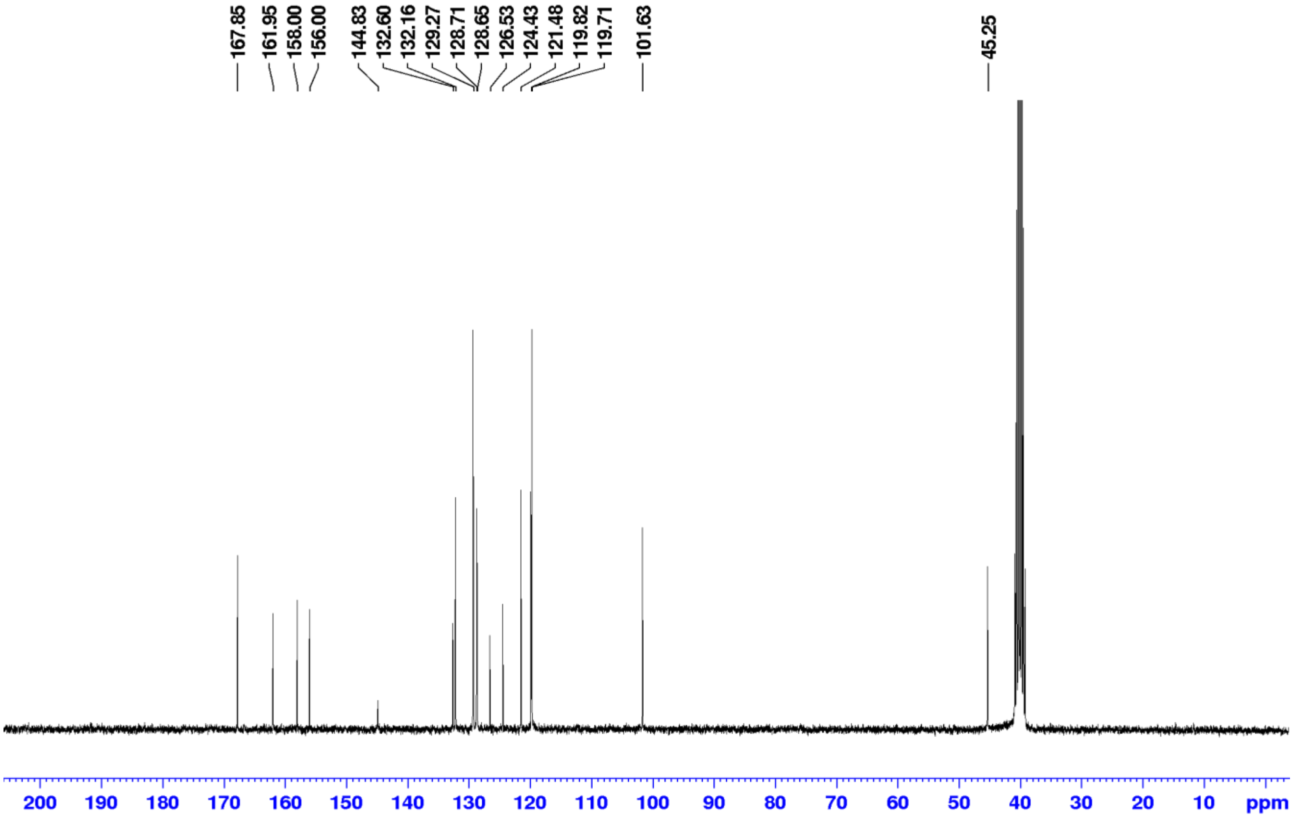

Figure S8. ^{13}C NMR spectrum (75 MHz, DMSO-d_6) of compound **6d**.

5-((2-nitro-1H-imidazol-1-yl)methyl)-3-(4-(4-(trifluoromethoxy)phenoxy)phenyl)isoxazole (6e)

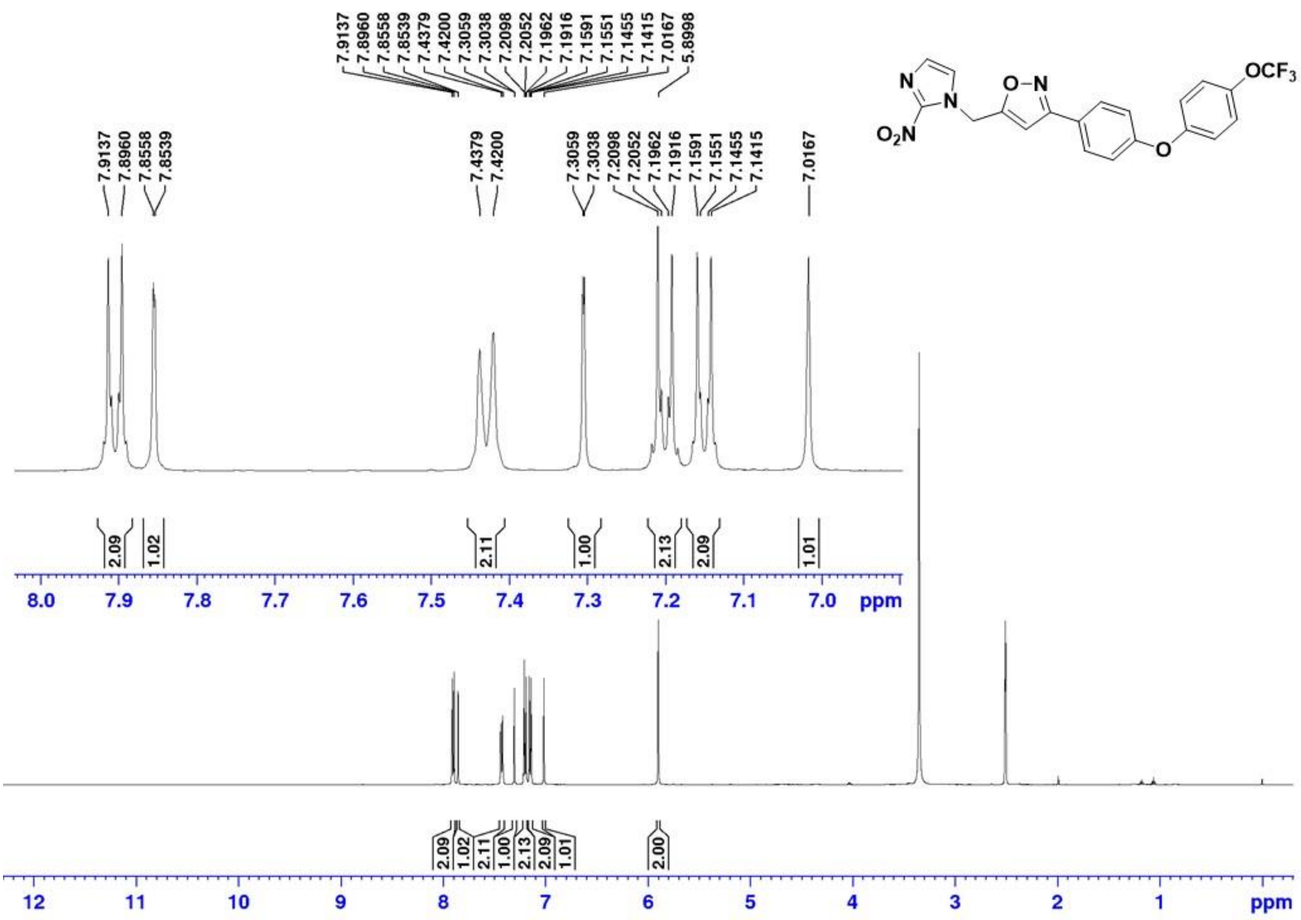

Figure S9. ^{1}H NMR spectrum (500 MHz, DMSO-d_6) of compound **6e**.

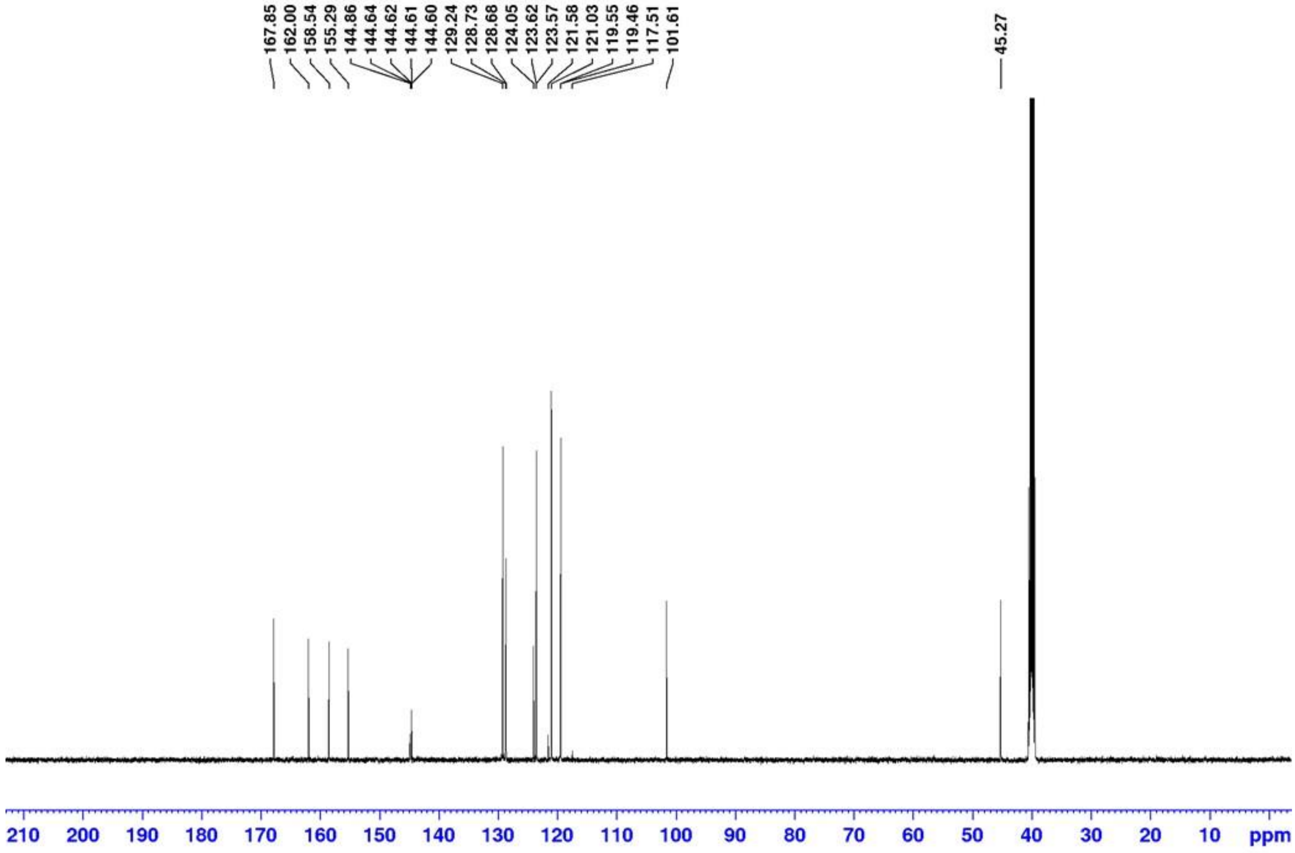

Figure S10. ^{13}C NMR spectrum (100 MHz, DMSO-d_6) of compound **6e**.

5-((2-nitro-1H-imidazol-1-yl)methyl)-3-(4-(p-tolyloxy)phenyl)isoxazole (6f)

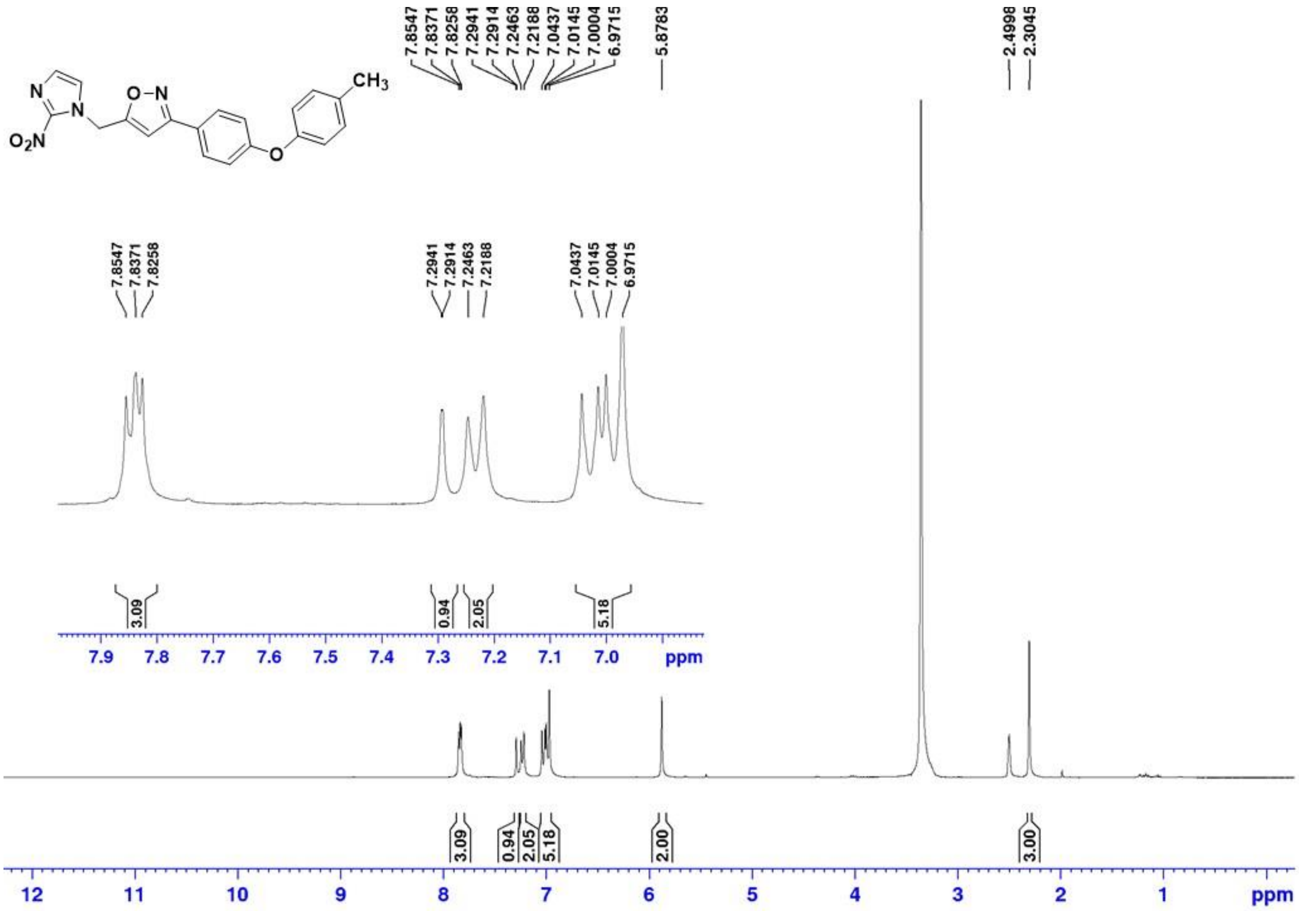

Figure S11. ^{1}H NMR spectrum (300 MHz, DMSO-d_6) of compound **6f**.

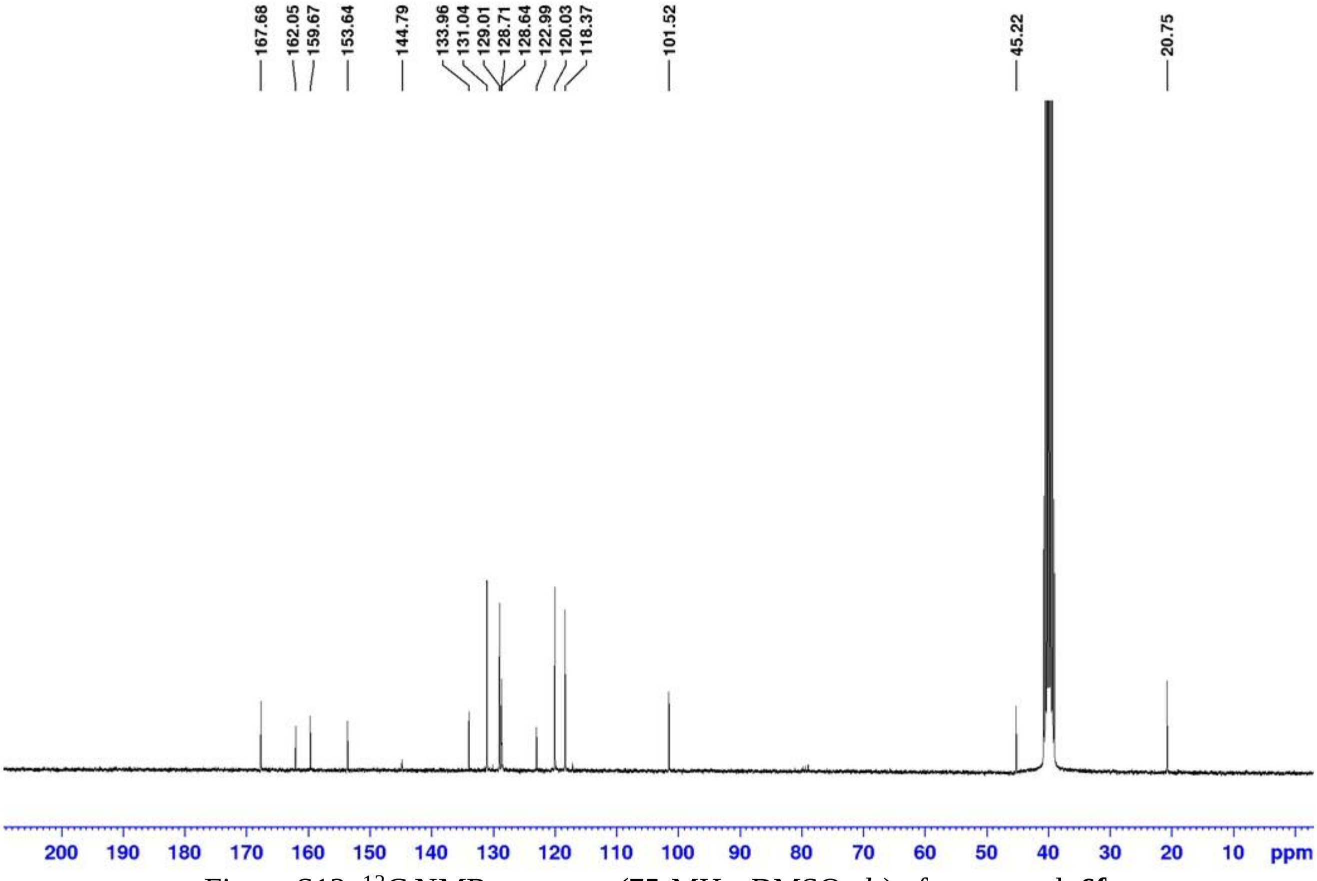

Figure S12. ^{13}C NMR spectrum (75 MHz, DMSO-d_6) of compound **6f.**

3-(4-(4-methoxyphenoxy)phenyl)-5-((2-nitro-1H-imidazol-1-yl)methyl)isoxazole (6g)

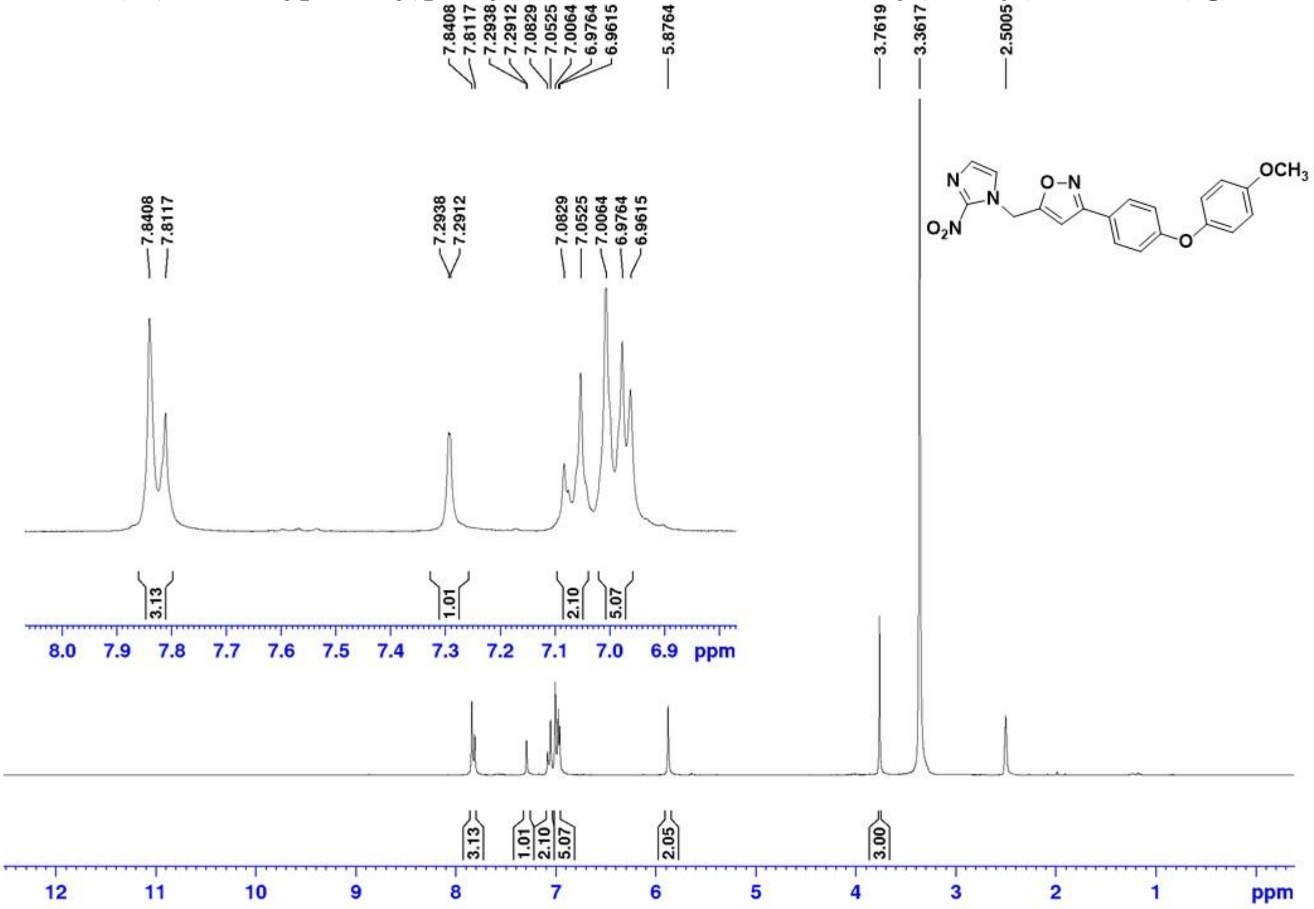

Figure S13. Figure S9. ^{1}H NMR spectrum (300MHz, DMSO-d_6) of compound **6g**.

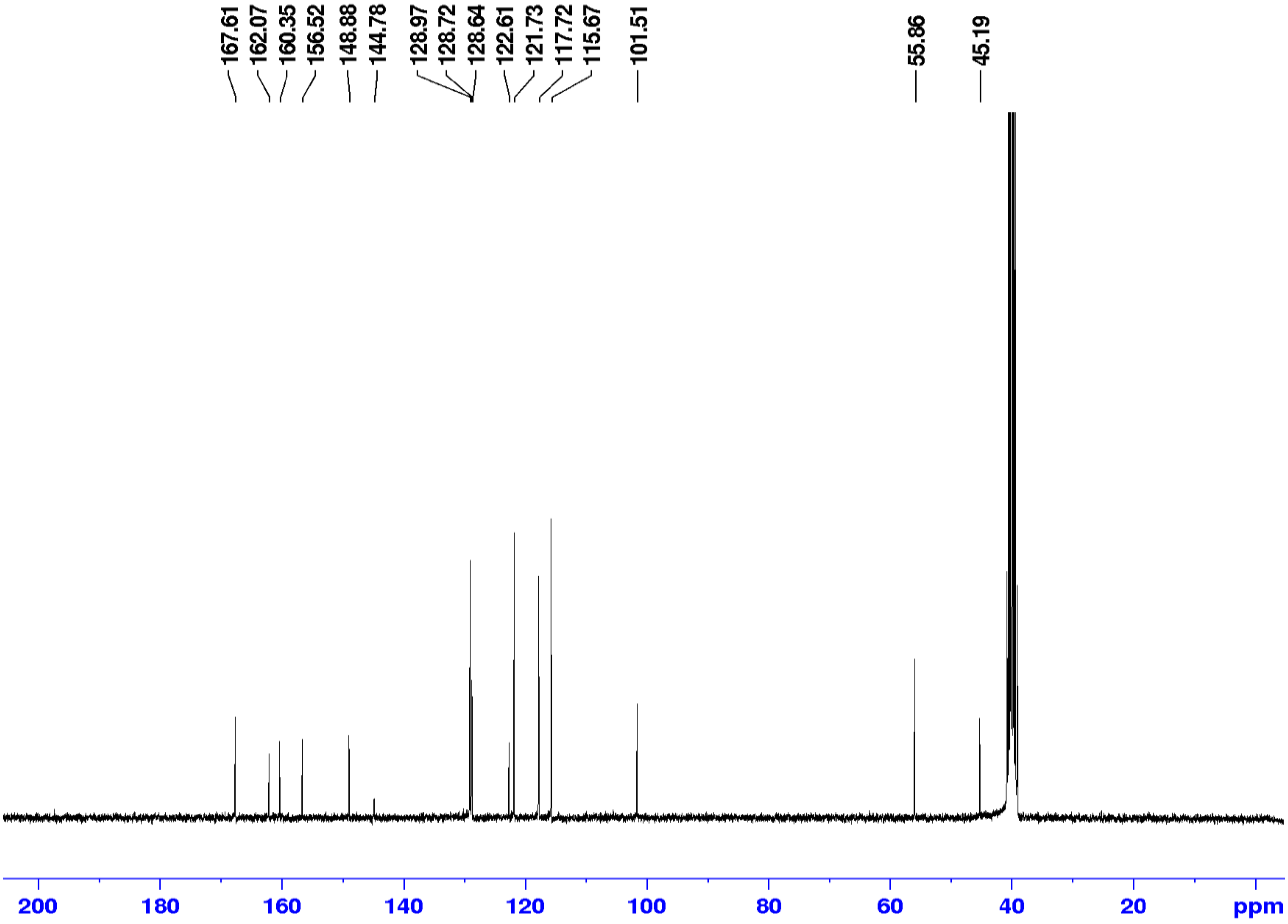

Figure S14. ^{13}C NMR spectrum (75 MHz, DMSO-d_6) of compound **6g.**

3-(3-fluoro-4-phenoxyphenyl)-5-((2-nitro-1H-imidazol-1-yl)methyl)isoxazole (6h)

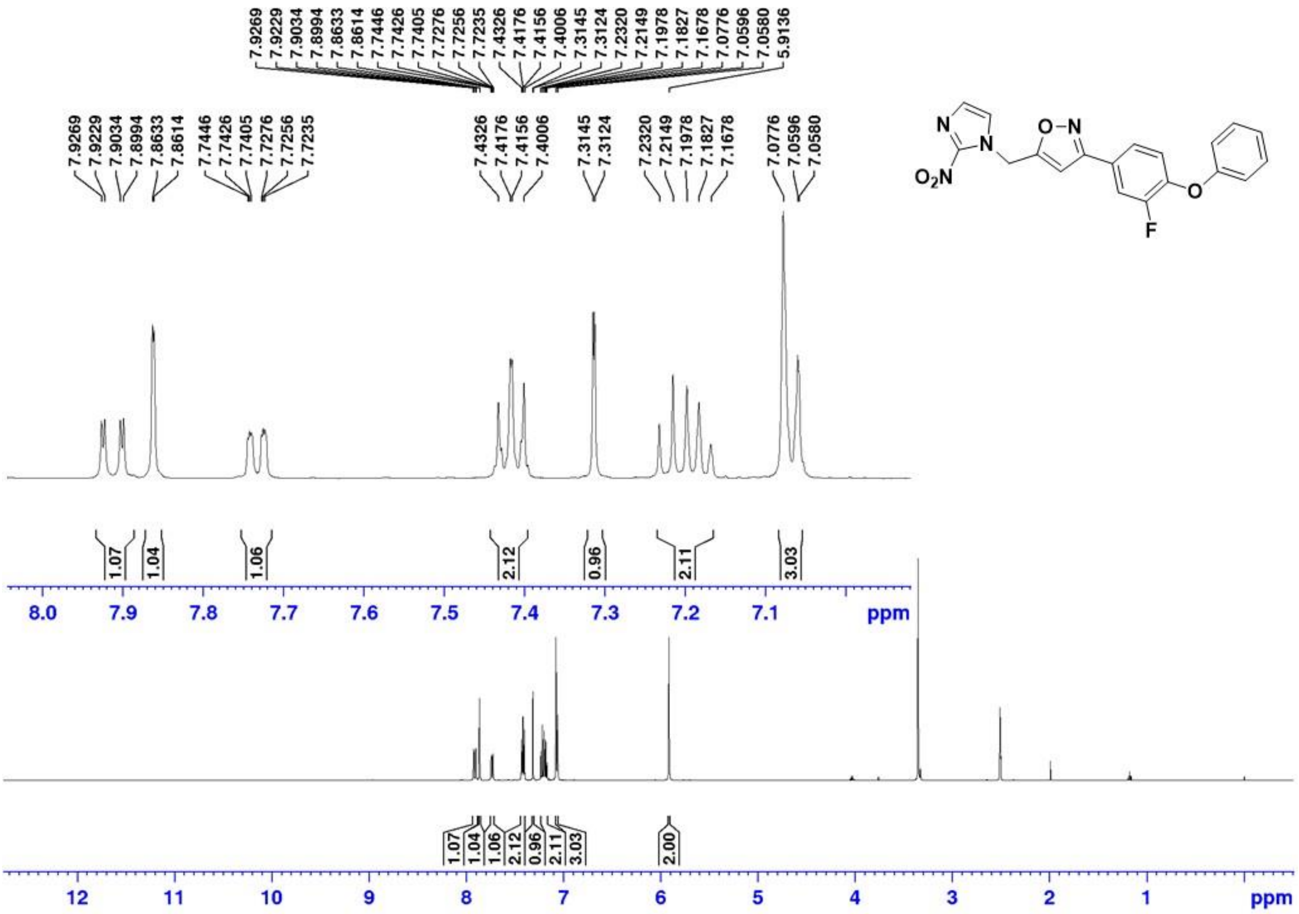

Figure S15. ^{1}H NMR spectrum (500 MHz, DMSO-d_6) of compound **6h**.

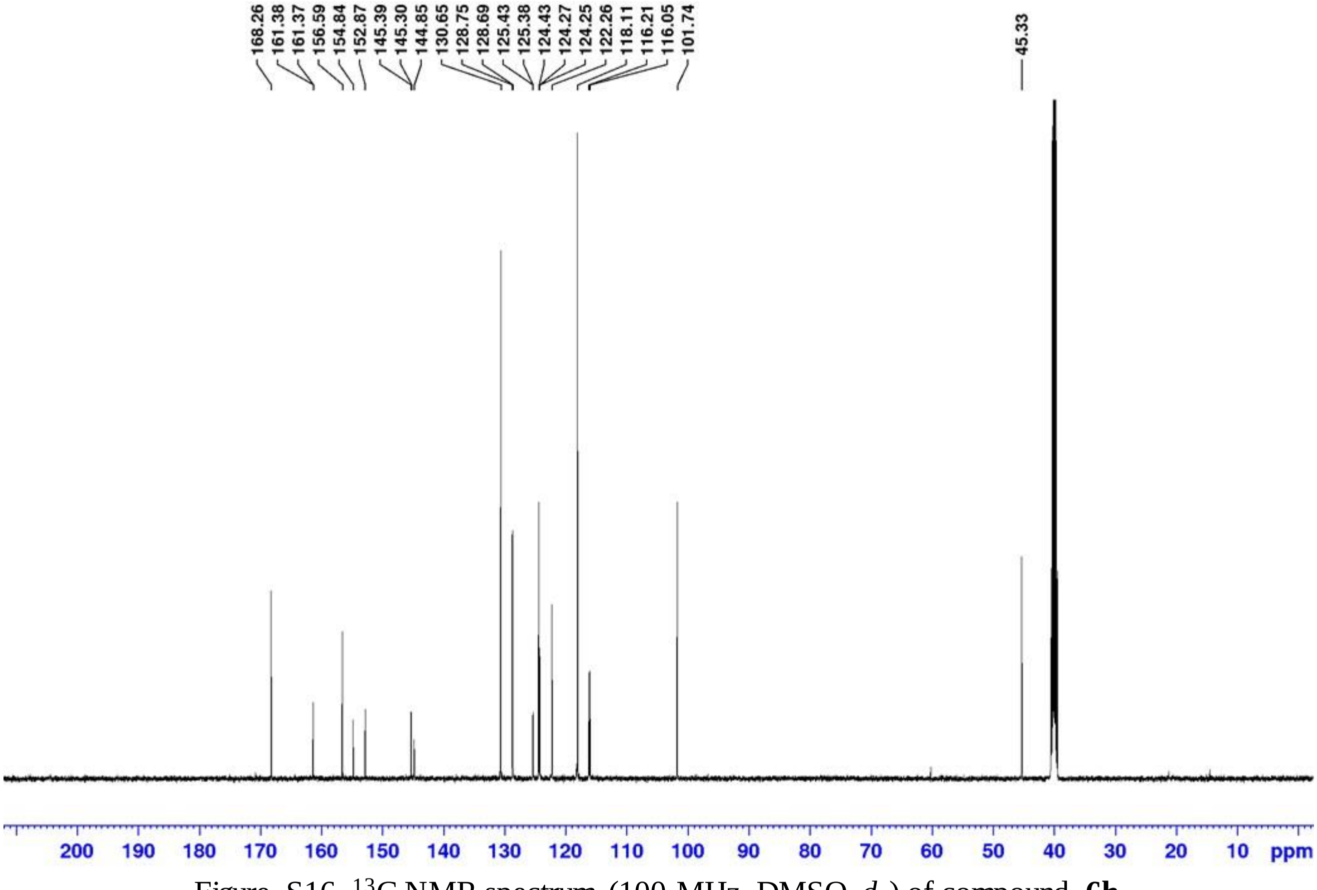

Figure S16. ^{13}C NMR spectrum (100 MHz, DMSO-d_6) of compound **6h.**

3-(3-fluoro-4-(4-fluorophenoxy)phenyl)-5-((2-nitro-1H-imidazol-1-yl)methyl)isoxazole (6i)

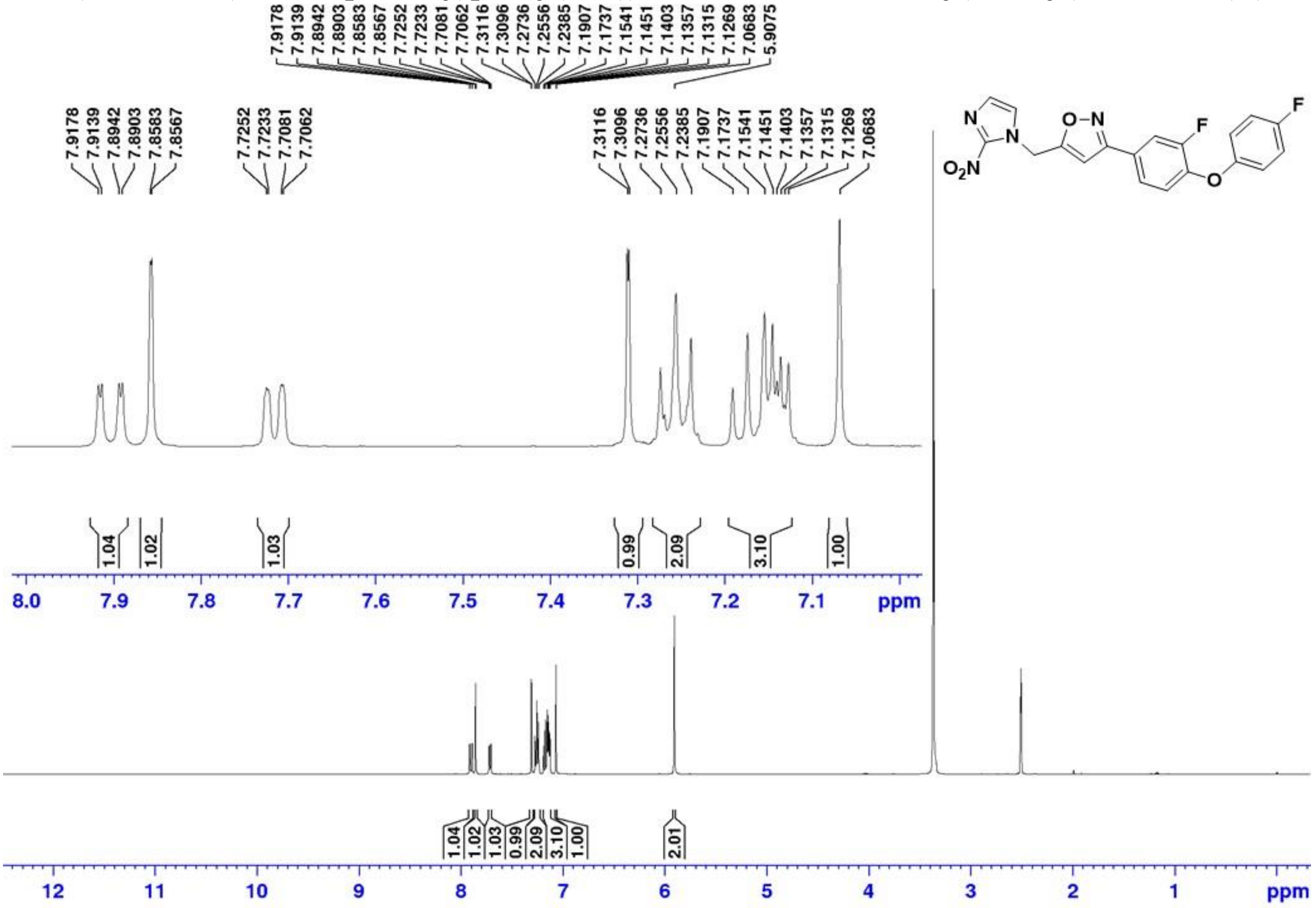

Figure S17. ^{1}H NMR spectrum (500 MHz, DMSO-d_6) of compound **6i**.

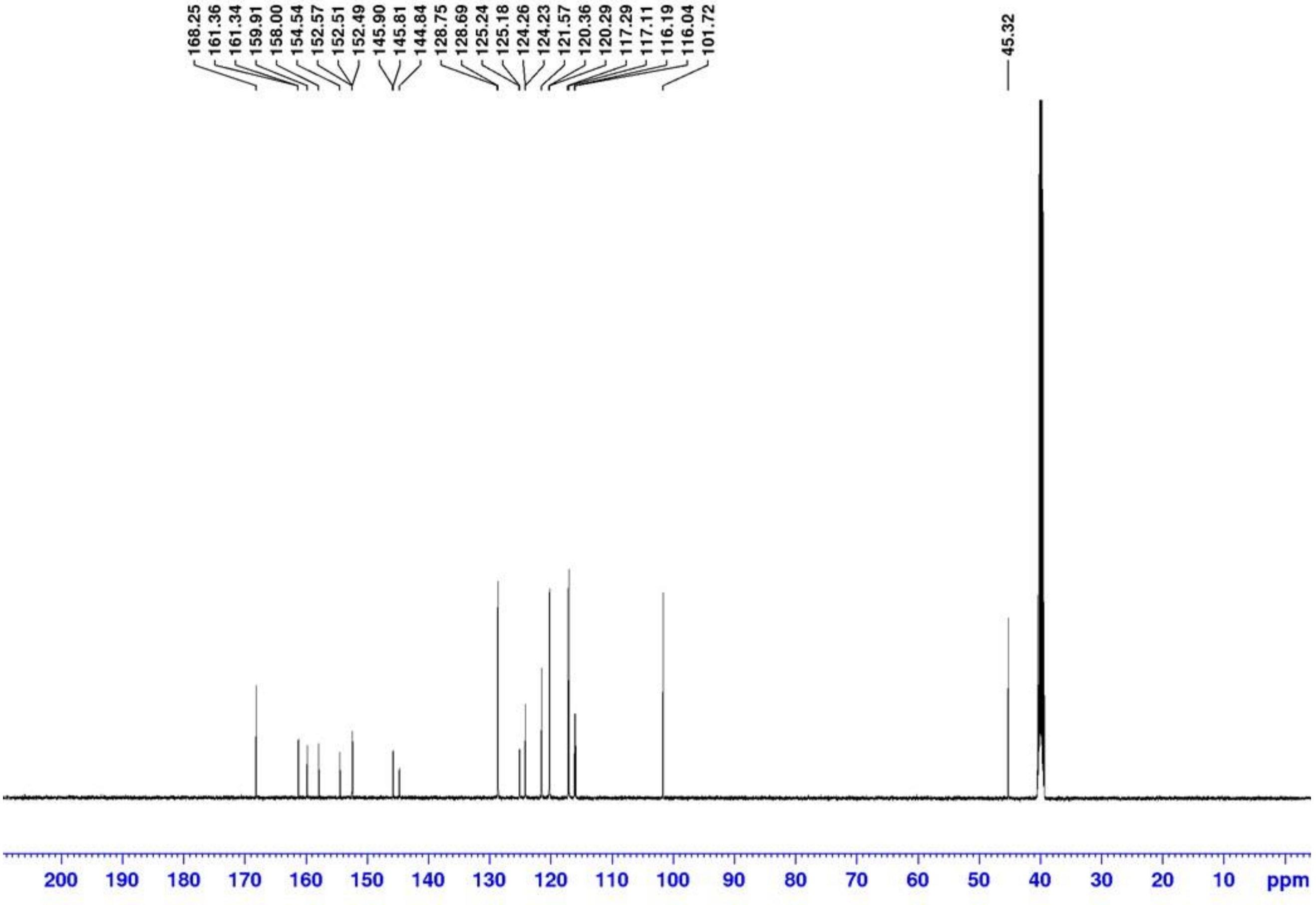

Figure S18. ^{13}C NMR spectrum (100 MHz, DMSO-d_6) of compound **6i**.

3-(4-(4-chlorophenoxy)-3-fluorophenyl)-5-((2-nitro-1H-imidazol-1-yl)methyl)isoxazole (6j)

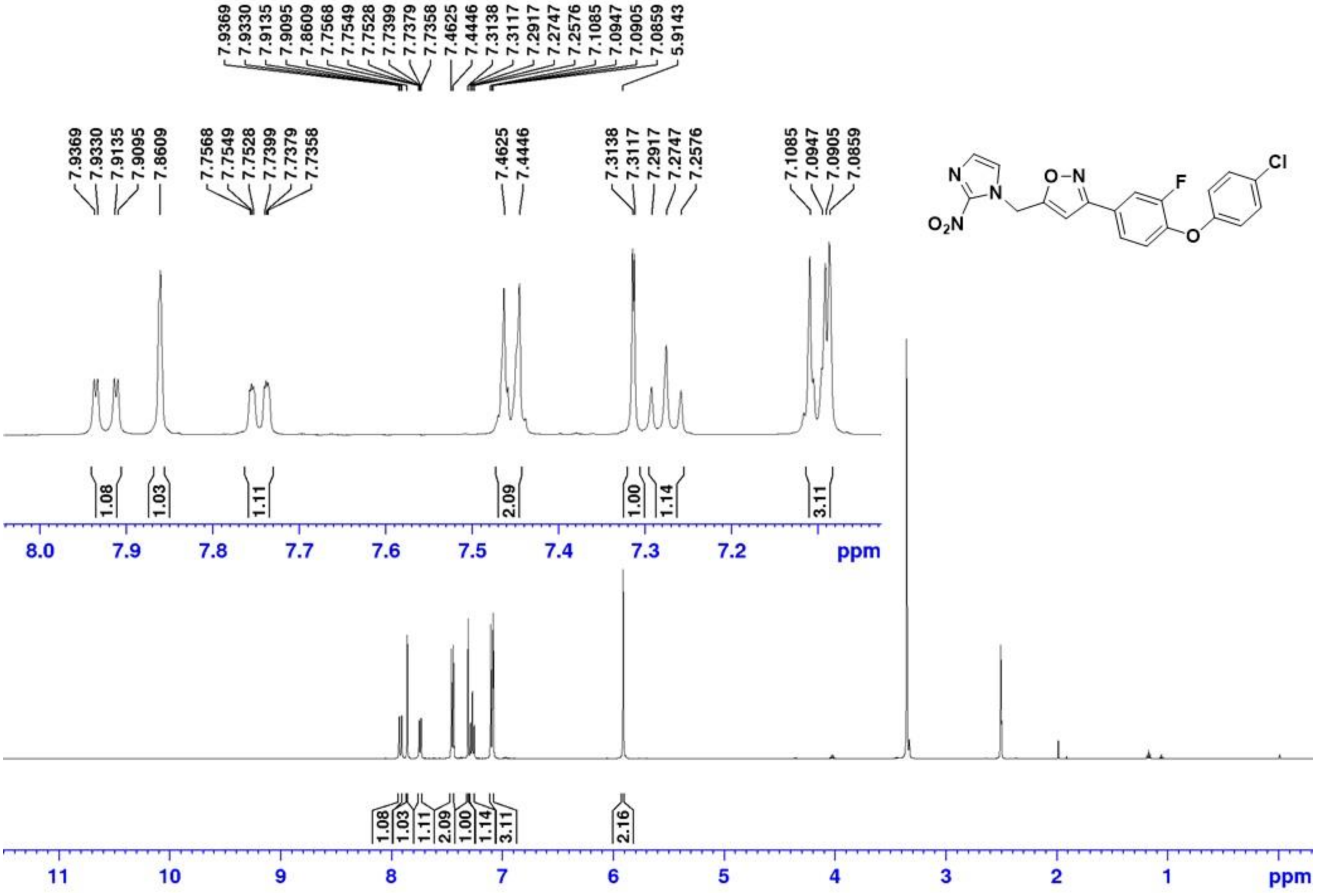

Figure S19. ^{1}H NMR spectrum (500 MHz, DMSO-d_6) of compound **6j**.

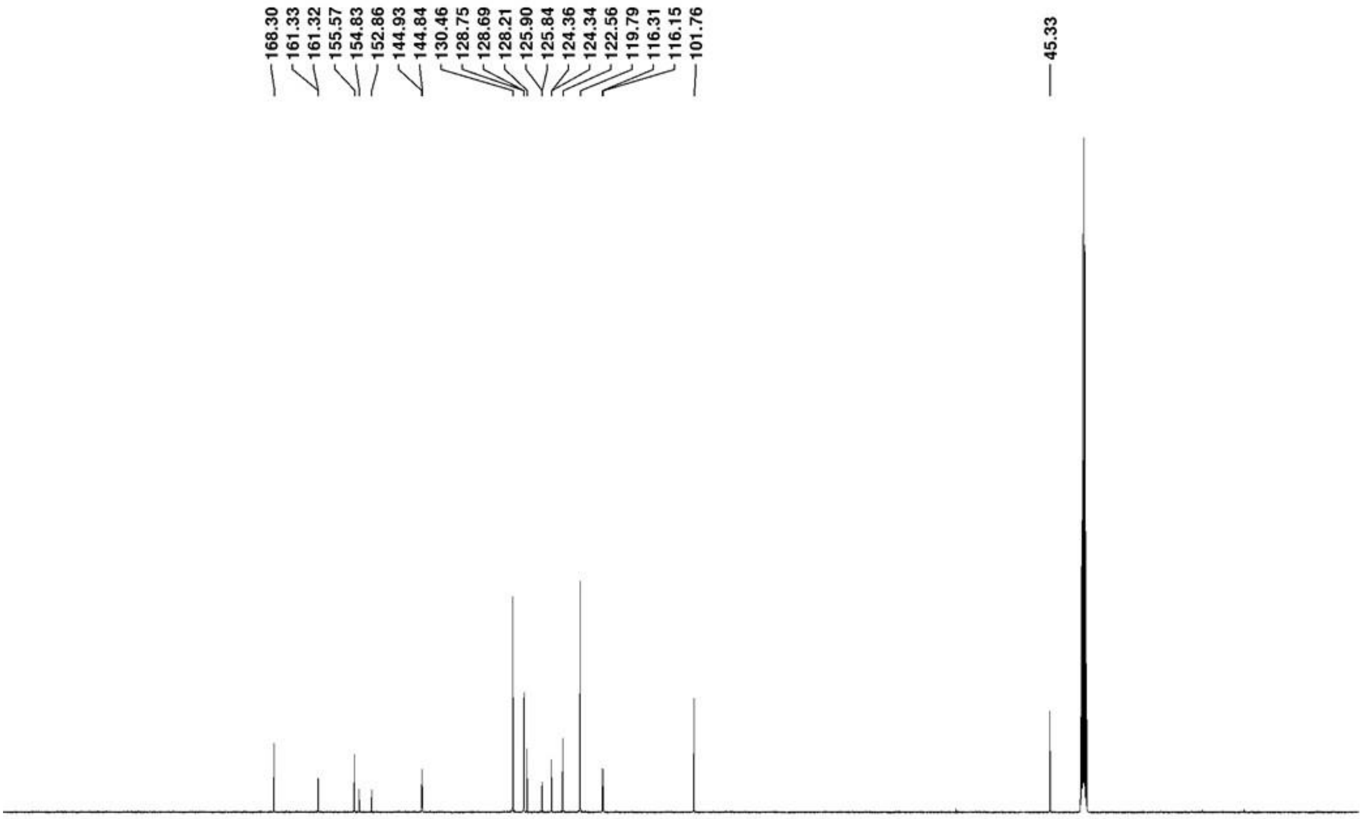

Figure S20. ^{13}C NMR spectrum (100 MHz, DMSO-$d6$) of compound **6j**.

3-(4-(3,4-dichlorophenoxy)-3-fluorophenyl)-5-((2-nitro-1H-imidazol-1-yl)methyl)isoxazole (6k)

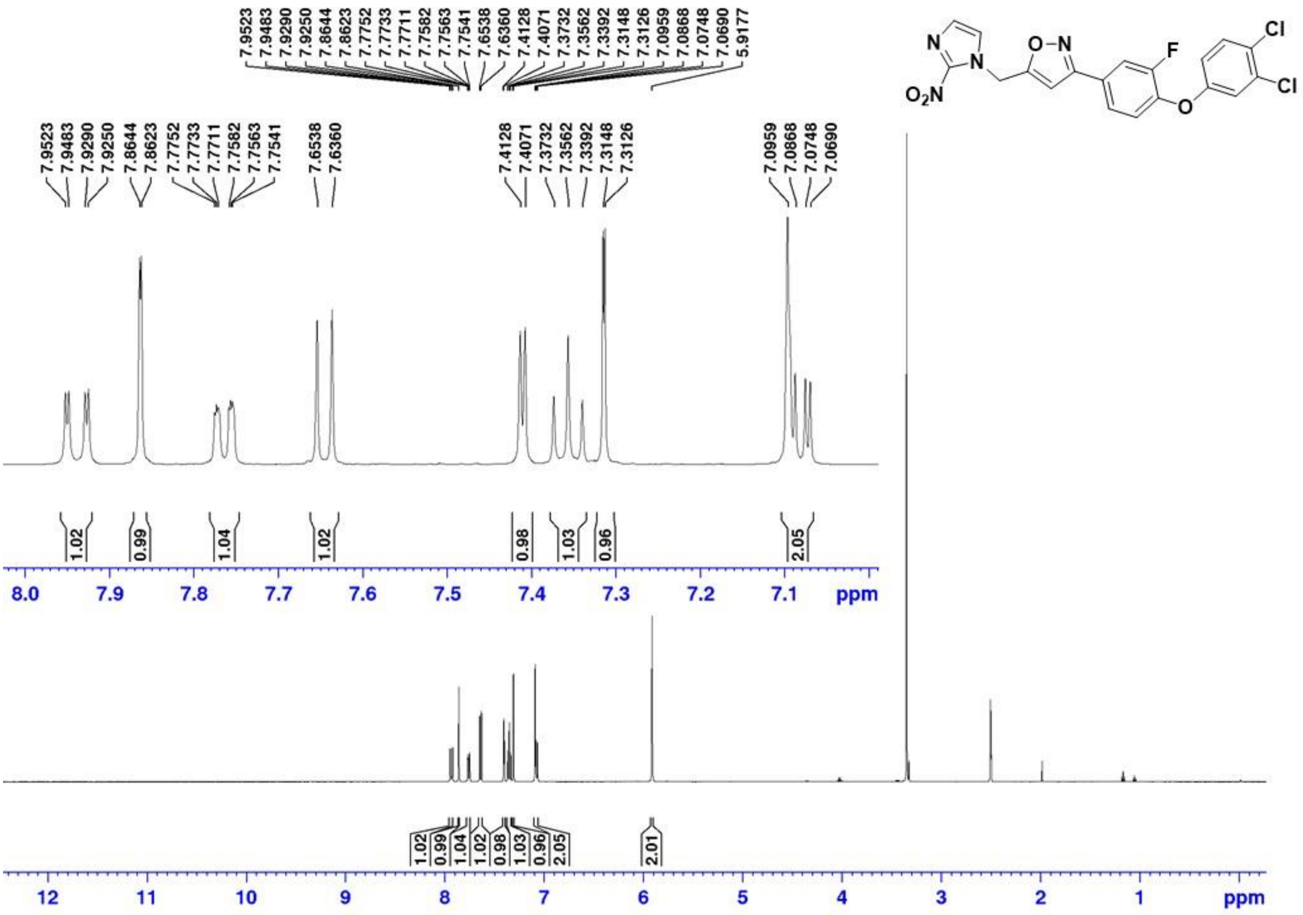

Figure S21. ^{1}H NMR spectrum (500 MHz, DMSO-d_6) of compound **6k**.

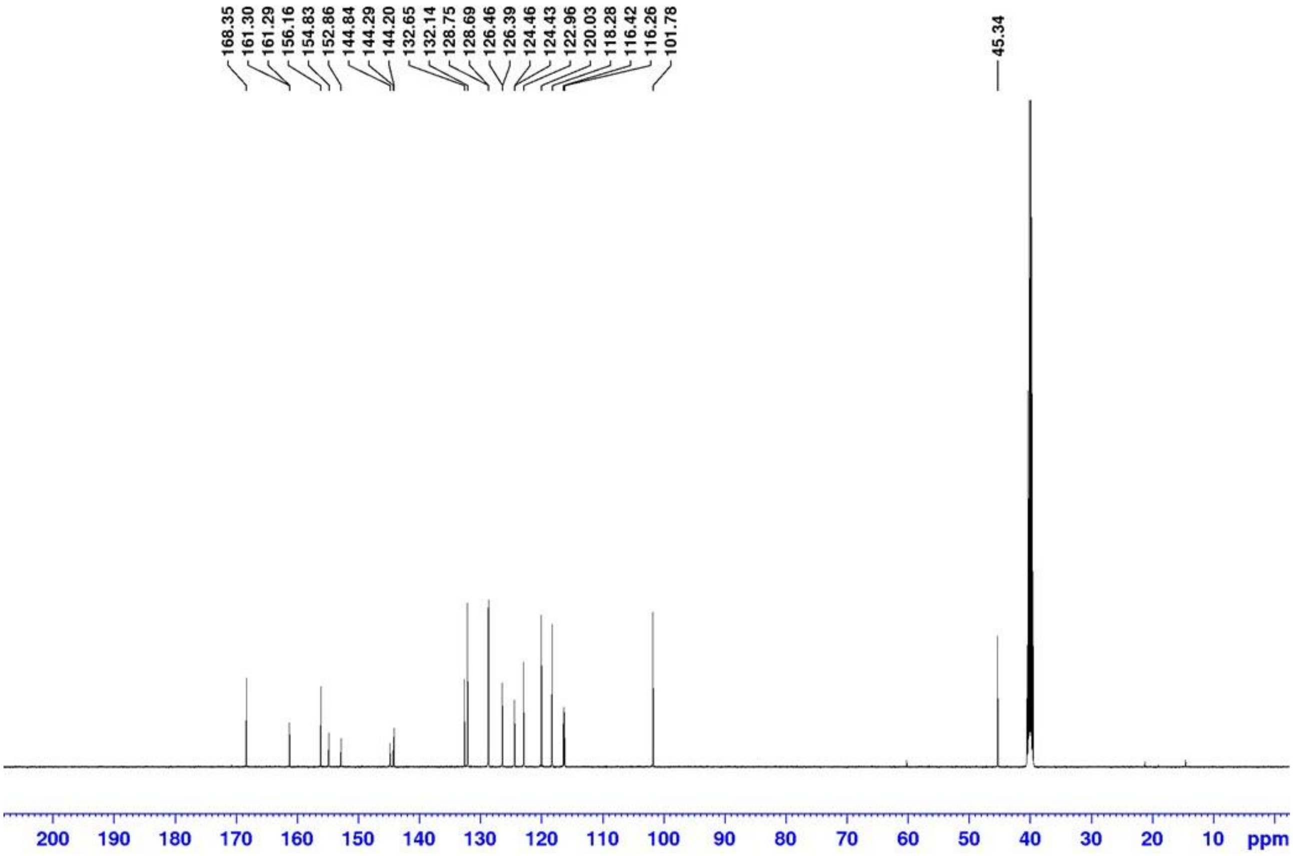

Figure S22. ^{13}C NMR spectrum (100 MHz, DMSO-d_6) of compound **6k**.

3-(3-fluoro-4-(4-(trifluoromethoxy)phenoxy)phenyl)-5-((2-nitro-1H-imidazol-1yl)methyl)isoxazole (6l)

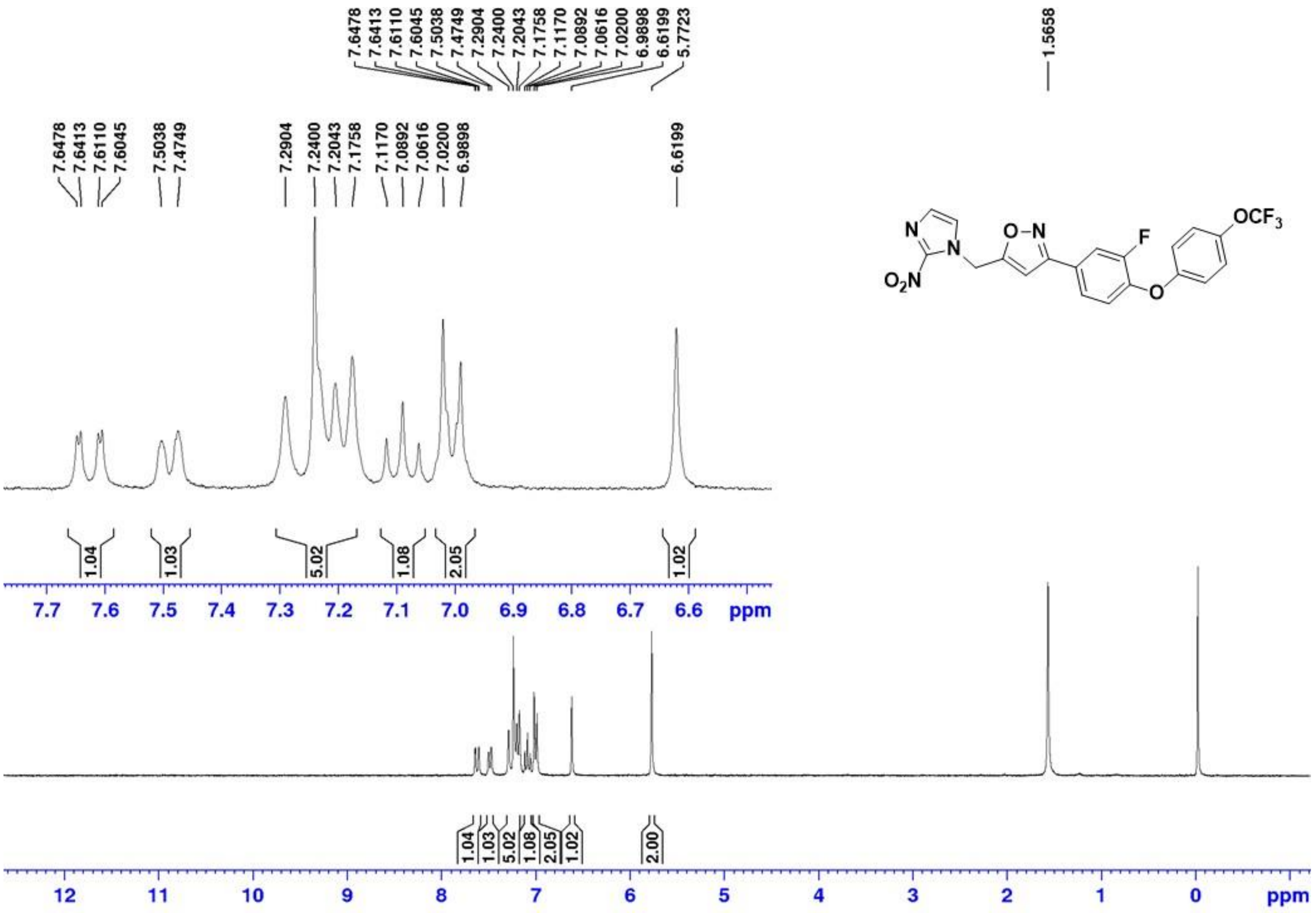

Figure S23. ^{1}H NMR spectrum (300 MHz, $CDCl_3$) of compound **6l**.

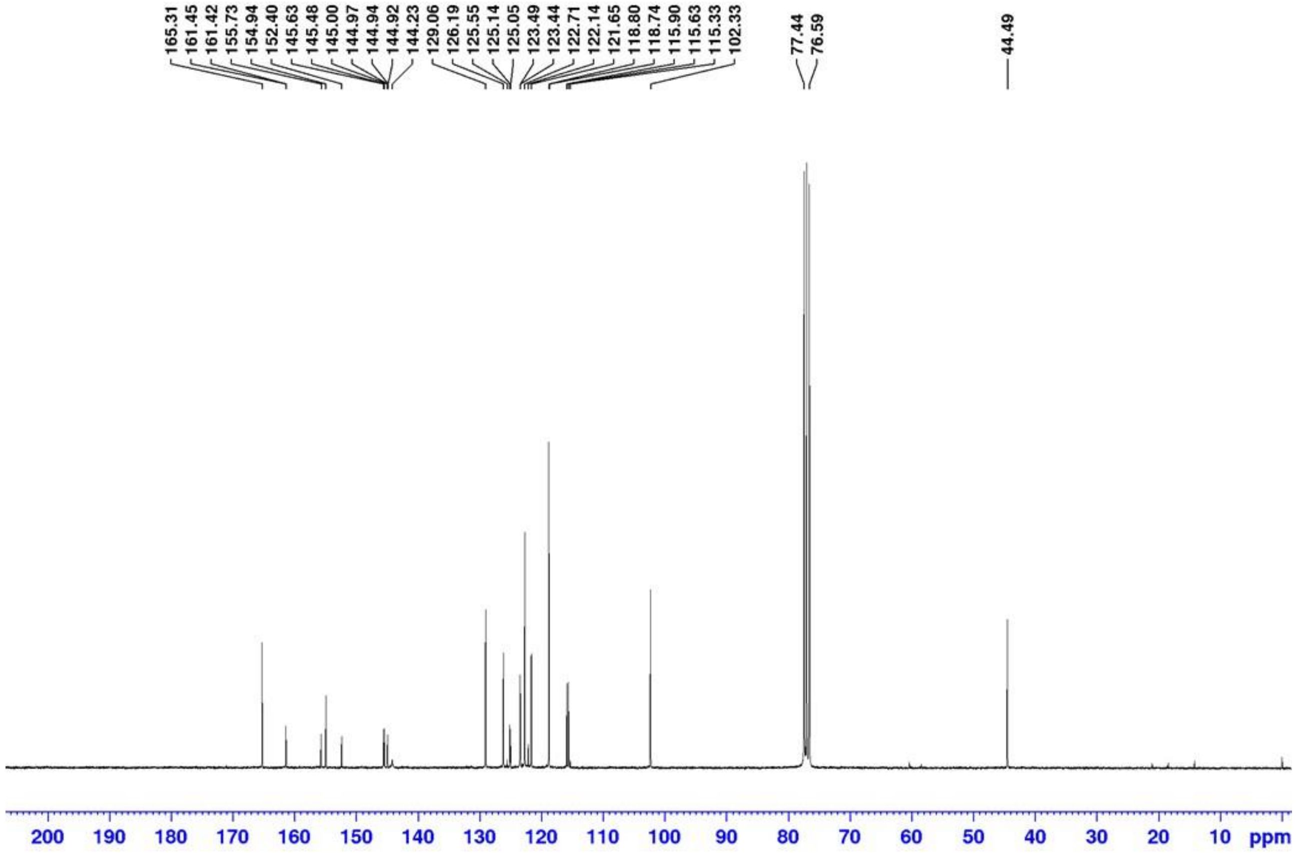

Figure S24. ^{13}C NMR spectrum (75 MHz, $CDCl_3$) of compound **6l**.

3-(3-fluoro-4-(p-tolyloxy)phenyl)-5-((2-nitro-1H-imidazol-1-yl)methyl)isoxazole (6m)

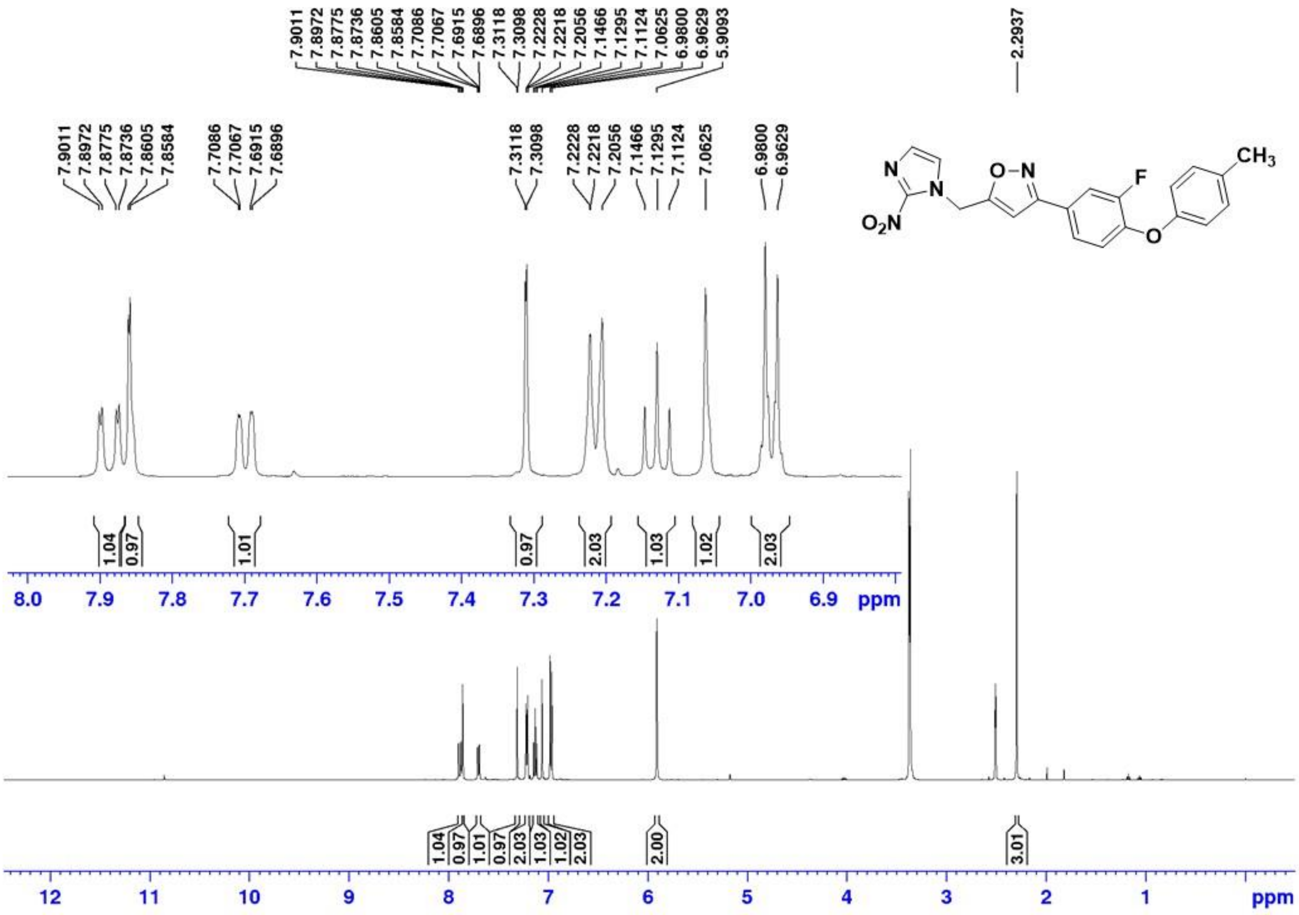

Figure S25. ^{1}H NMR spectrum (500 MHz, DMSO-d_6) of compound **6m**.

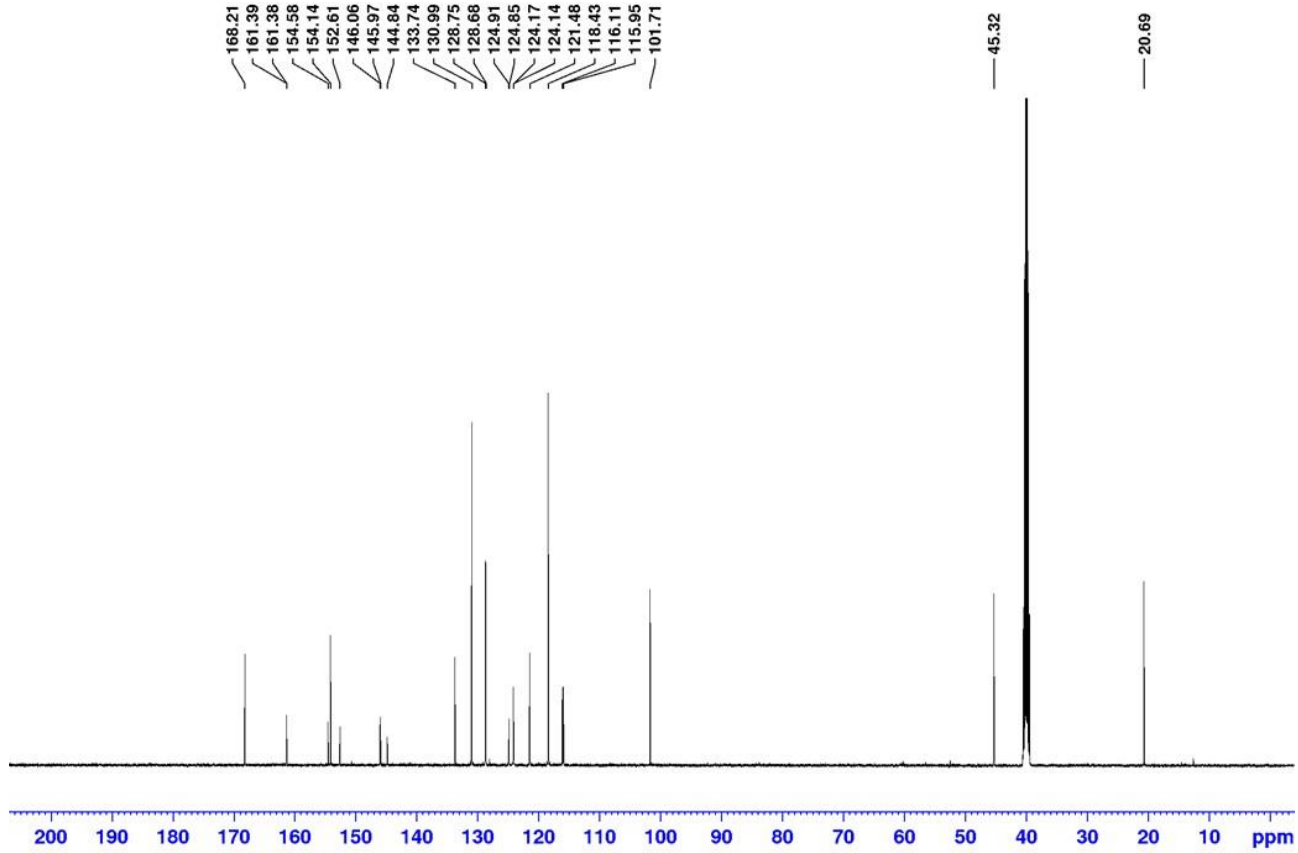

Figure S26. ^{13}C NMR spectrum (100 MHz, DMSO-d_6) of compound **6m.**

3-(3-fluoro-4-(4-methoxyphenoxy)phenyl)-5-((2-nitro-1H-imidazol-1-yl)methyl)isoxazole (6n)

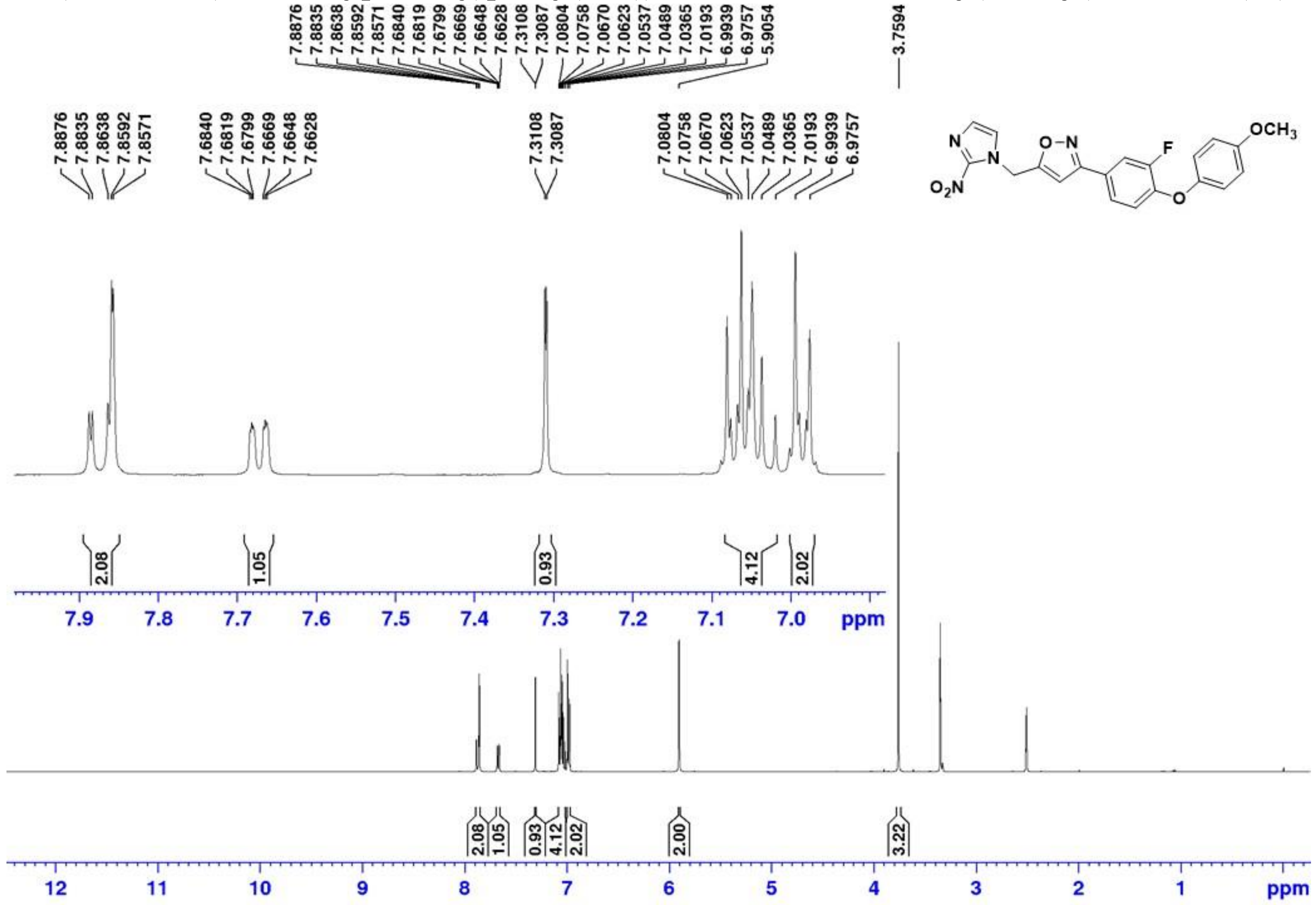

Figure S27. ^{1}H NMR spectrum (500 MHz, DMSO-d_6) of compound **6n**.

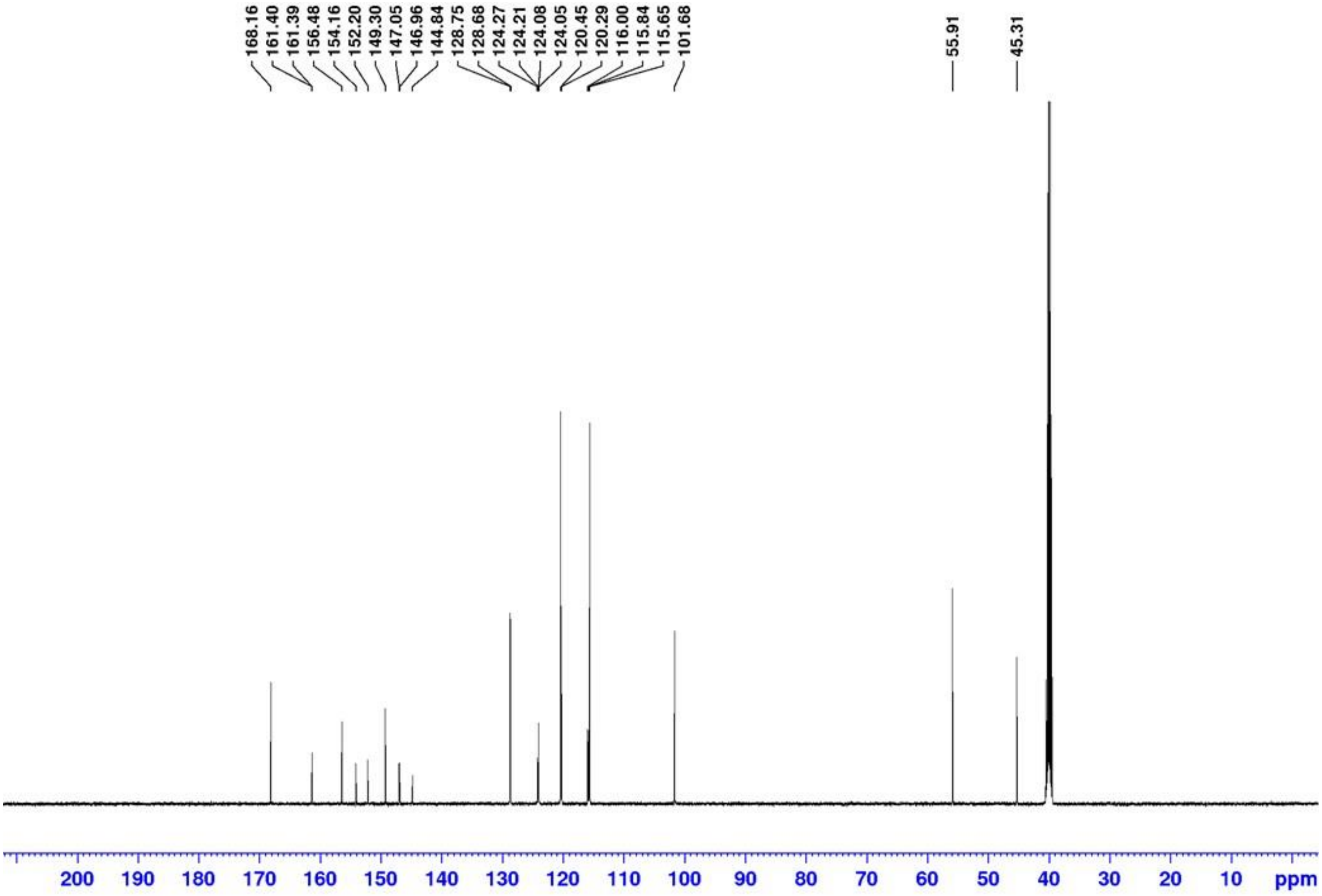

Figure S28. ^{13}C NMR spectrum (100 MHz, DMSO-d_6) of compound **6n**.

5-((2-nitro-1H-imidazol-1-yl)methyl)-3-(4-(phenylthio)phenyl)isoxazole (6o)

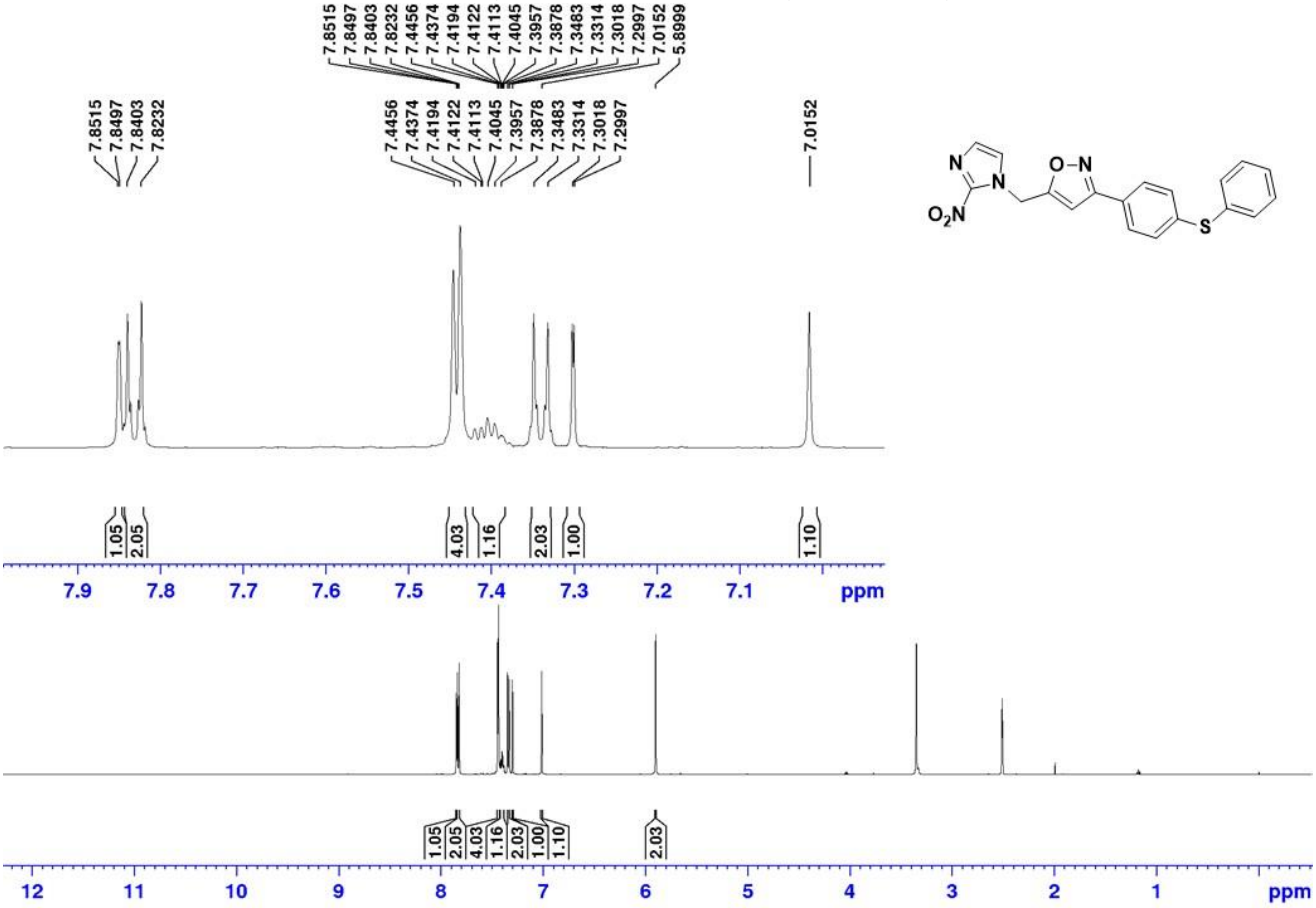

Figure S29. ¹H NMR spectrum (500MHz, DMSO-d_6) of compound **6o**.

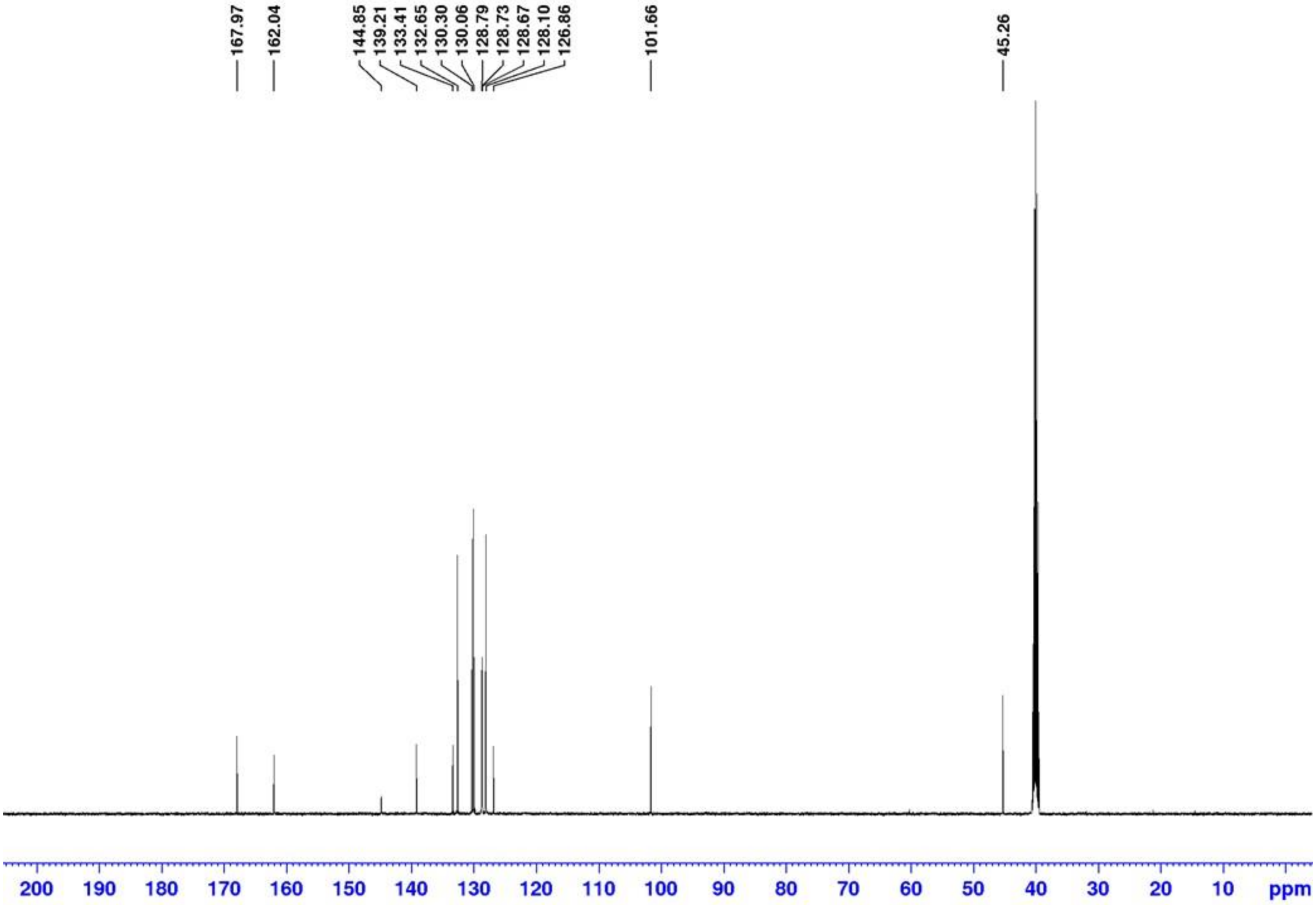

Figure S30. ¹³C NMR spectrum (100 MHz, DMSO-d_6) of compound **6o.**

3-(4-((4-chlorophenyl)thio)phenyl)-5-((2-nitro-1H-imidazol-1-yl)methyl)isoxazole (6p)

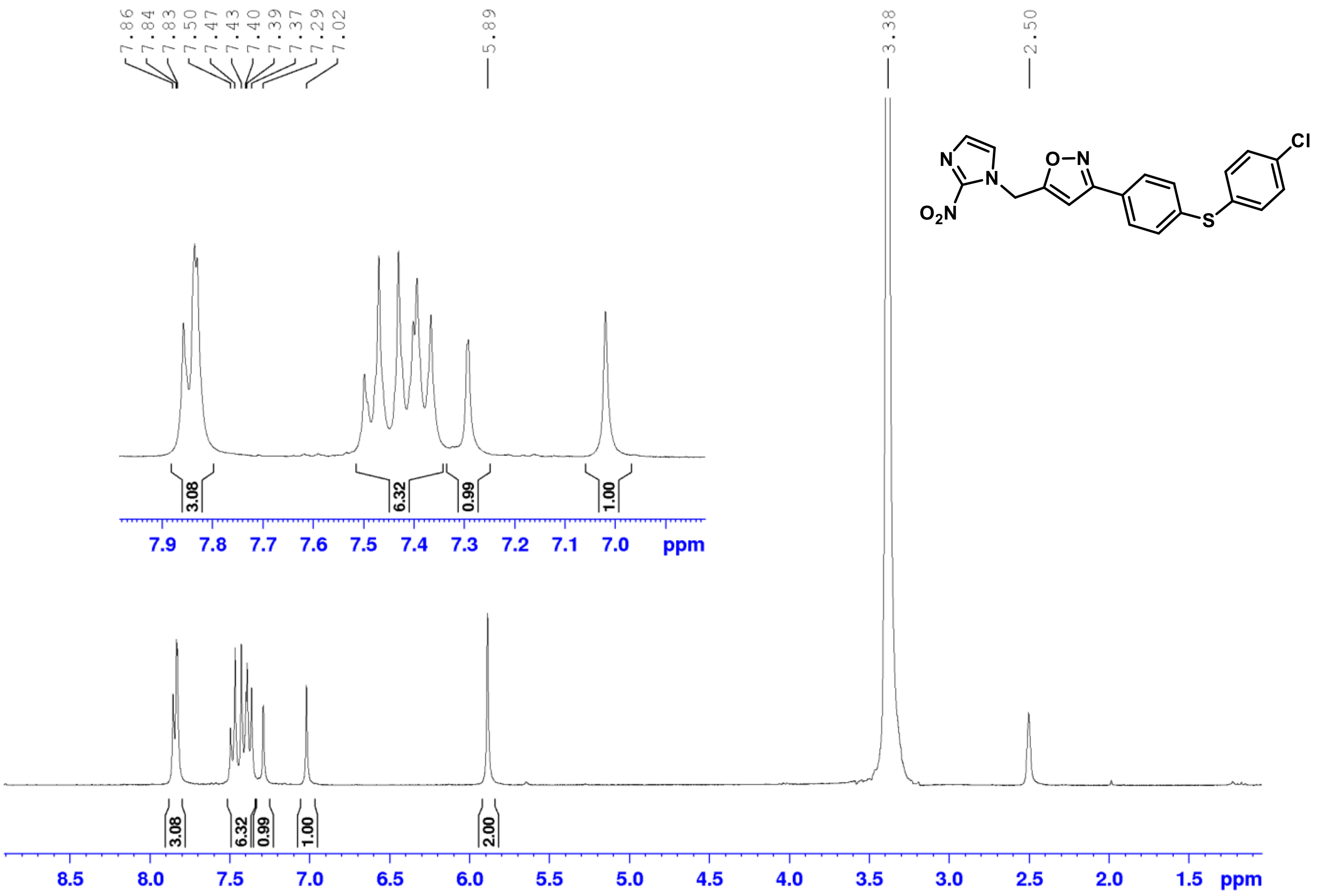

Figure S31. ^{1}H NMR spectrum (300MHz, DMSO-d_6) of compound **6p**.

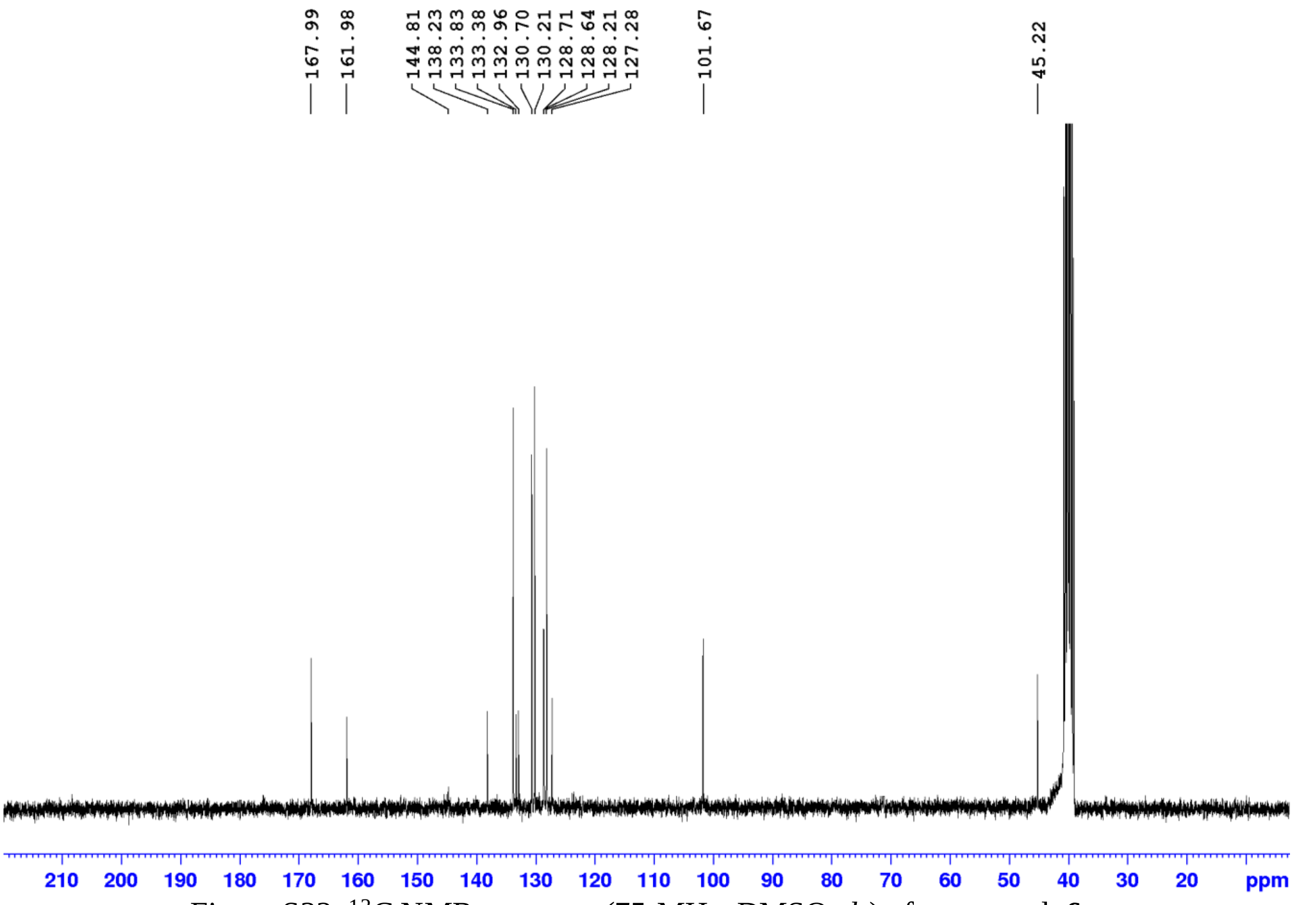

Figure S32. ^{13}C NMR spectrum (75 MHz, DMSO-d_6) of compound **6p.**

5-((2-nitro-1H-imidazol-1-yl)methyl)-3-(4-(p-tolylthio)phenyl)isoxazole (6q)

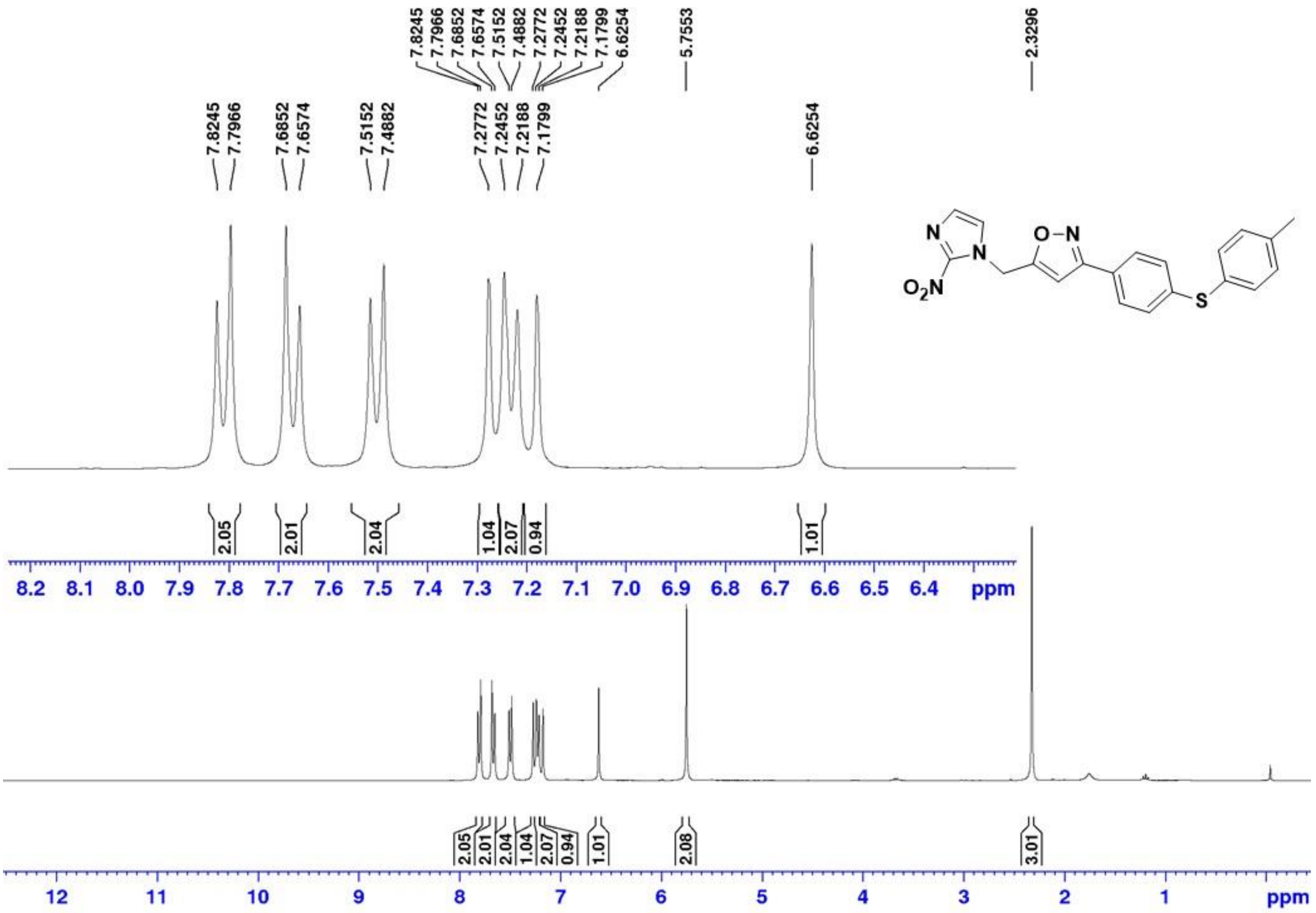

Figure S33. ^{1}H NMR spectrum (300 MHz, $CDCl_3$) of compound **6q**.

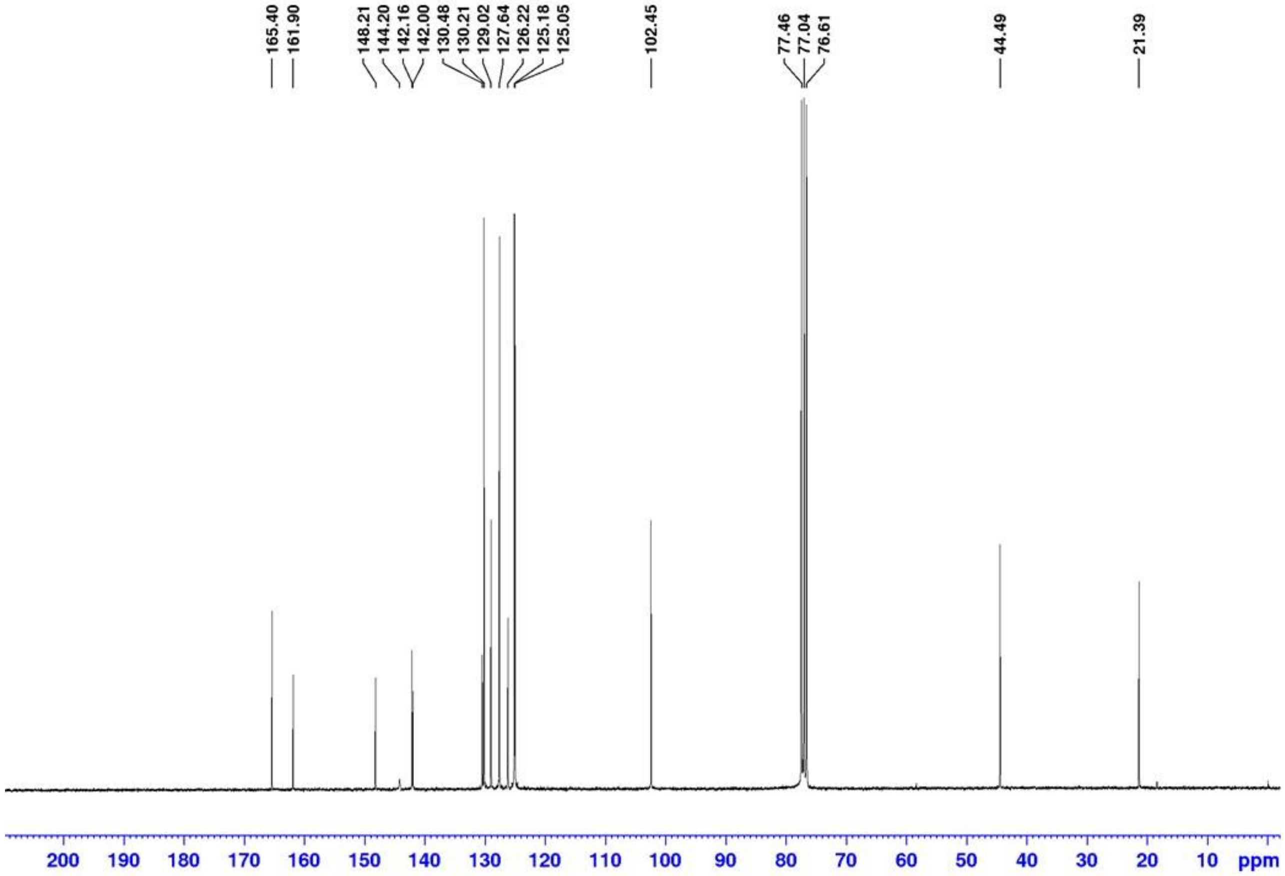

Figure S34. ^{13}C NMR spectrum (75 MHz, $CDCl_3$) of compound **6q**.
